# Supplementary material for: Structures and Bioactivities of Psolusosides B1, B2, J, K, L, M, N, O, P, and Q from the Sea Cucumber Psolus fabricii. The First Finding of Tetrasulfated Marine Low Molecular Weight Metabolites
Source: Mar Drugs. 2019 Nov 6;17(11):631. doi: 10.3390/md17110631 (PMC6891663; doi:10.3390/md17110631)
Supplement: Supplementary file 1 [file marinedrugs-17-00631-s001.pdf]

Supplementary Data Content Page

Title: Structures and bioactivities of psolusosides B_1, B_2, J, K, L, M, N, O, P and Q, triterpene glycosides from the sea cucumber *Psolus fabricii*. Novel tri- and unique tetrasulfated carbohydrate chains and the non-holostane aglycone with 7,8-epoxide

Authors: Alexandra S. Silchenko, Anatoly I. Kalinovsky, Sergey A. Avilov, Vladimir I. Kalinin*, Pelageya V. Andrijaschenko, Pavel S. Dmitrenok, Roman S. Popov, Ekaterina A. Chingizova

Address: [1]*G.B. Elyakov Pacific Institute of Bioorganic Chemistry, Far Eastern Branch of Russian Academy of Sciences, Pr. 100-let Vladivostoku 159, 690022 Vladivostok, Russia*

Correspondence: kalininv@piboc.dvo.ru; Tel.: +7-423-231-1168

Contents:

Table 1S. NMR data for the carbohydrate moiety of psolusosides B_1 (**1**) and B_2 (**2**).

Table 2S. NMR data for the aglycone moiety of psolusoside J (**3**).

Table 3S. NMR data for the aglycone moiety of psolusoside K (**4**), L (**5**), M (**6**), N (**7**), O (**8**), P (**9**), Q (**10**).

Figure 1. The ^{13}C NMR (176.04 MHz) spectrum of the aglycone moiety of **1** in C_5D_5N/D_2O (4/1)

Figure 2. The ^{1}H NMR (700.13 MHz) spectrum of the aglycone moiety of **1** in C_5D_5N/D_2O (4/1)

Figure 3. The HSQC (700.13 MHz) spectrum of the aglycone moiety of **1** in C_5D_5N/D_2O (4/1)

Figure 4. The ROESY (700.13 MHz) spectrum of the aglycone moiety of **1** in C_5D_5N/D_2O (4/1)

Figure 5. The ^{13}C NMR (176.04 MHz) spectrum of the aglycone moiety of **2** in C_5D_5N/D_2O (4/1)

Figure 6. The ^{1}H NMR (700.13 MHz) spectrum of the aglycone moiety of **2** in C_5D_5N/D_2O (4/1)

Figure 7. The HSQC (700.13 MHz) spectrum of the aglycone moiety of **2** in C_5D_5N/D_2O (4/1)

Figure 8. The ROESY (700.13 MHz) spectrum of the aglycone moiety of **2** in C_5D_5N/D_2O (4/1)

Figure 9. The ^{13}C NMR (176.04 MHz) spectrum of the carbohydrate moiety of **3** in C_5D_5N/D_2O (4/1)

Figure 10. The ^{1}H NMR (700.13 MHz) spectrum of the carbohydrate moiety of **3** in C_5D_5N/D_2O (4/1)

Figure 11. The COSY (700.13 MHz) spectrum of the carbohydrate moiety of **3** in C_5D_5N/D_2O (4/1)

Figure 12. The HSQC (700.13 MHz) spectrum of the carbohydrate moiety of **3** in C_5D_5N/D_2O (4/1)

Figure 13. The ROESY (500.13 MHz) spectrum of the carbohydrate moiety of **3** in C_5D_5N/D_2O (4/1)

Figure 14. 1D TOCSY (700.13 MHz) spectra of the monosaccharide residues in **3** in C_5D_5N/D_2O (4/1)

Figure 15. The ^{13}C NMR (176.04 MHz) spectrum of the carbohydrate moiety of **4** in C_5D_5N/D_2O (4/1)

Figure 16. The ^{1}H NMR (700.13 MHz) spectrum of the carbohydrate moiety of **4** in C_5D_5N/D_2O (4/1)

Figure 17. The COSY (700.13 MHz) spectrum of the carbohydrate moiety of **4** in C_5D_5N/D_2O (4/1)

Figure 18. The HSQC (700.13 MHz) spectrum of the carbohydrate moiety of **4** in C_5D_5N/D_2O (4/1)

Figure 19. The ROESY (500.13 MHz) spectrum of the carbohydrate moiety of **4** in C_5D_5N/D_2O (4/1)

Figure 20. 1D TOCSY (700.13 MHz) spectra of the monosaccharide residues in **4** in C_5D_5N/D_2O (4/1)

Figure 21. The ^{13}C NMR (176.04 MHz) spectrum of the carbohydrate moiety of **5** in C_5D_5N/D_2O (4/1)

Figure 22. The ^{1}H NMR (700.13 MHz) spectrum of the carbohydrate moiety of **5** in C_5D_5N/D_2O (4/1)

Figure 23. The COSY (700.13 MHz) spectrum of the carbohydrate moiety of **5** in C_5D_5N/D_2O (4/1)

Figure 24. The HSQC (700.13 MHz) spectrum of the carbohydrate moiety of **5** in C_5D_5N/D_2O (4/1)

Figure 25. The ROESY (500.13 MHz) spectrum of the carbohydrate moiety of **5** in C_5D_5N/D_2O (4/1)

Figure 26. 1D TOCSY (700.13 MHz) spectra of the monosaccharide residues in **5** in C_5D_5N/D_2O (4/1)

Figure 27. The ^{13}C NMR (176.04 MHz) spectrum of the carbohydrate moiety of **6** in C_5D_5N/D_2O (4/1)

Figure 28. The ^{1}H NMR (700.13 MHz) spectrum of the carbohydrate moiety of **6** in C_5D_5N/D_2O (4/1)

Figure 29. The COSY (700.13 MHz) spectrum of the carbohydrate moiety of **6** in C_5D_5N/D_2O (4/1)

Figure 30. The HSQC (700.13 MHz) spectrum of the carbohydrate moiety of **6** in C_5D_5N/D_2O (4/1)

Figure 31. The ROESY (500.13 MHz) spectrum of the carbohydrate moiety of **6** in C_5D_5N/D_2O (4/1)

Figure 32. 1D TOCSY (700.13 MHz) spectra of the monosaccharide residues in **6** in C_5D_5N/D_2O (4/1)

Figure 33. The ^{13}C NMR (176.04 MHz) spectrum of the carbohydrate moiety of **7** in C_5D_5N/D_2O (4/1)

Figure 34. The ^{1}H NMR (700.13 MHz) spectrum of the carbohydrate moiety of **7** in C_5D_5N/D_2O (4/1)

Figure 35. The COSY (700.13 MHz) spectrum of the carbohydrate moiety of **7** in C_5D_5N/D_2O (4/1)

Figure 36. The HSQC (700.13 MHz) spectrum of the carbohydrate moiety of **7** in C_5D_5N/D_2O (4/1)

Figure 37. The ROESY (500.13 MHz) spectrum of the carbohydrate moiety of **7** in C_5D_5N/D_2O (4/1)

Figure 38. 1D TOCSY (700.13 MHz) spectra of the monosaccharide residues in **7** in C_5D_5N/D_2O (4/1)

Figure 39. The ^{13}C NMR (176.04 MHz) spectrum of the carbohydrate moiety of **8** in C_5D_5N/D_2O (4/1)

Figure 40. The ^{1}H NMR (700.13 MHz) spectrum of the carbohydrate moiety of **8** in C_5D_5N/D_2O (4/1)

Figure 41. The COSY (700.13 MHz) spectrum of the carbohydrate moiety of **8** in C_5D_5N/D_2O (4/1)

Figure 42. The HSQC (700.13 MHz) spectrum of the carbohydrate moiety of **8** in C_5D_5N/D_2O (4/1)

Figure 43. The ROESY (500.13 MHz) spectrum of the carbohydrate moiety of **8** in C_5D_5N/D_2O (4/1)

Figure 44. 1D TOCSY (700.13 MHz) spectra of the monosaccharide residues in **8** in C_5D_5N/D_2O (4/1)

Figure 45. The ^{13}C NMR (176.04 MHz) spectrum of the carbohydrate moiety of **9** in C_5D_5N/D_2O (4/1)

Figure 46. The ^{1}H NMR (700.13 MHz) spectrum of the carbohydrate moiety of **9** in C_5D_5N/D_2O (4/1)

Figure 47. The COSY (700.13 MHz) spectrum of the carbohydrate moiety of **9** in C_5D_5N/D_2O (4/1)

Figure 48. The HSQC (700.13 MHz) spectrum of the carbohydrate moiety of **9** in C_5D_5N/D_2O (4/1)

Figure 49. The ROESY (500.13 MHz) spectrum of the carbohydrate moiety of **9** in C_5D_5N/D_2O (4/1)

Figure 50. 1D TOCSY (700.13 MHz) spectra of the monosaccharide residues in **9** in C_5D_5N/D_2O (4/1)

Figure 51. The ^{13}C NMR (176.04 MHz) spectrum of the carbohydrate moiety of **10** in C_5D_5N/D_2O (4/1)

Figure 52. The ^{1}H NMR (700.13 MHz) spectrum of the carbohydrate moiety of **10** in C_5D_5N/D_2O (4/1)

Figure 53. The COSY (700.13 MHz) spectrum of the carbohydrate moiety of **10** in C_5D_5N/D_2O (4/1)

Figure 54. The HSQC (700.13 MHz) spectrum of the carbohydrate moiety of **10** in C_5D_5N/D_2O (4/1)

Figure 55. The ROESY (500.13 MHz) spectrum of the carbohydrate moiety of **10** in C_5D_5N/D_2O (4/1)

Figure 56. 1D TOCSY (700.13 MHz) spectra of the monosaccharide residues in **10** in C_5D_5N/D_2O (4/1)

Figure 57. ESI-MS (–) and (+) spectra of psolusoside B_1 (**1**)

Figure 58. ESI-MS (–) spectra of psolusoside B_2 (**2**)

Figure 59. ESI-MS (–) spectra of psolusoside J (**3**)

Figure 60. ESI-MS (–) spectra of psolusoside K (**4**)

Figure 61. ESI-MS (–) spectra of psolusoside L (**5**)

Figure 62. ESI-MS (–) spectra of psolusoside M (**6**)

Figure 63. ESI-MS (–) spectra of psolusoside N (**7**)

Figure 64. ESI-MS (–) spectra of psolusoside O (**8**)

Figure 65. ESI-MS (–) spectra of psolusoside P (**9**)

Figure 66. ESI-MS (–) spectra of psolusoside Q (**10**)

Table 1S. ^{13}C and ^{1}H NMR chemical shifts and HMBC and ROESY correlations of carbohydrate moiety of psolusosides B$_1$ (**1**) and B$_2$ (**2**). ^{a} Recorded at 176.04 MHz in C_5D_5N/D_2O (4/1). ^{b} Bold = interglycosidic positions. ^{c} Italic = sulphate position. ^{d} Recorded at 700.13 MHz in C_5D_5N/D_2O (4/1). Multiplicity by 1D TOCSY.

Atom	δ_C mult.$^{a, b, c}$	δ_H mult.^{d} (J in Hz)	HMBC	ROESY
Xyl1 (1→C-3)				
1	104.8 CH	4.56 (d, 7.3)	C: 3; C: 5 Xyl1	H-3; H-3, 5 Xyl1
2	**81.0** CH	4.01 (t, 8.0)	C: 1 Glc2; C: 1, 3 Xyl1	H-1 Glc2
3	75.2 CH	4.20 (t, 8.8)	C: 2, 4 Xyl1	H-1 Xyl1
4	**78.6** CH	4.08 (dt, 5.6; 9.6)	C: 1 Clc4; C: 5 Xyl1	H-1 Glc4
5	63.6 CH_2	4.43 (dd, 5.2; 12.1)	C: 1, 3, 4 Xyl1	
		3.73 (brt, 11.3)	C: 1 Xyl1	H-1 Xyl1
Glc2 (1→2Xyl1)				
1	104.1 CH	5.11 (d, 7.8)	C: 2 Xyl1; C: 5 Glc2	H-2 Xyl1; H-3, 5 Glc2
2	75.1 CH	3.82 (t, 7.8)	C: 1, 3 Glc2	
3	75.2 CH	3.96 (t, 8.7)	C: 2, 4 Glc2	H-1 Glc2
4	**82.2** CH	3.87 (t, 8.7)	C: 1 Glc3; C: 5, 6 Glc2	H-1 Glc3
5	75.9 CH	3.70 (dt, 2.9; 9.7)		H-1, 3 Glc2
6	61.4 CH_2	4.30 (dd, 2.9; 12.3)		
		4.25 (dd, 4.6; 12.2)	C: 4, 5 Glc2	
Glc3 (1→4Glc2)				
1	104.5 CH	4.81 (d, 7.9)	C: 4 Glc2	H-4 Glc2; H-5 Glc3
2	74.1 CH	3.79 (t, 9.2)	C: 1, 3, 4 Glc3	H-4 Glc3
3	**76.8** CH	4.07 (t, 9.2)	C: 2, 4 Glc3	H-1 Glc3
4	70.7 CH	3.90 (t, 9.2)	C: 3, 5, 6 Glc3	
5	75.5 CH	4.03 (dd, 4.6; 10.1)		H-1 Glc3
6	*67.5* CH_2	5.01 (d, 10.1)	C: 4 Glc3	
		4.64 (dd, 6.7; 11.1)	C: 5 Glc3	
Glc4 (1→4Xyl1)				
1	100.9 CH	4.92 (d, 7.8)	C: 4 Xyl1	H-4 Xyl1; H-3, 5 Glc4
2	*80.6* CH	4.74 (t, 8.9)	C: 1, 3 Glc4	
3	76.8 CH	4.28 (t, 8.9)	C: 2, 4 Glc4	H-1, 5 Glc4
4	70.7 CH	3.90 (t, 8.9)	C: 3, 5, 6 Glc4	
5	77.4 CH	3.84 (dd, 4.6; 10.2)	C: 4 Glc4	H-1 Glc4
6	61.8 CH_2	4.32 (dd, 2.5; 12.1)	C: 4 Glc4	
		4.01 (dd, 6.4; 12.1)	C: 4, 5 Glc4	

Table 2S. ^{13}C and ^{1}H NMR chemical shifts and HMBC and ROESY correlations of aglycone moiety of psolusoside J (**3**). [a] Recorded at 176.04 MHz in C_5D_5N/D_2O (4/1). [b] Recorded at 700.13 MHz in C_5D_5N/D_2O (4/1). [c] Recorded at 500.13 MHz in C_5D_5N/D_2O (4/1).

Position	δ_C mult.[a]	δ_H mult. (*J* in Hz)[b]	HMBC	ROESY[c]
1	35.4 CH_2	1.35 m		H-3, H-5, H-11, H-19
2	26.7 CH_2	2.06 m		
		1.89 m		
3	89.2 CH	3.24 dd (3.8; 11.8)	C: 30, 1 Xyl1	
4	39.2 C			
5	48.1 CH	0.92 dd (4.3; 11.6)		H-1, H-3, H-31
6	23.1 CH_2	1.91 m		H-19, H-31
7	121.7 CH	5.63 m		
8	143.9 C			
9	46.9 CH	3.54 brd (15.2)		H-19
10	35.7 C			
11	22.2 CH_2	1.80 m		H-1
		1.53 m		H-32
12	29.4 CH_2	2.19 brdd (5.8; 8.8)	C: 13, 18	H-17, H-21, H-32
13	56.6 C			
14	45.6 C			
15	51.8 CH_2	2.65 d (15.9)	C: 13, 16, 32	H-7, H-32
		2.32 d (16.1)	C: 14, 16, 32	H-7
16	213.8 C			
17	63.3 CH	2.87 s	C: 12, 13, 16, 18, 20, 21	H-12, H-21, H-22, H-32
18	179.0 C			
19	23.8 CH_3	1.10 s	C: 1, 9, 10	H-1, H-2, H-6, H-9
20	83.6 C			
21	26.0 CH_3	1.45 s	C: 17, 20, 22	H-12, H-17, H-22
22	38.1 CH_2	1.71 m		H-17, H-21
		1.56 m		
23	22.0 CH_2	1.71 m		
		1.43 m		
24	37.7 CH_2	1.90 m	C: 25, 26	H-26
25	145.4 C			
26	110.3 CH_2	4.70 brs	C: 24, 27	H-27
		4.69 brs	C: 24, 27	H-27
27	22.0 CH_3	1.63 s	C: 24, 25, 26	
30	17.1 CH_3	1.07 s	C: 3, 4, 5, 31	H-2, H-6, H-6 Glc2
31	28.5 CH_3	1.20 s	C: 3, 4, 5, 30	H-3, H-5, H-6, H-1 Xyl1
32	31.7 CH_3	1.16 s	C: 8, 13, 14, 15	H-7, H-11, H-12, H-15, H-17

Table 3S. ^{13}C and ^{1}H NMR chemical shifts and HMBC and ROESY correlations of aglycone moiety of psolusosides K–Q (**4**–**10**). [a] Recorded at 176.04 MHz in C_5D_5N/D_2O (4/1). [b] Recorded at 700.13 MHz in C_5D_5N/D_2O (4/1). [c] Recorded at 500.13 MHz in C_5D_5N/D_2O (4/1).

Position	δ_C mult.[a]	δ_H mult. (*J* in Hz)[b]	HMBC	ROESY[c]
1	36.2 CH_2	1.89 m		H-11, H-19
		1.52 m		H-3, H-5, H-11
2	27.0 CH_2	2.30 m		
		2.02 m		H-19, H-30
3	88.7 CH	3.31 (dd, 4.8; 11.6)	C: 4, 30, 31, C-1 Xyl1	H-1, H-5, H-31, H-1 Xyl1
4	39.6 C			
5	52.8 CH	0.99 (brd, 12.0)	C: 4, 10, 19, 30	H-1, H-3, H-7, H-31
6	21.0 CH_2	1.75 m		
		1.57 m		H-19, H-30
7	28.4 CH_2	1.62 m		H-15
		1.27 m		H-5, H-32
8	38.6 CH	3.29 m	C: 9	H-15, H-19
9	151.2 C			
10	39.8 C			
11	110.9 CH	5.35 m	C: 8, 13	H-1
12	32.0 CH_2	2.48 m	C: 14	H-21
		2.52 m	C: 9, 11, 13, 14, 18	H-17, H-32
13	55.6 C			
14	41.9 C			
15	51.8 CH_2	2.39 d (15.6)	C: 13, 16, 17, 32	H-7, H-32
		2.23 d (15.6)	C: 14, 16, 32	H-8
16	212.9 C			
17	61.2 CH	2.80 s	C: 12, 13, 16, 18, 20, 21	H-12, H-21, H-22, H-32
18	175.8 C			
19	21.9 CH_3	1.43 s	C: 1, 5, 9, 10	H-1, H-2, H-8, H-30
20	82.9 C			
21	26.6 CH_3	1.40 s	C: 17, 20, 22	H-12, H-17, H-22
22	38.3 CH_2	1.81 m		H-12, H-17, H-21
		1.66 m		
23	22.1 CH_2	1.81 m		
		1.53 m		
24	37.8 CH_2	1.99 m	C: 25, 26, 27	H-27
25	145.4 C			
26	110.3 CH_2	4.78 brs	C: 24, 25, 27	H-27
27	22.2 CH_3	1.70 s	C: 24, 25, 26	
30	16.5 CH_3	1.11 s	C: 3, 4, 5, 31	H-2, H-6, H-19
31	27.9 CH_3	1.31 s	C: 3, 4, 5, 30	H-3, H-5, H-6, H-1 Xyl1
32	20.5 CH_3	0.92 s	C: 8, 13, 14, 15	H-7, H-12, H-15, H-17

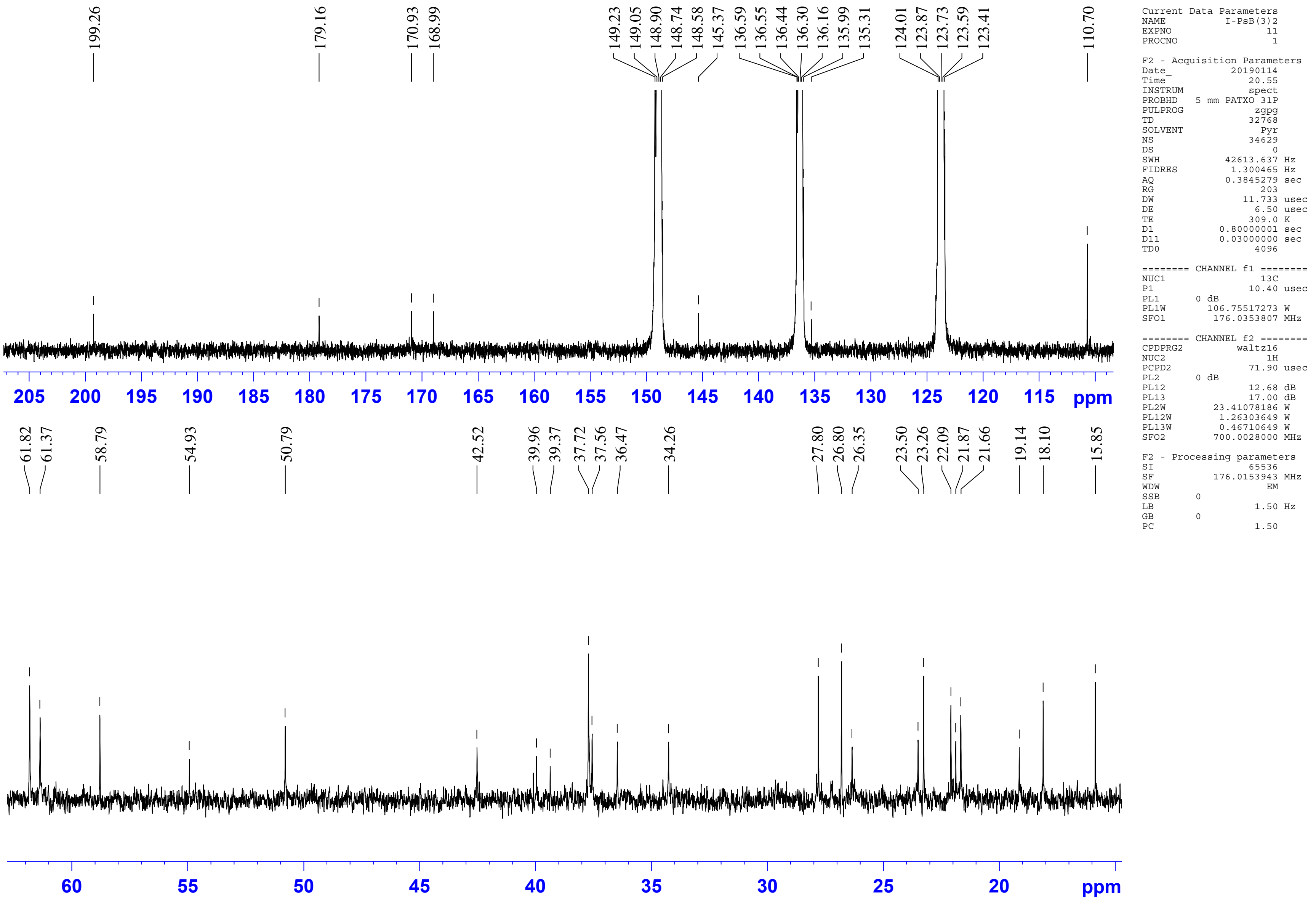

Figure 1. The ^{13}C NMR (176.04 MHz) spectrum of the aglycone moiety of **1** in C_5D_5N/D_2O (4/1).

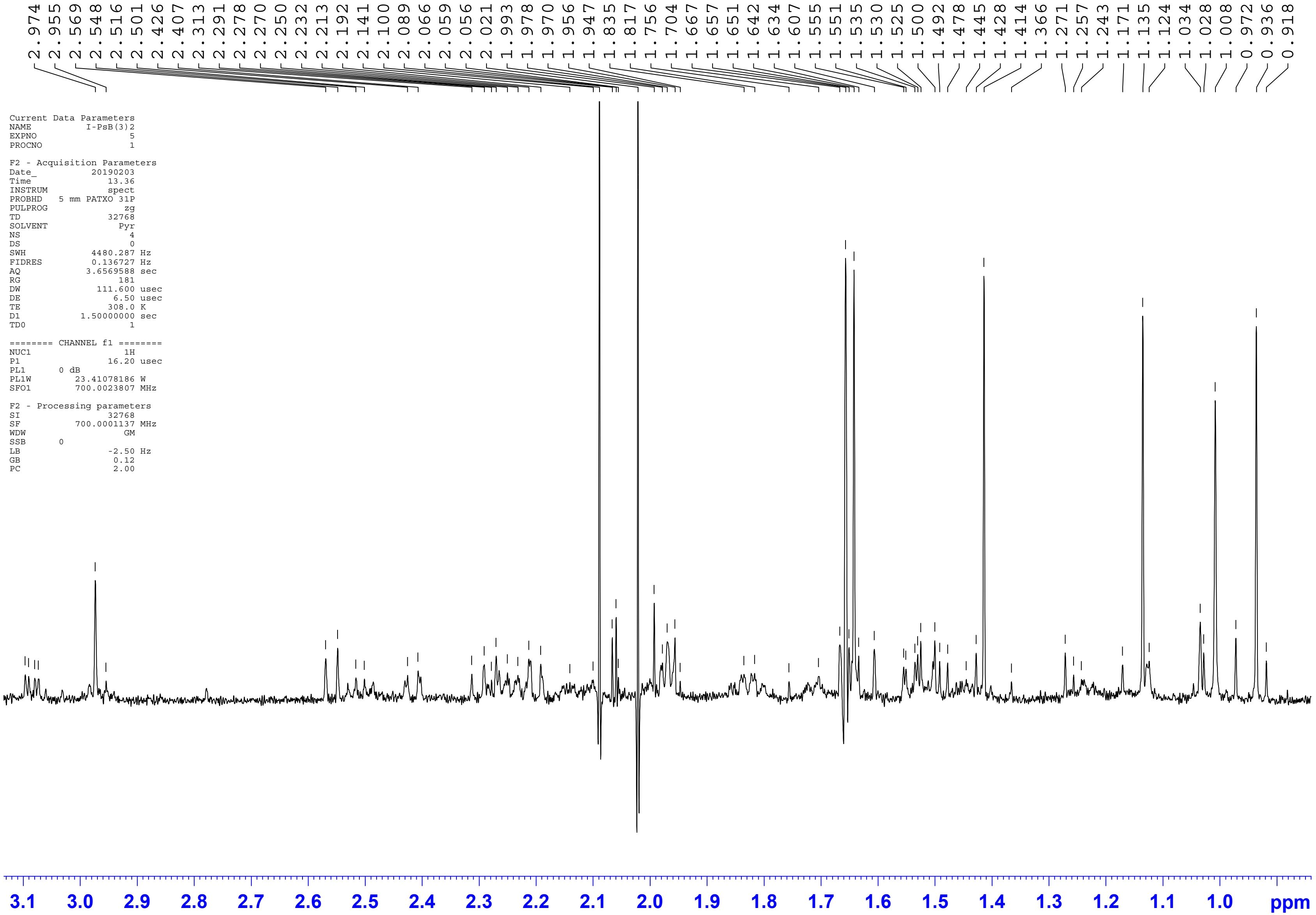

Figure 2. The ^{1}H NMR (700.13 MHz) spectrum of the aglycone moiety of **1** in C_5D_5N/D_2O (4/1).

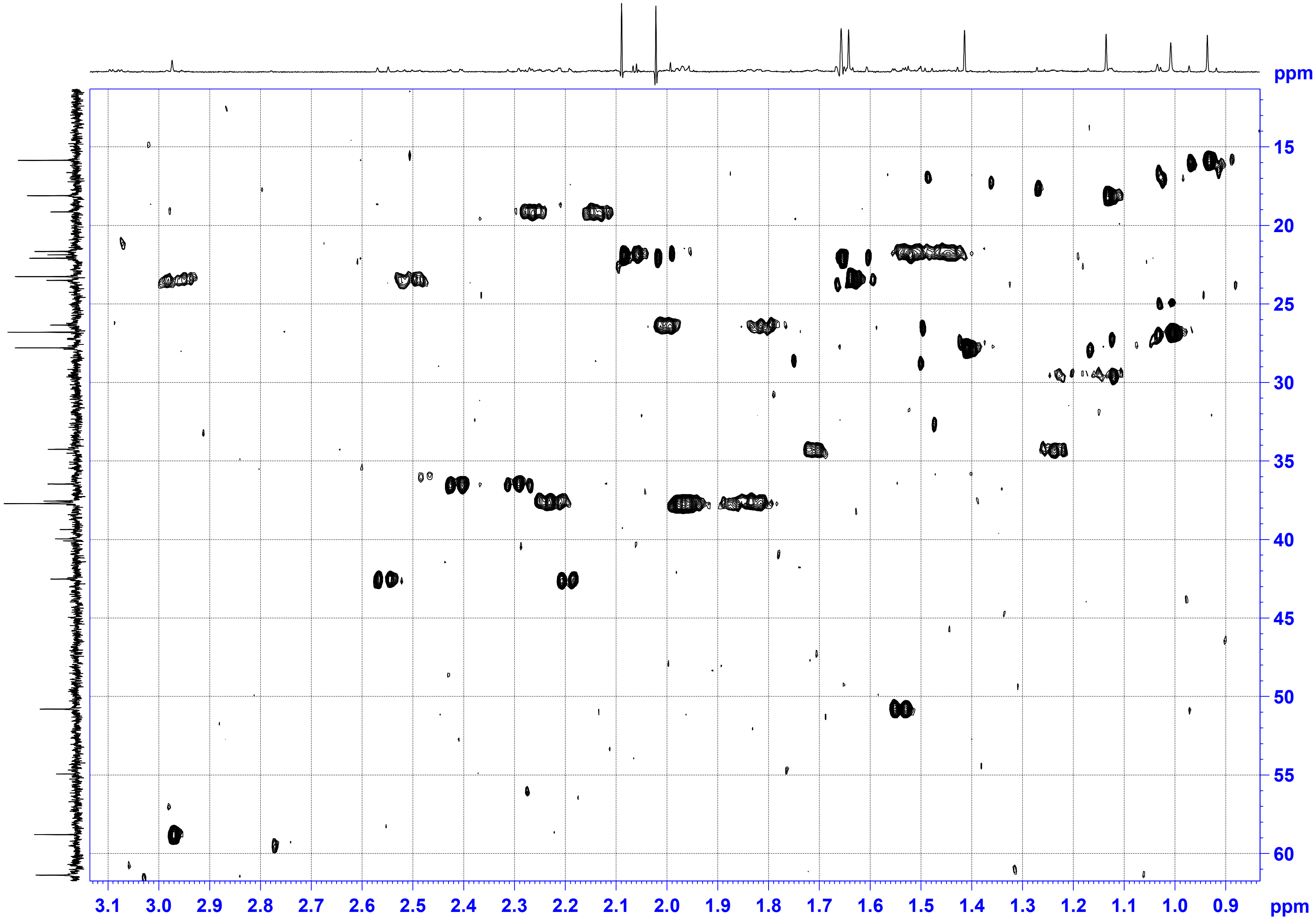

Figure 3. The HSQC (700.13 MHz) spectrum of the aglycone moiety of **1** in C_5D_5N/D_2O (4/1).

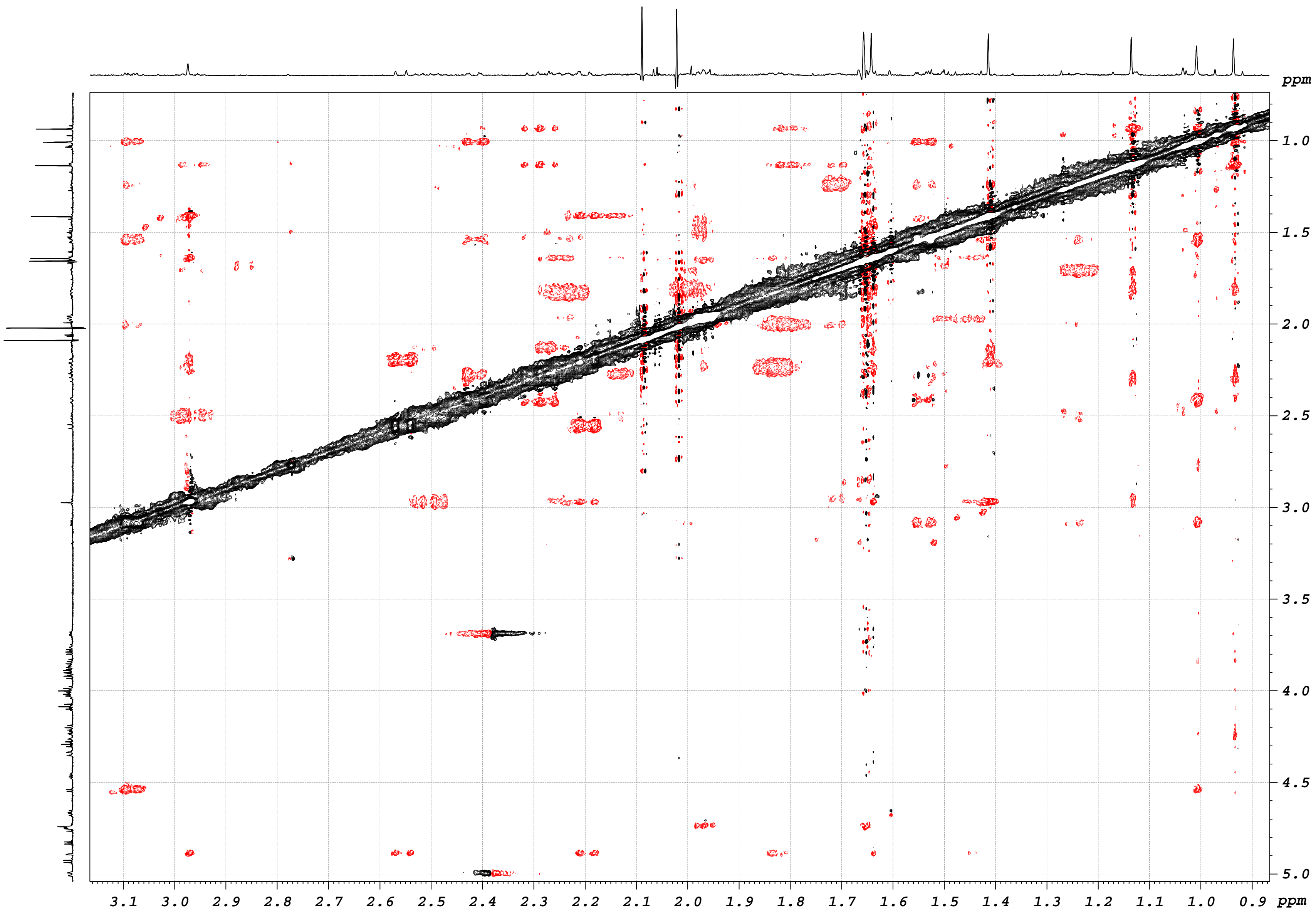

Figure 4. The ROESY (700.13 MHz) spectrum of the aglycone moiety of **1** in C_5D_5N/D_2O (4/1).

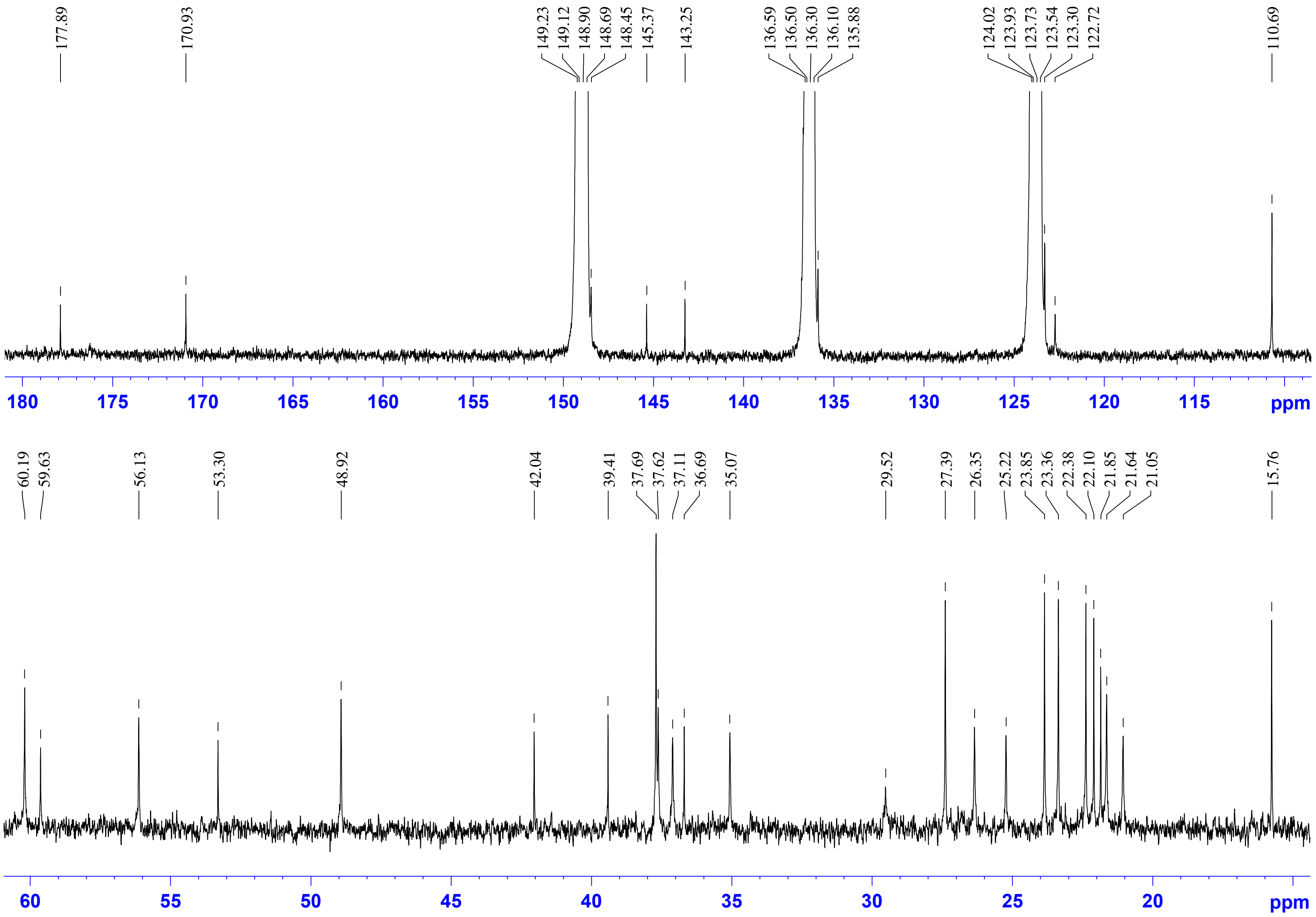

Figure 5. The ^{13}C NMR (176.04 MHz) spectrum of the aglycone moiety of **2** in C_5D_5N/D_2O (4/1).

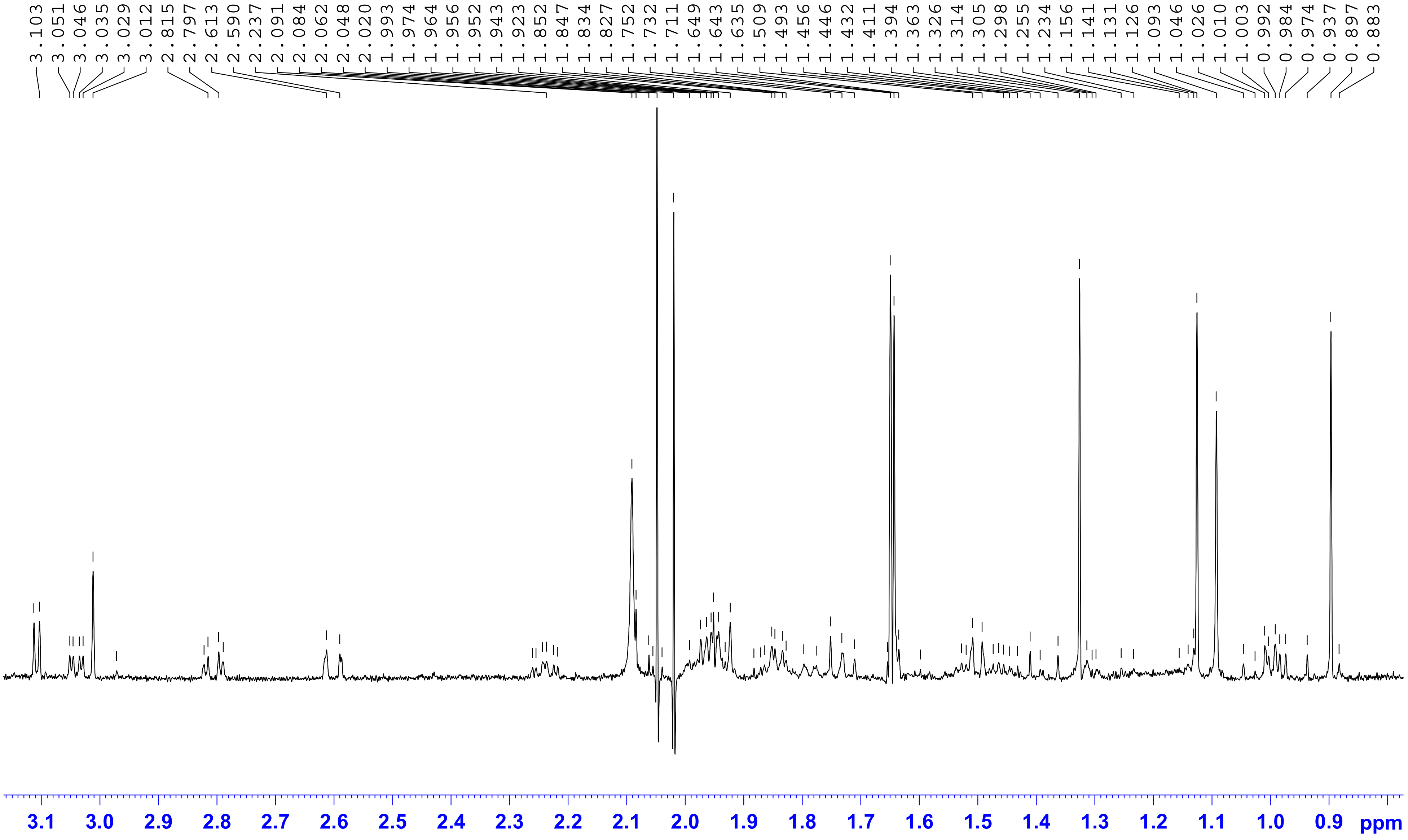

Figure 6. The ^{1}H NMR (700.13 MHz) spectrum of the aglycone moiety of **2** in C_5D_5N/D_2O (4/1).

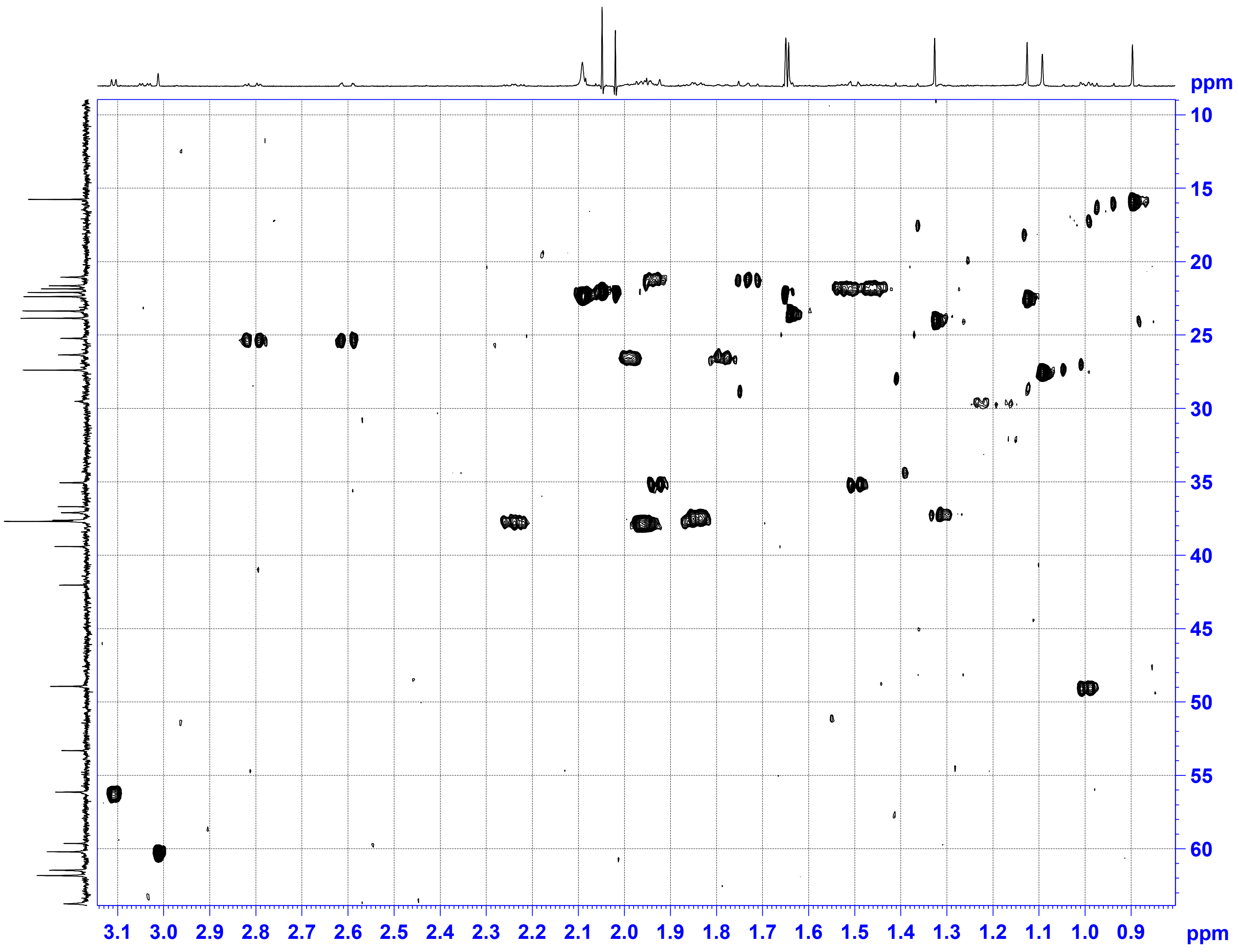

Figure 7. The HSQC (700.13 MHz) spectrum of the aglycone moiety of **2** in C_5D_5N/D_2O (4/1).

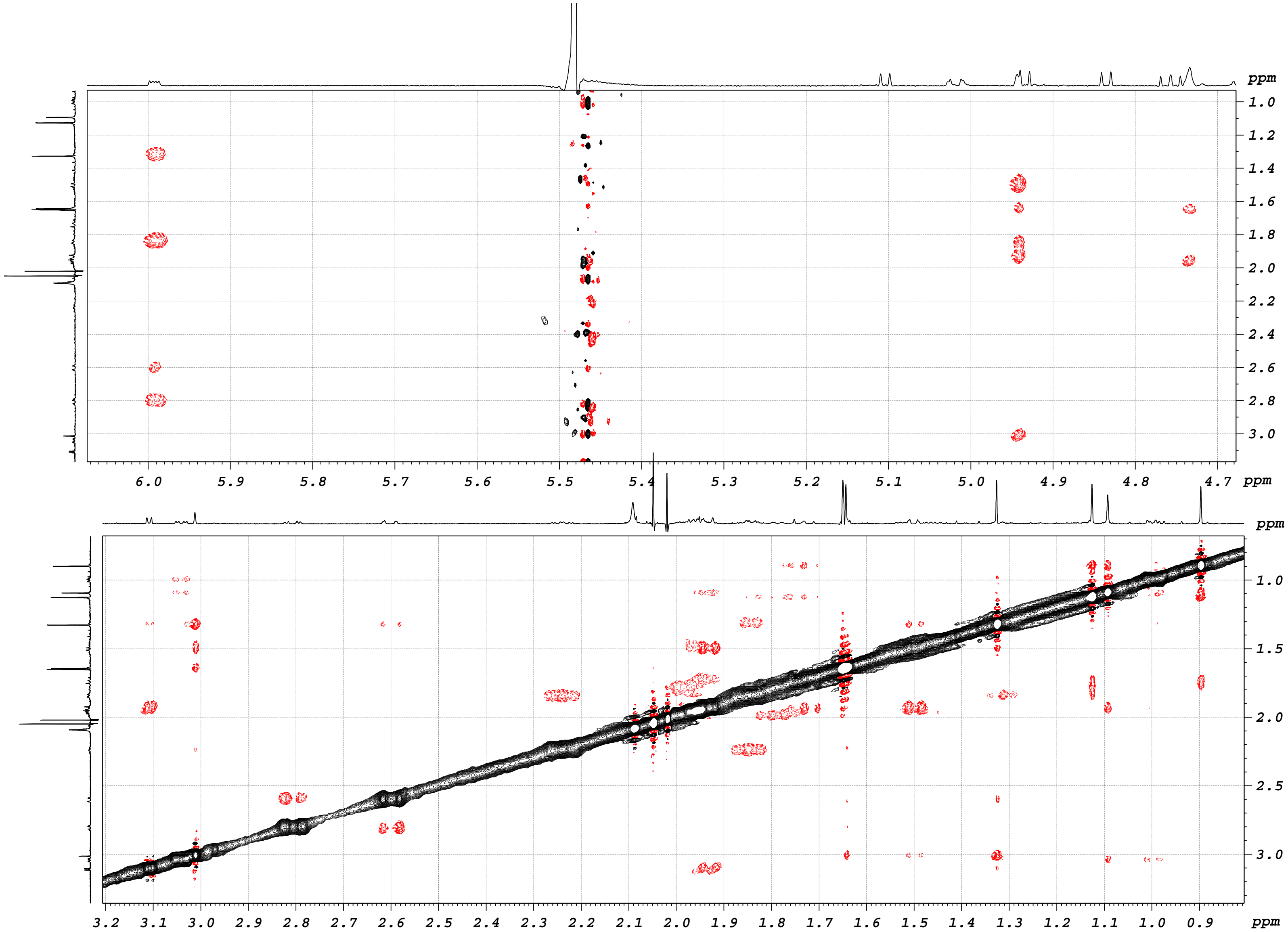

Figure 8. The ROESY (700.13 MHz) spectrum of the aglycone moiety of **2** in C_5D_5N/D_2O (4/1).

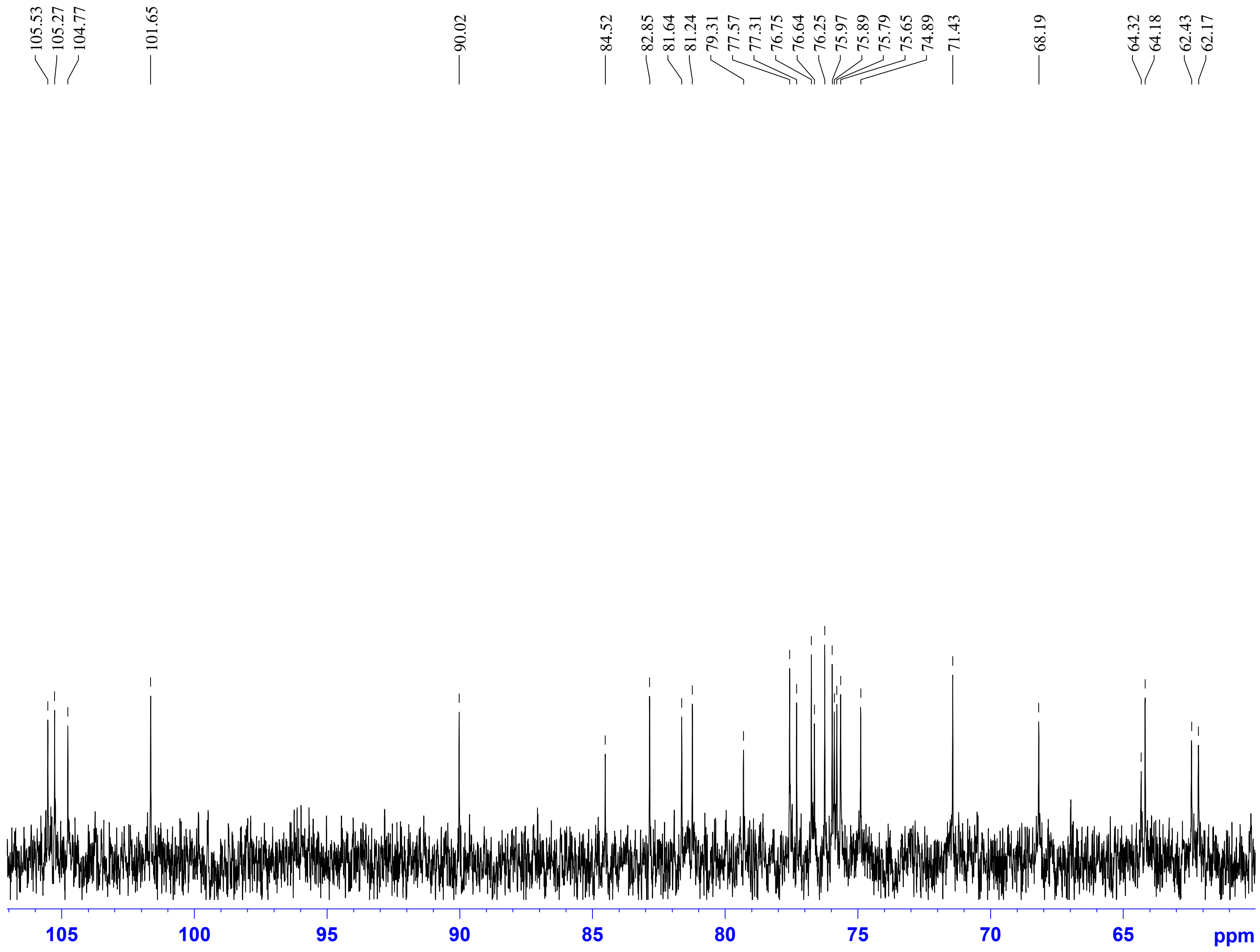

Figure 9. The ^{13}C NMR (176.04 MHz) spectrum of the carbohydrate moiety of **3** in C_5D_5N/D_2O (4/1).

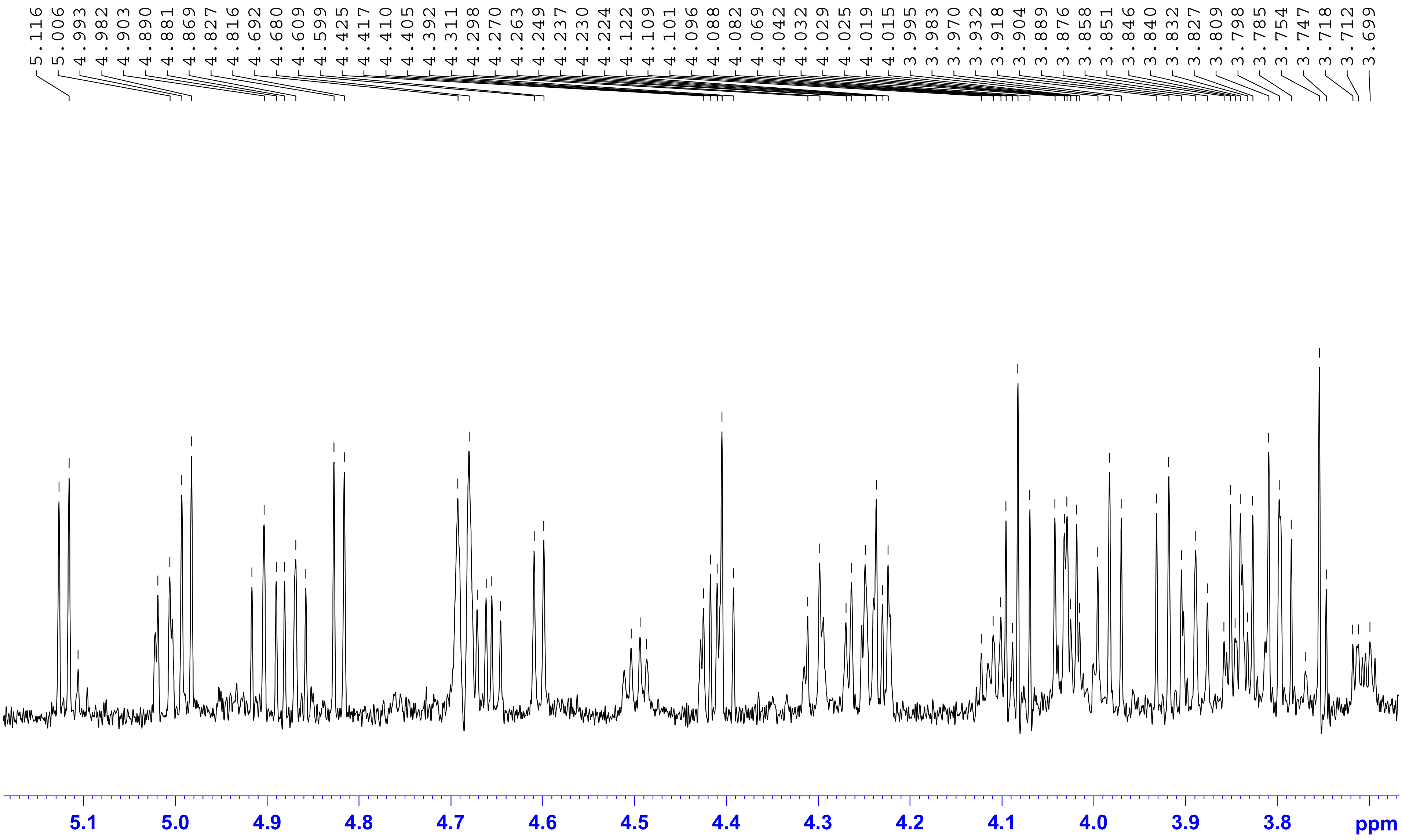

Figure 10. The ^{1}H NMR (700.13 MHz) spectrum of the carbohydrate moiety of **3** in C_5D_5N/D_2O (4/1).

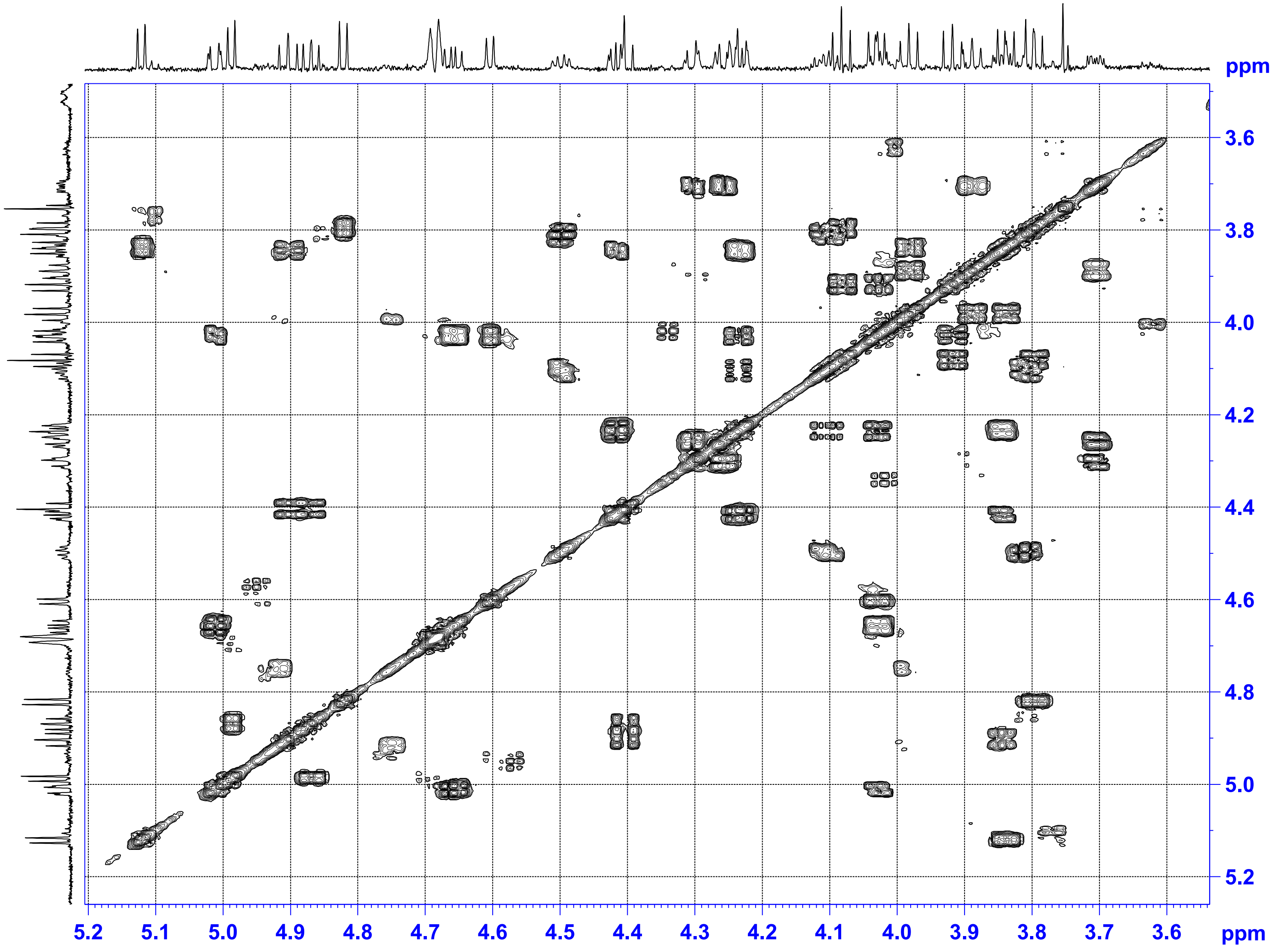

Figure 11. The COSY (700.13 MHz) spectrum of the carbohydrate moiety of **3** in C_5D_5N/D_2O (4/1).

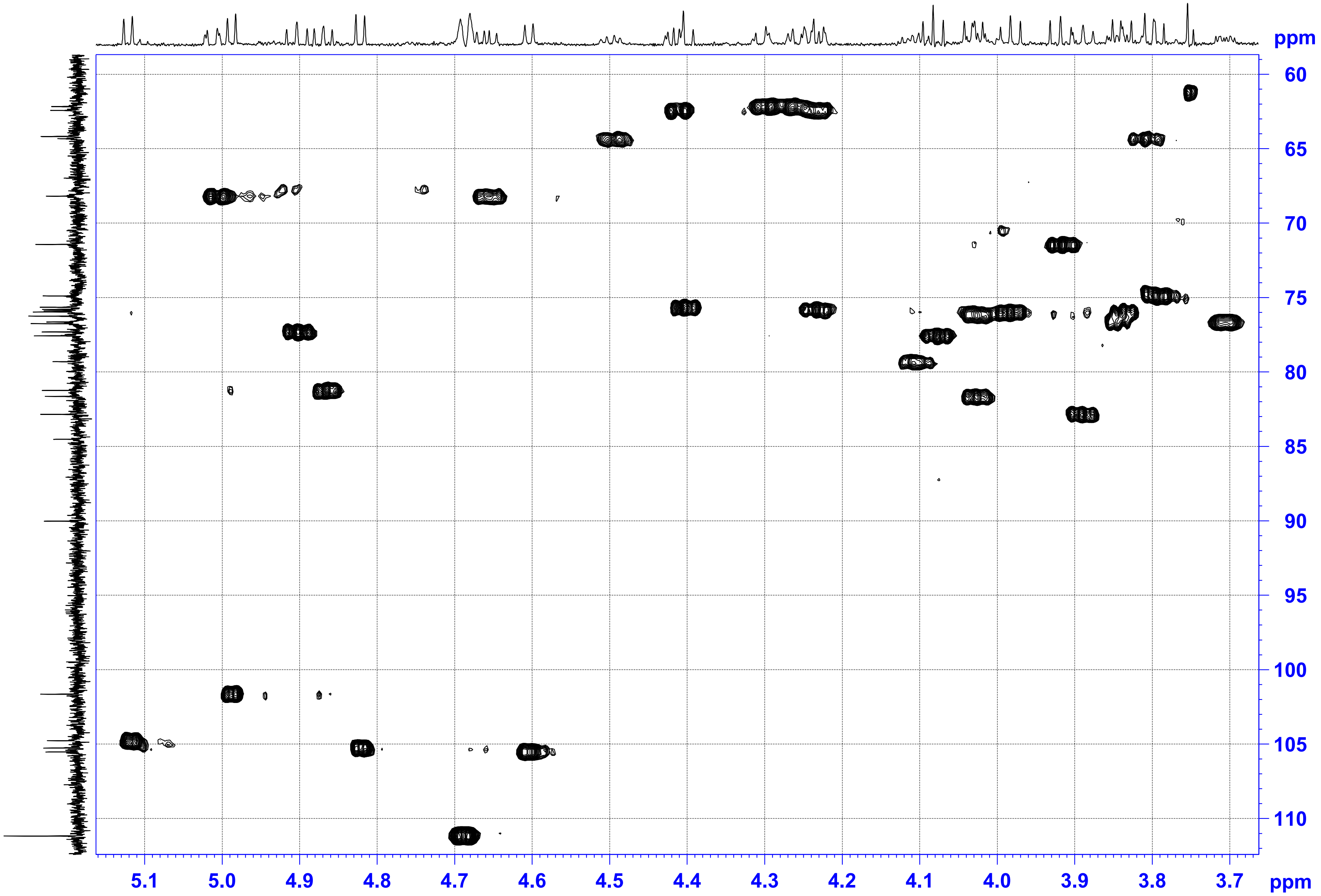

Figure 12. The HSQC (700.13 MHz) spectrum of the carbohydrate moiety of **3** in C_5D_5N/D_2O (4/1).

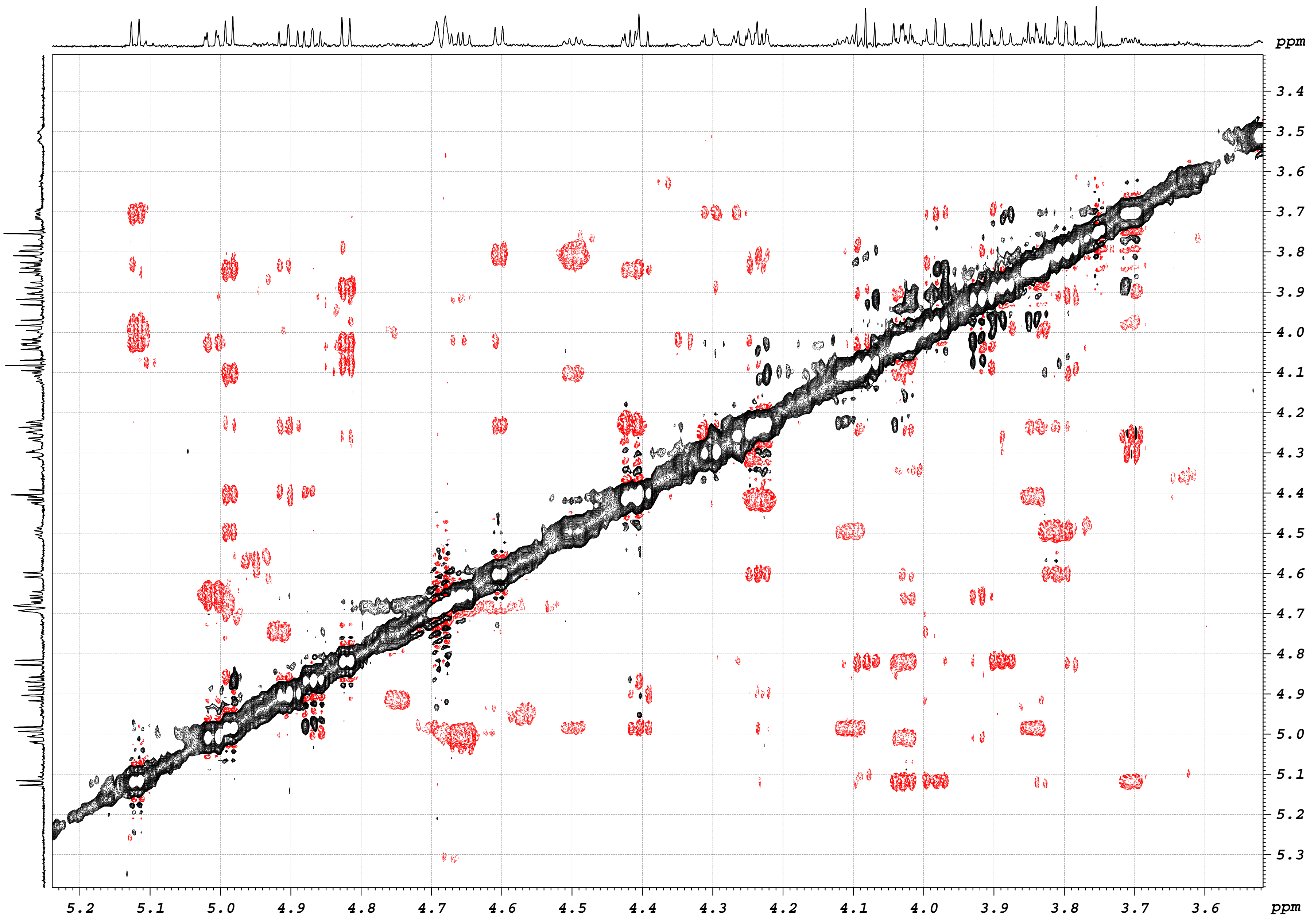

Figure 13. The ROESY (500.13 MHz) spectrum of the carbohydrate moiety of **3** in C_5D_5N/D_2O (4/1).

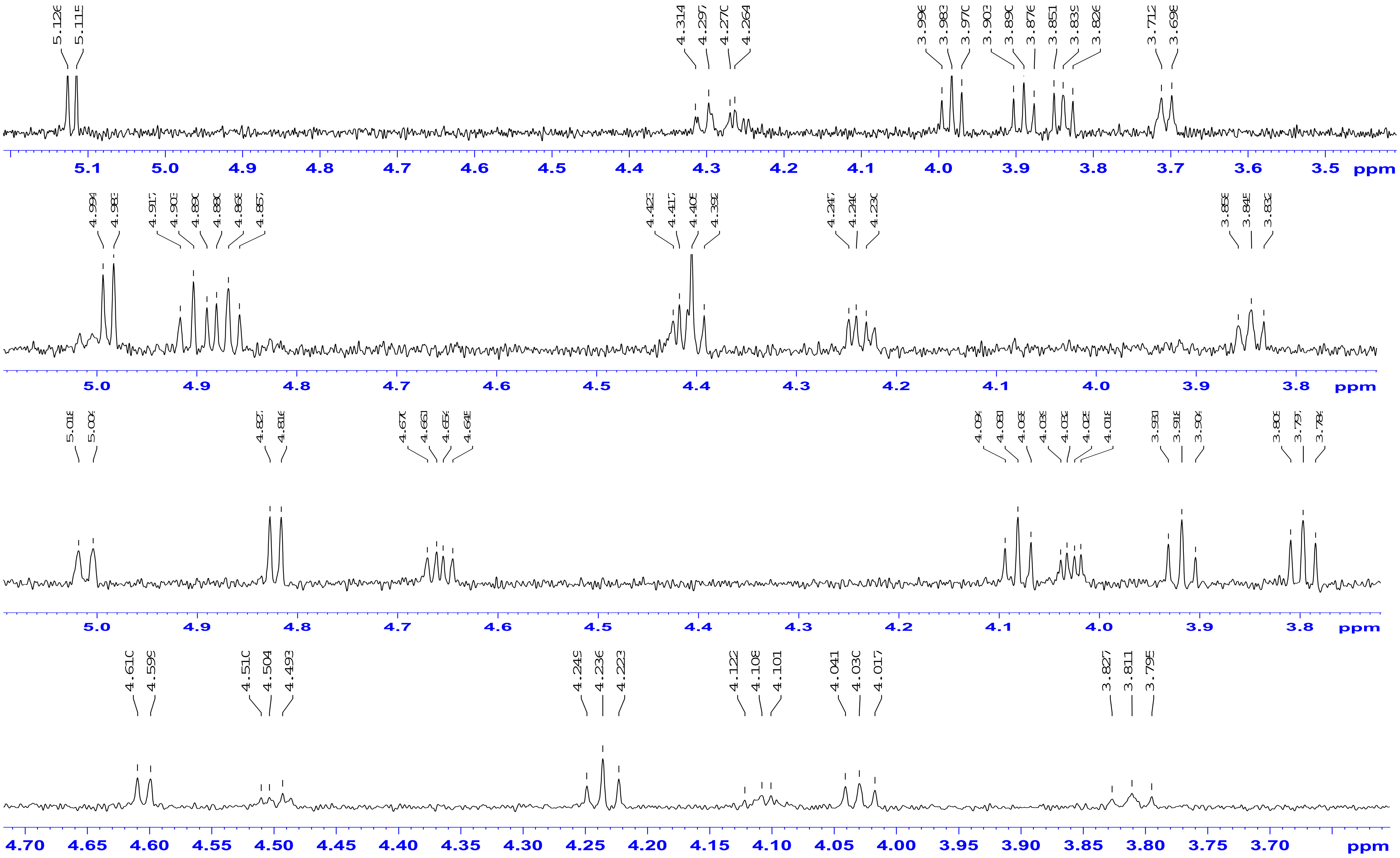

Figure 14. 1D TOCSY (700.13 MHz) spectra of the carbohydrate moiety of **3** in C_5D_5N/D_2O (4/1).

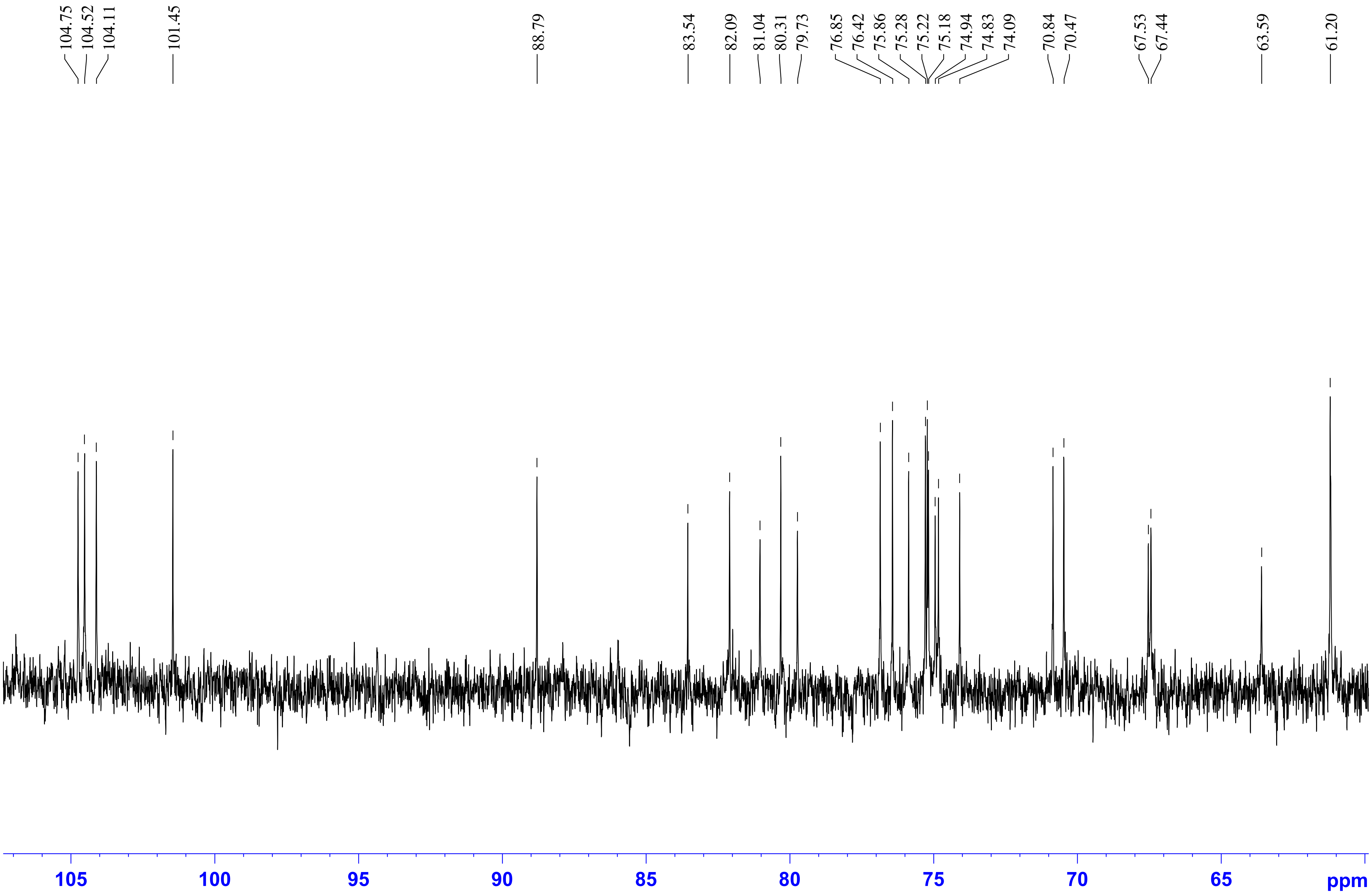

Figure 15. The ^{13}C NMR (176.04 MHz) spectrum of the carbohydrate moiety of **4** in C_5D_5N/D_2O (4/1).

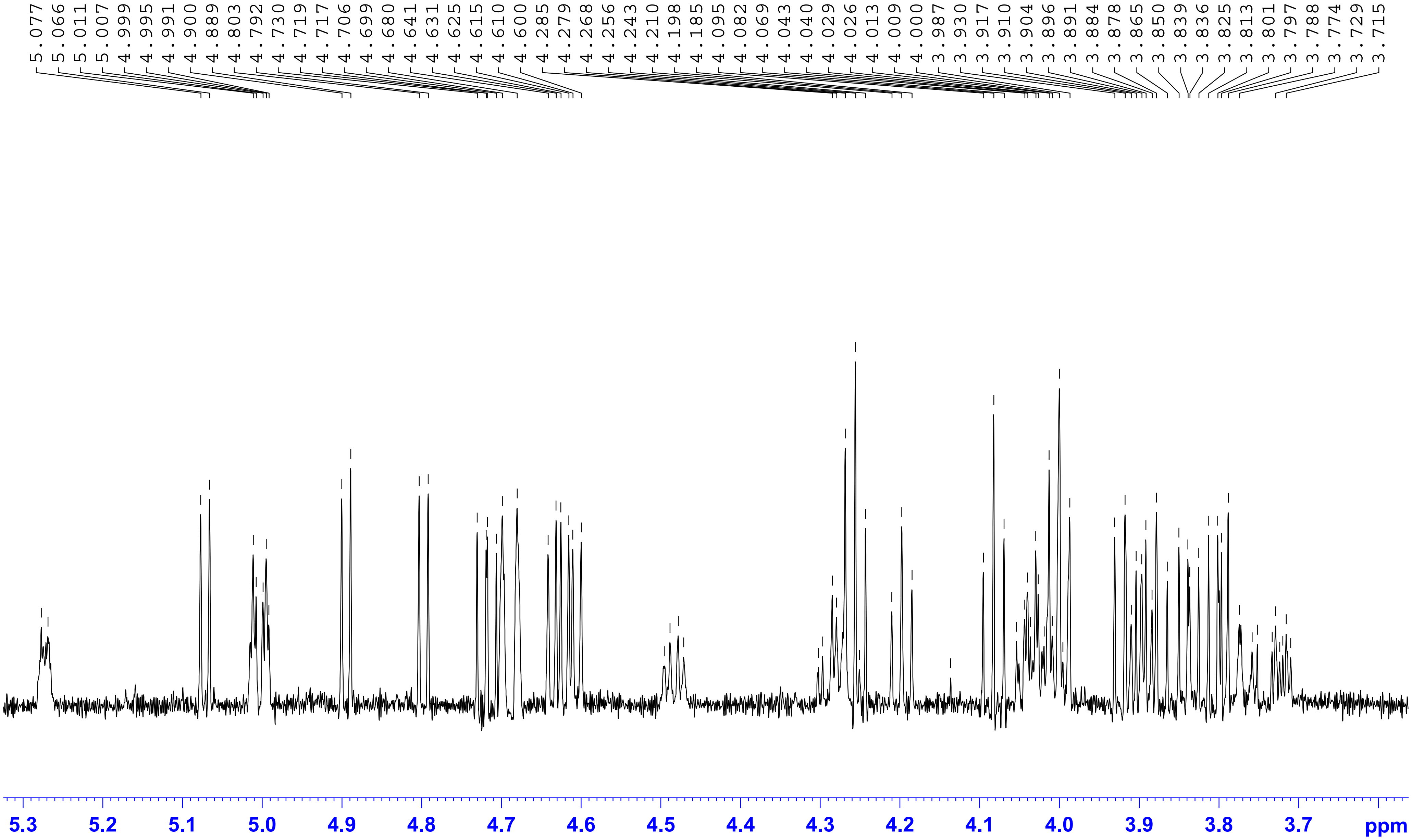

Figure 16. The ^{1}H NMR (700.13 MHz) spectrum of the carbohydrate moiety of **4** in C_5D_5N/D_2O (4/1).

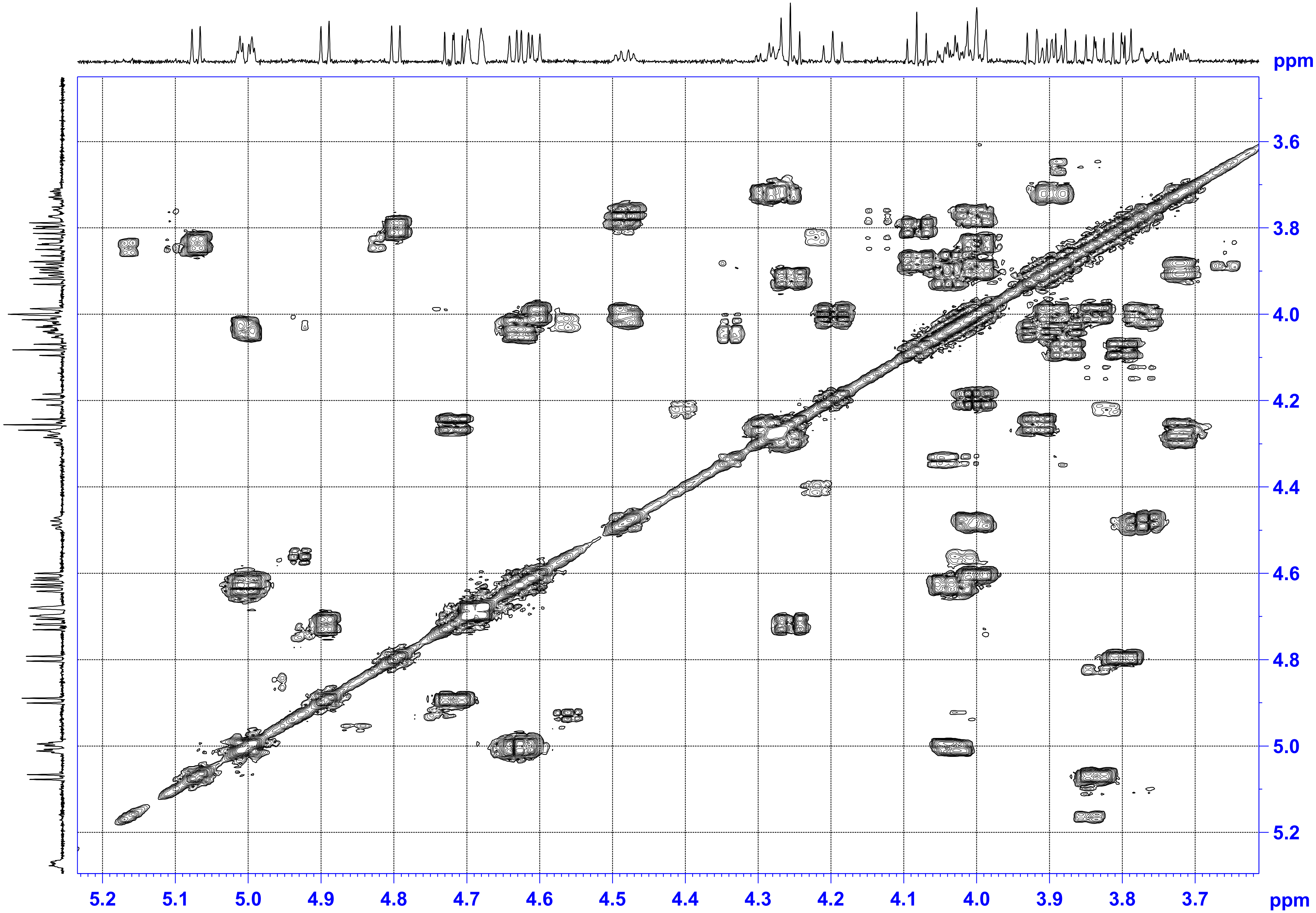

Figure 17. The COSY (700.13 MHz) spectrum of the carbohydrate moiety of **4** in C_5D_5N/D_2O (4/1).

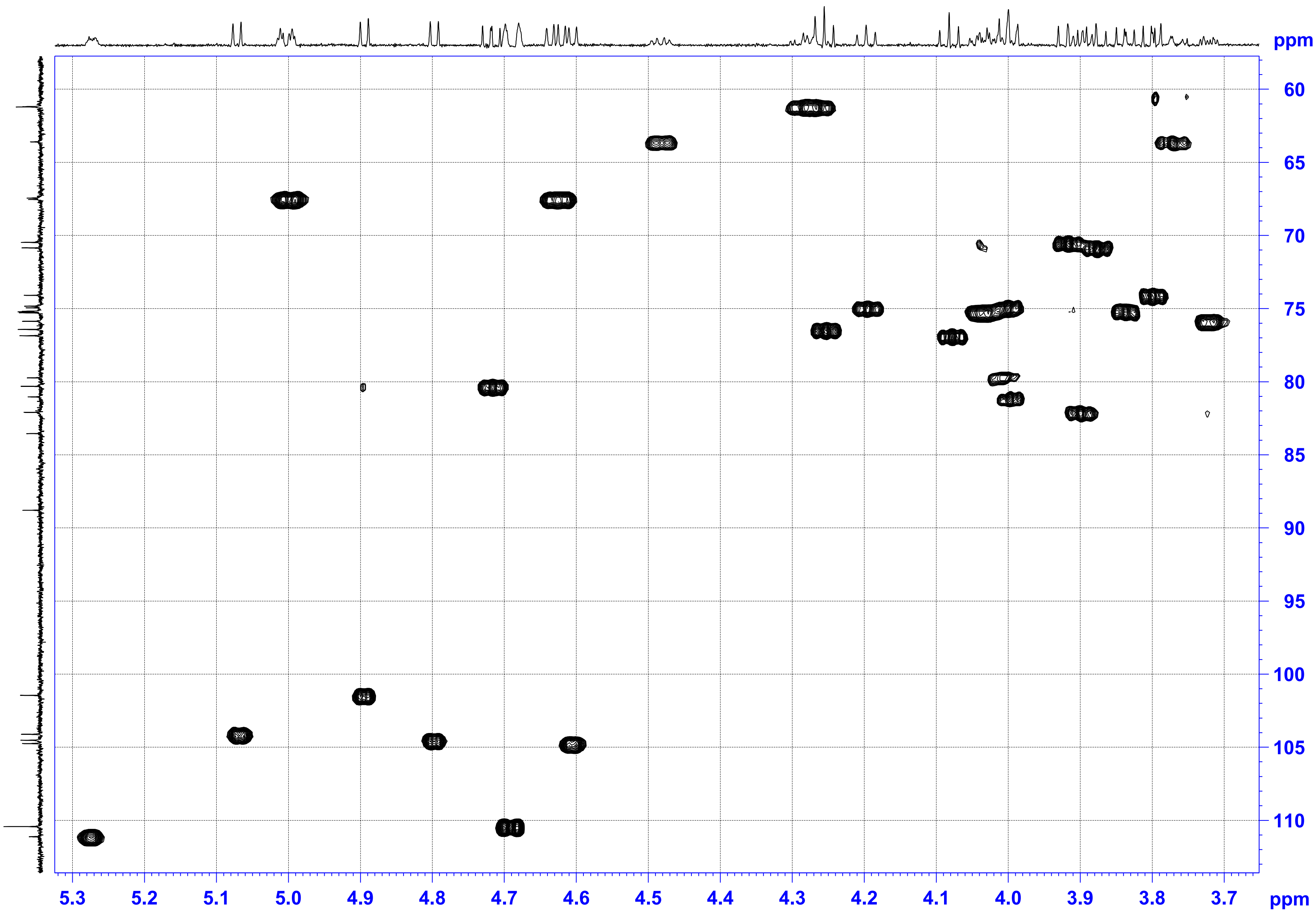

Figure 18. The HSQC (700.13 MHz) spectrum of the carbohydrate moiety of **4** in C_5D_5N/D_2O (4/1).

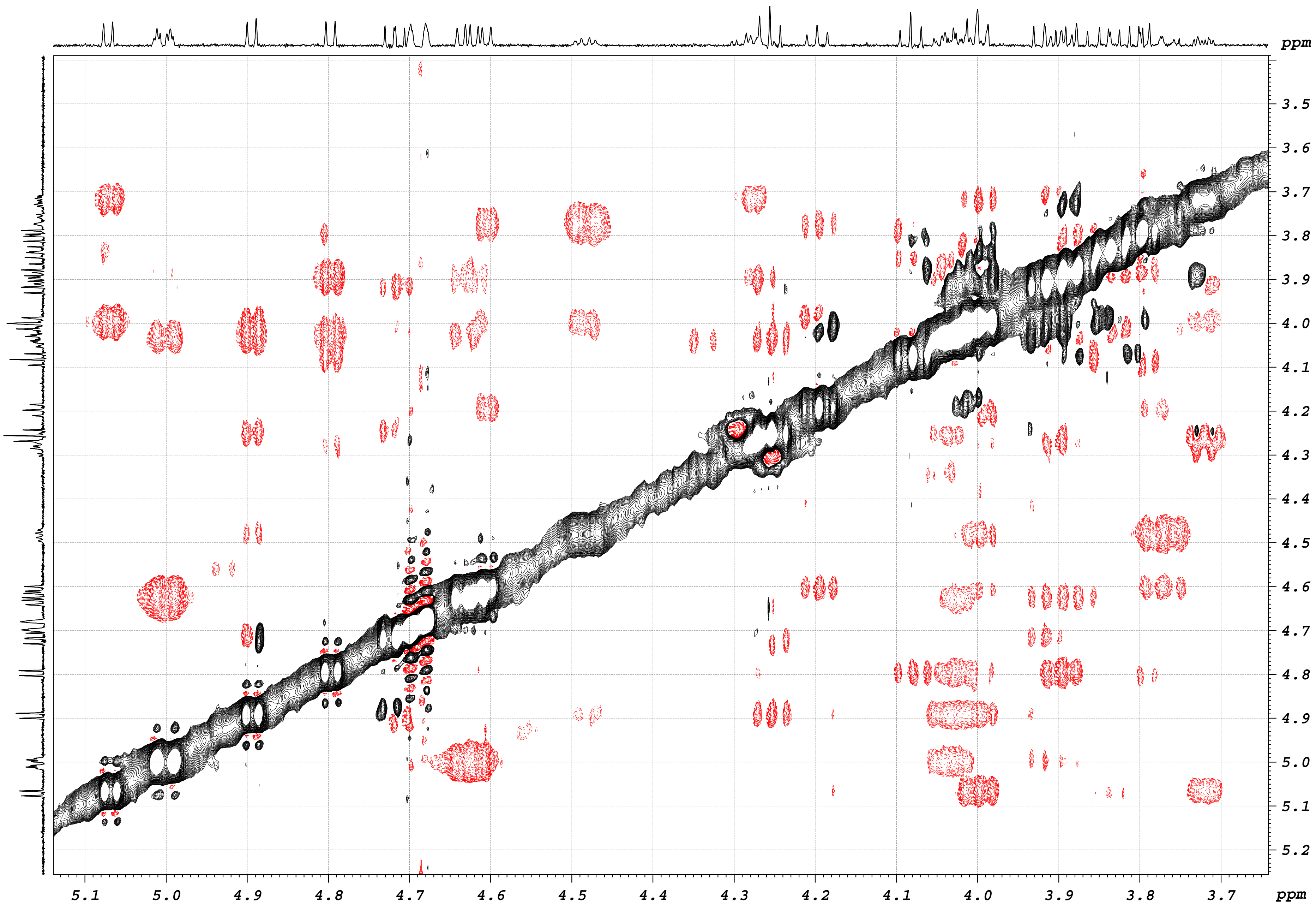

Figure 19. The ROESY (500.13 MHz) spectrum of the carbohydrate moiety of **4** in C_5D_5N/D_2O (4/1).

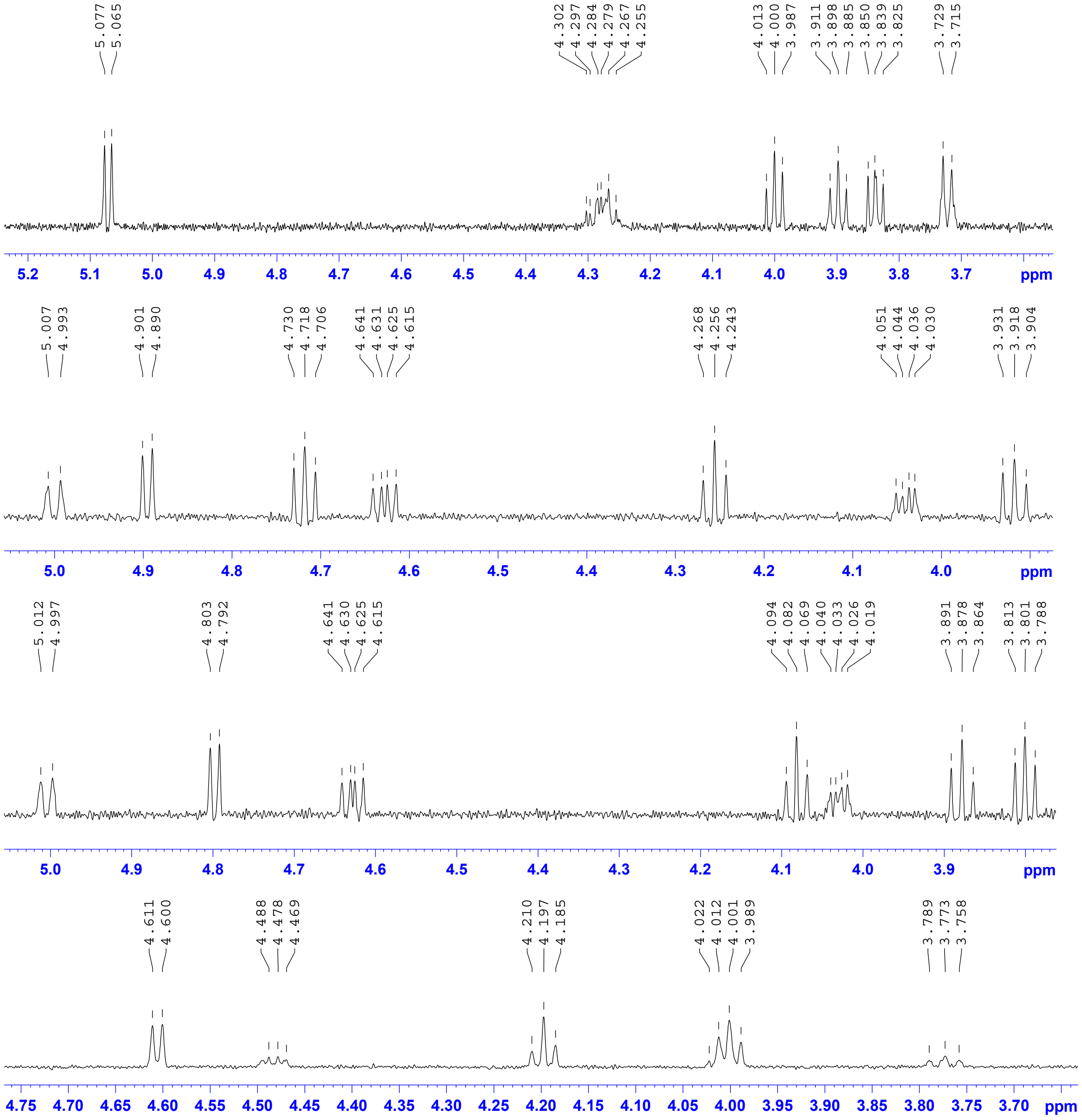

Figure 20. 1D TOCSY (700.13 MHz) spectra of the monosaccharide residues in **4** in C_5D_5N/D_2O (4/1).

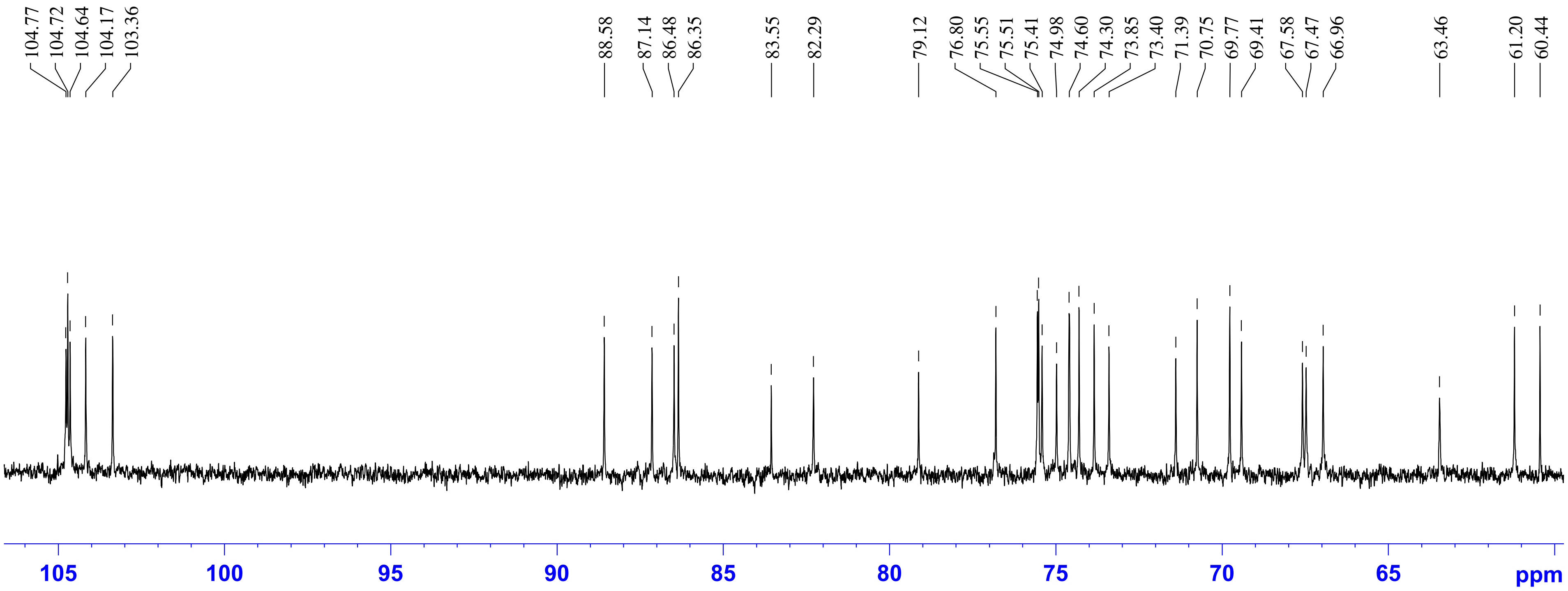

Figure 21. The ^{13}C NMR (176.04 MHz) spectrum of the carbohydrate moiety of **5** in C_5D_5N/D_2O (4/1).

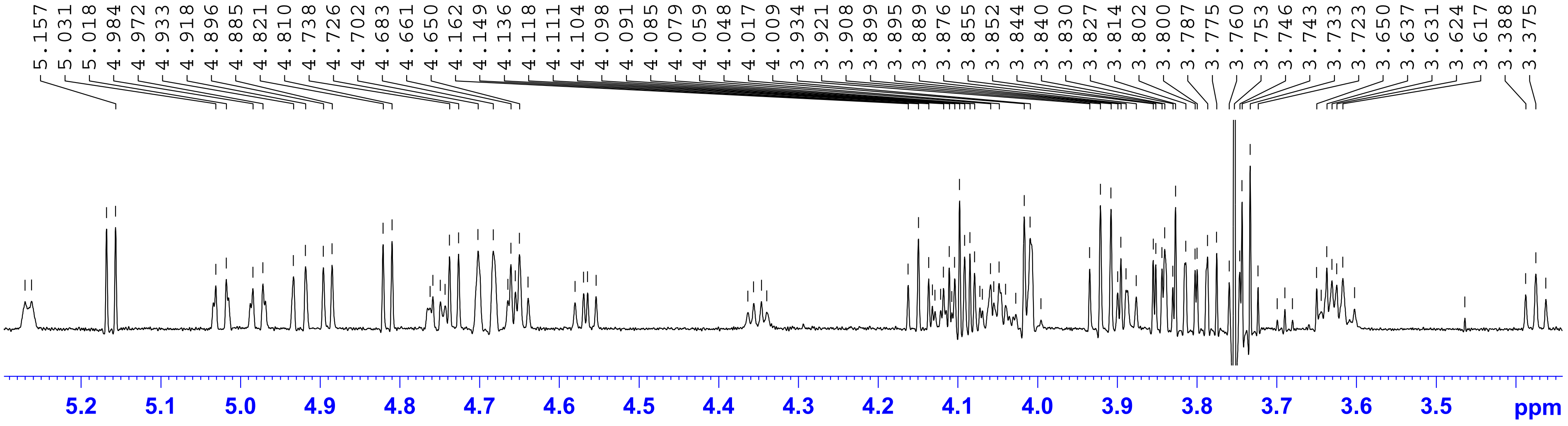

Figure 22. The ^{1}H NMR (700.13 MHz) spectrum of the carbohydrate moiety of **5** in C_5D_5N/D_2O (4/1).

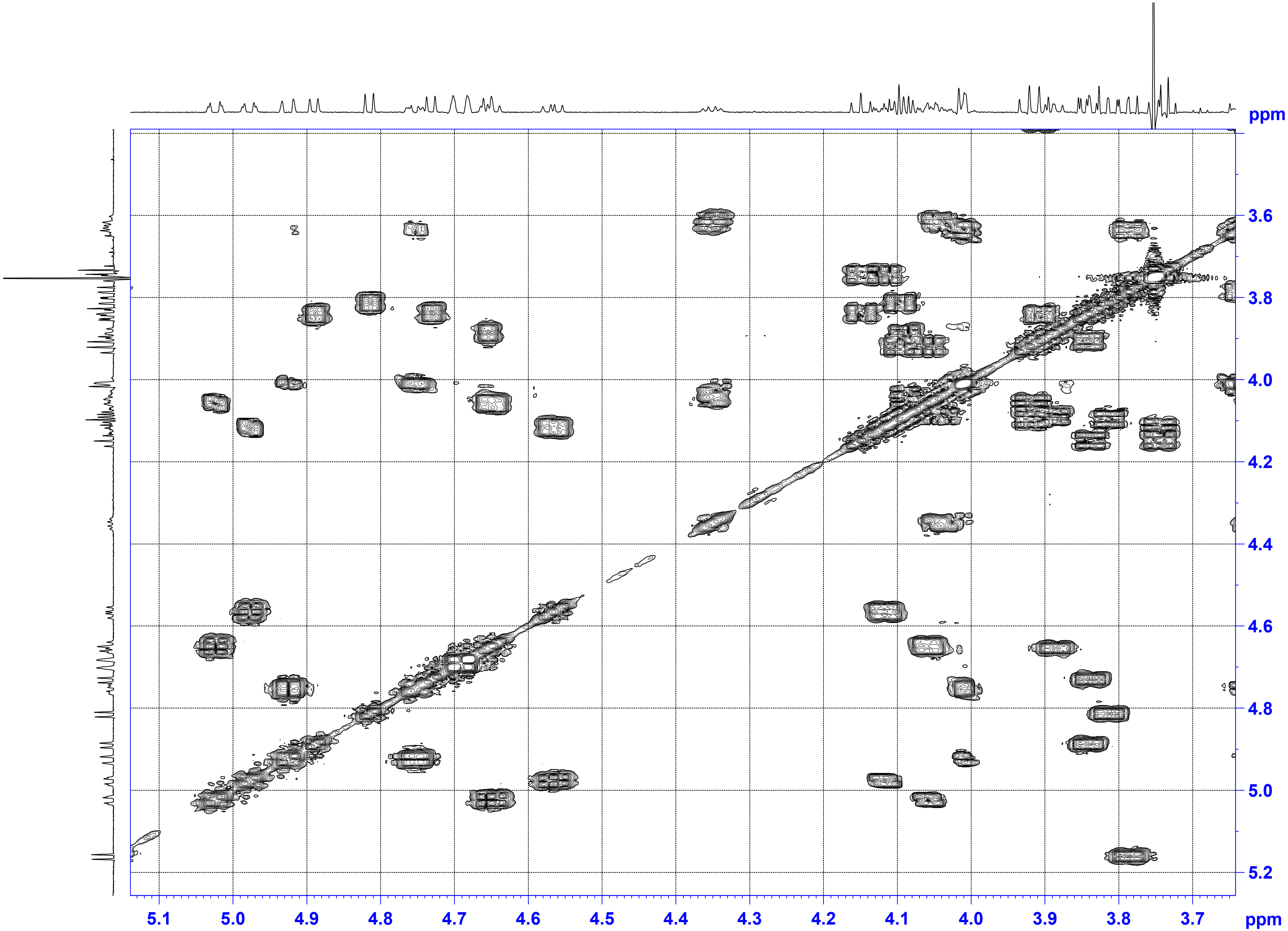

Figure 23. The COSY (700.13 MHz) spectrum of the carbohydrate moiety of **5** in C_5D_5N/D_2O (4/1).

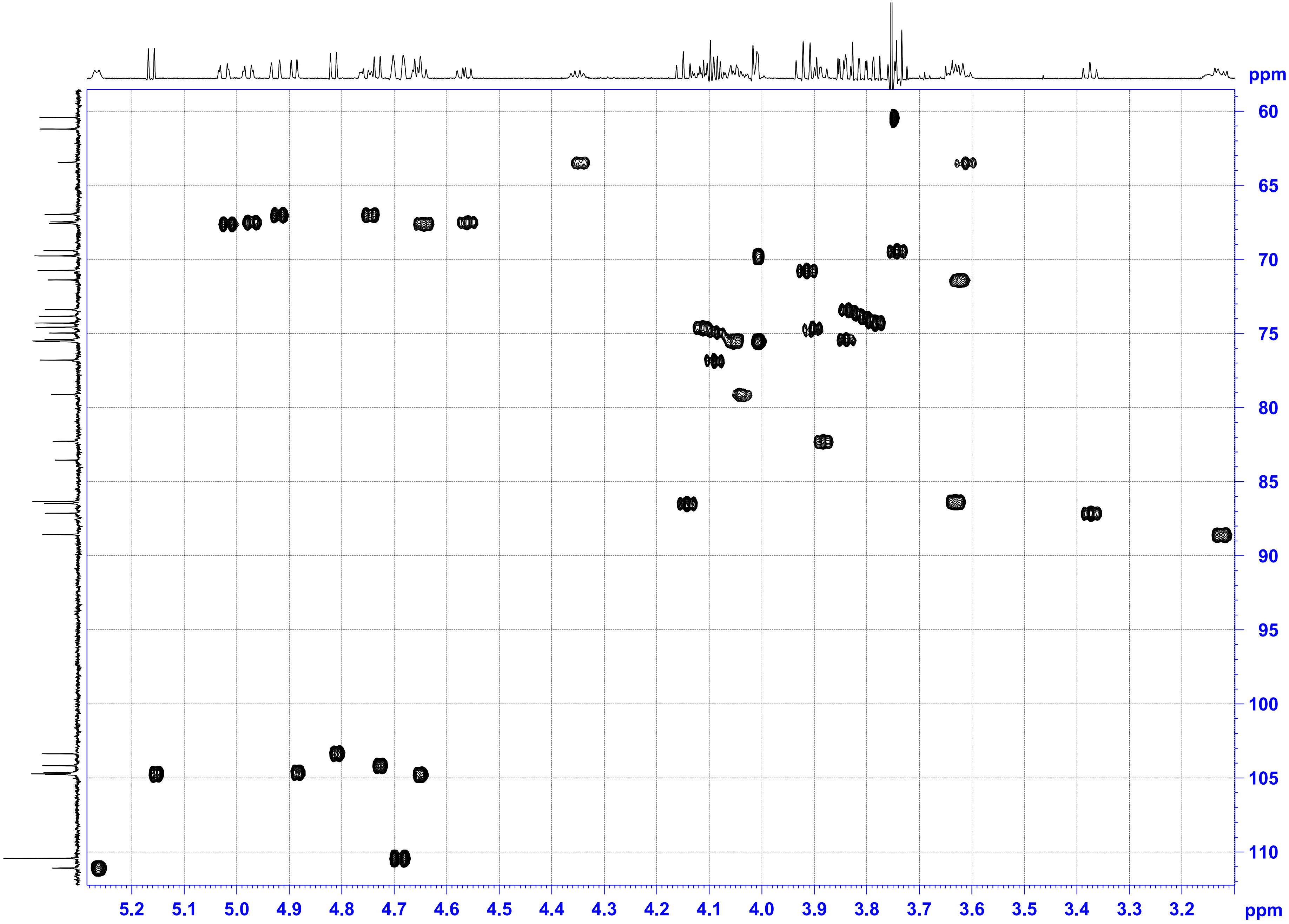

Figure 24. The HSQC (700.13 MHz) spectrum of the carbohydrate moiety of **5** in C_5D_5N/D_2O (4/1).

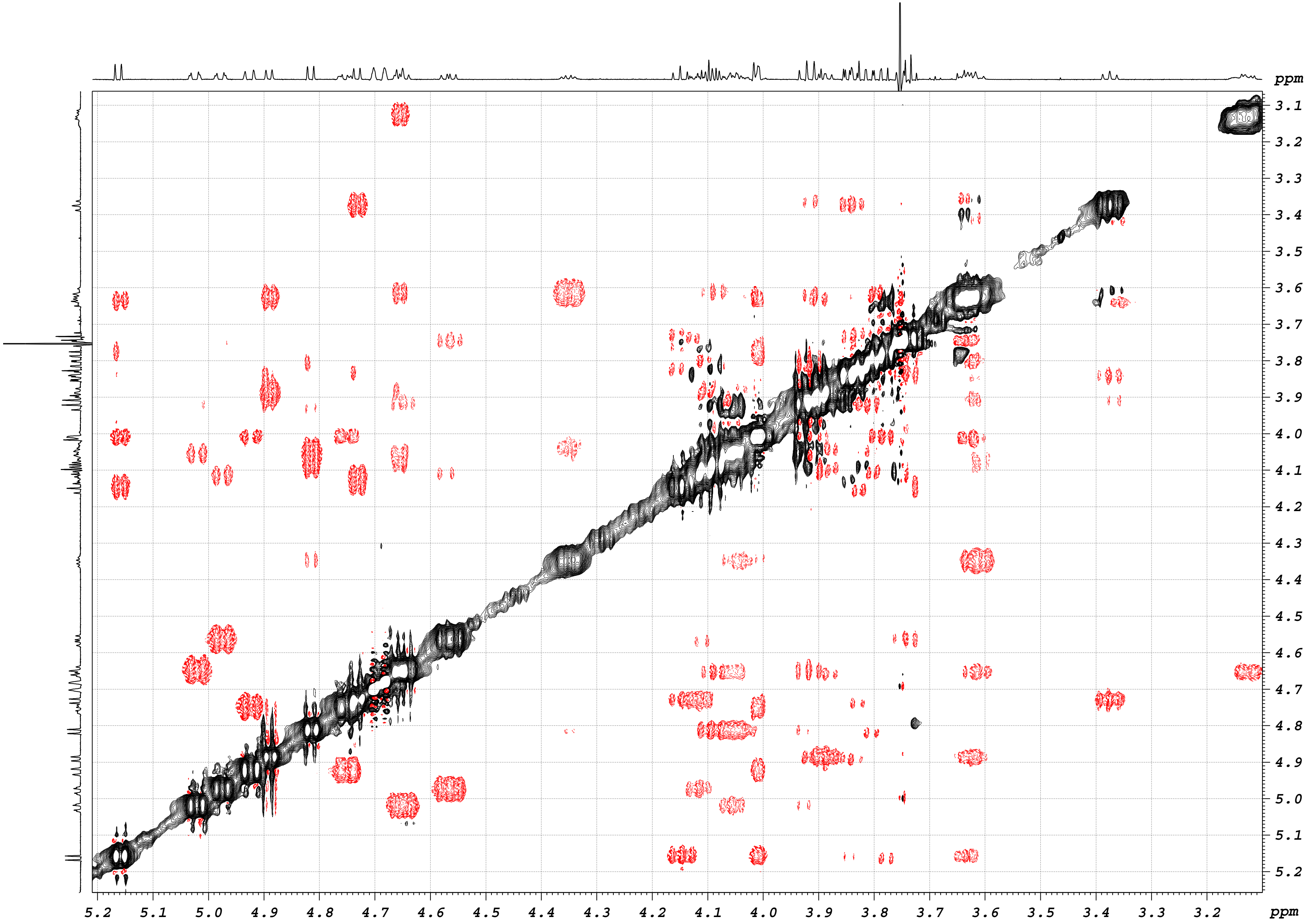

Figure 25. The ROESY (500.13 MHz) spectrum of the carbohydrate moiety of **5** in C_5D_5N/D_2O (4/1).

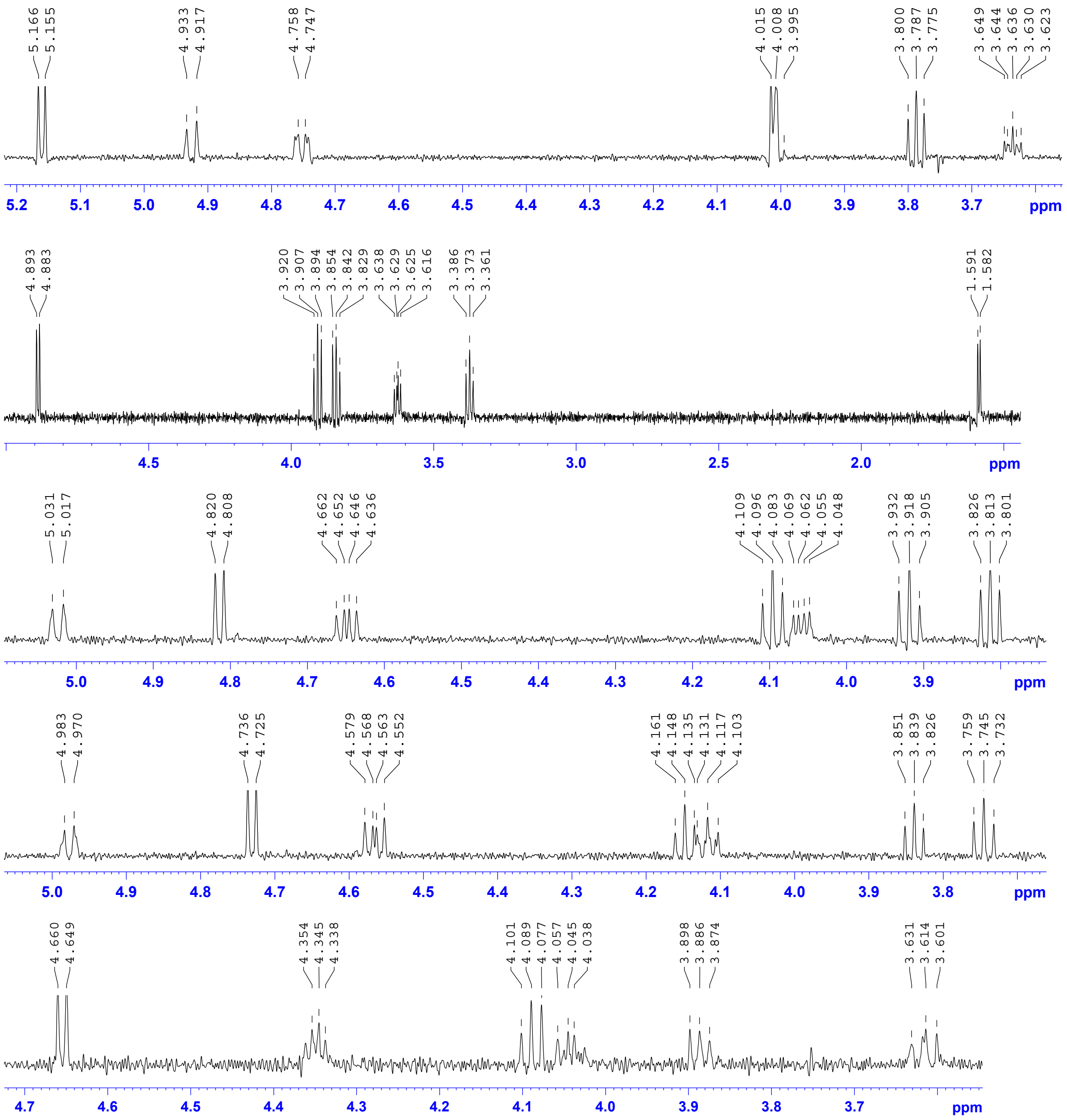

Figure 26. 1D TOCSY (700.13 MHz) spectra of the monosaccharide residues in **5** in C_5D_5N/D_2O (4/1).

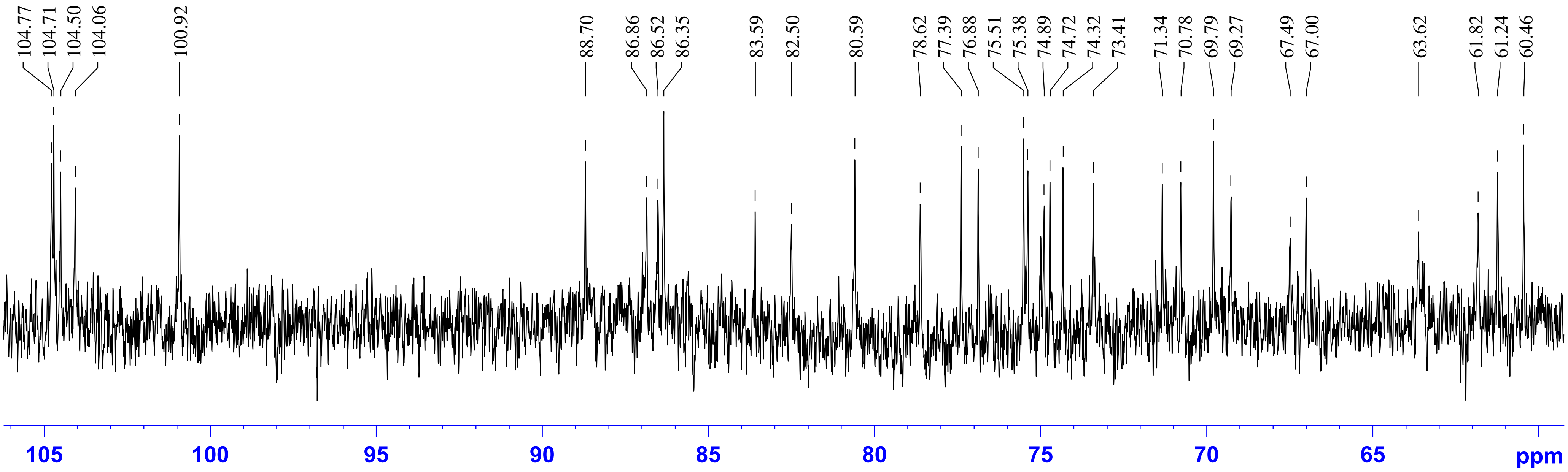

Figure 27. The ^{13}C NMR (176.04 MHz) spectrum of the carbohydrate moiety of **6** in C_5D_5N/D_2O (4/1).

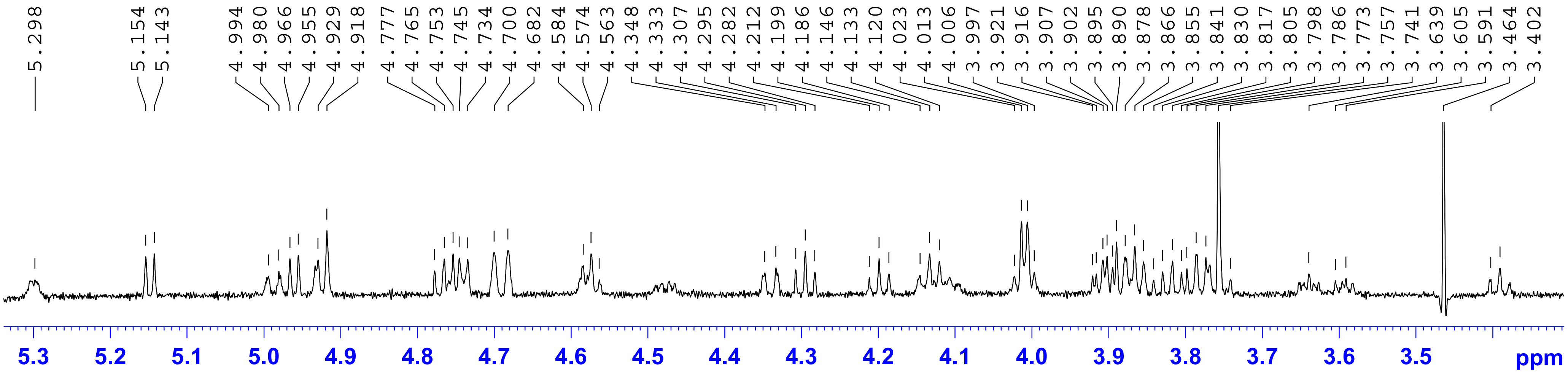

Figure 28. The ^{1}H NMR (700.13 MHz) spectrum of the carbohydrate moiety of **6** in C_5D_5N/D_2O (4/1).

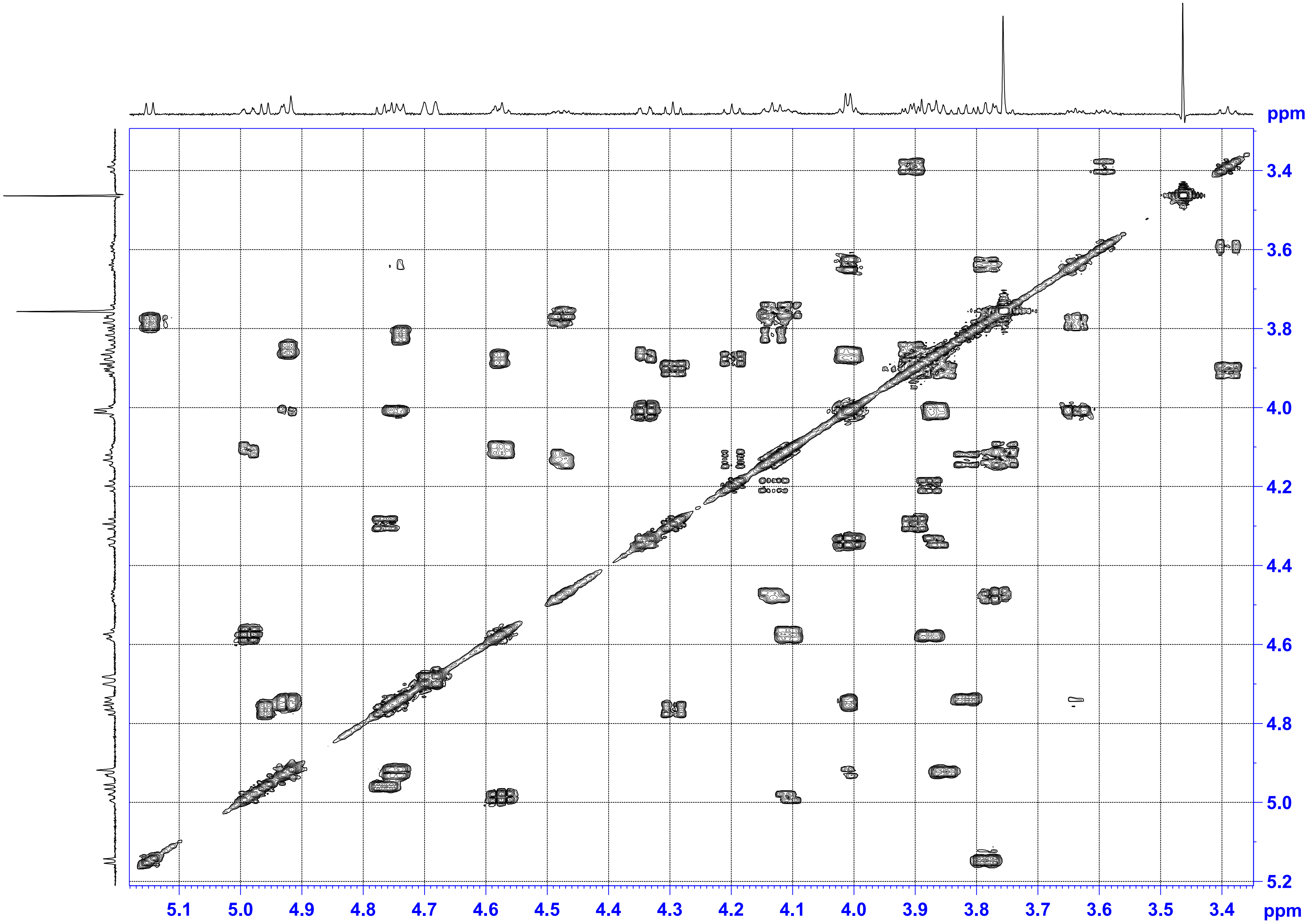

Figure 29. The COSY (700.13 MHz) spectrum of the carbohydrate moiety of **6** in C_5D_5N/D_2O (4/1).

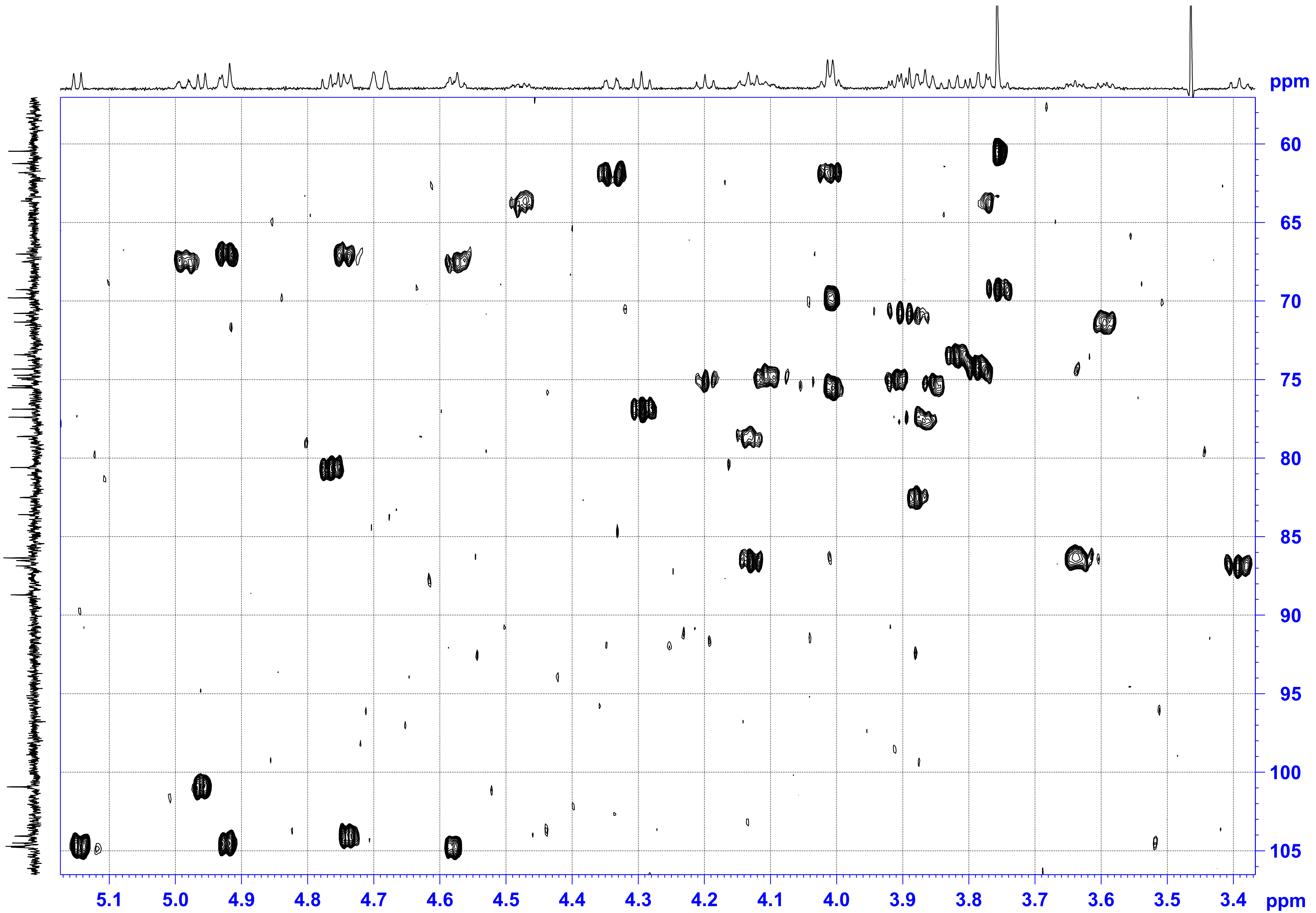

Figure 30. The HSQC (700.13 MHz) spectrum of the carbohydrate moiety of **6** in C_5D_5N/D_2O (4/1).

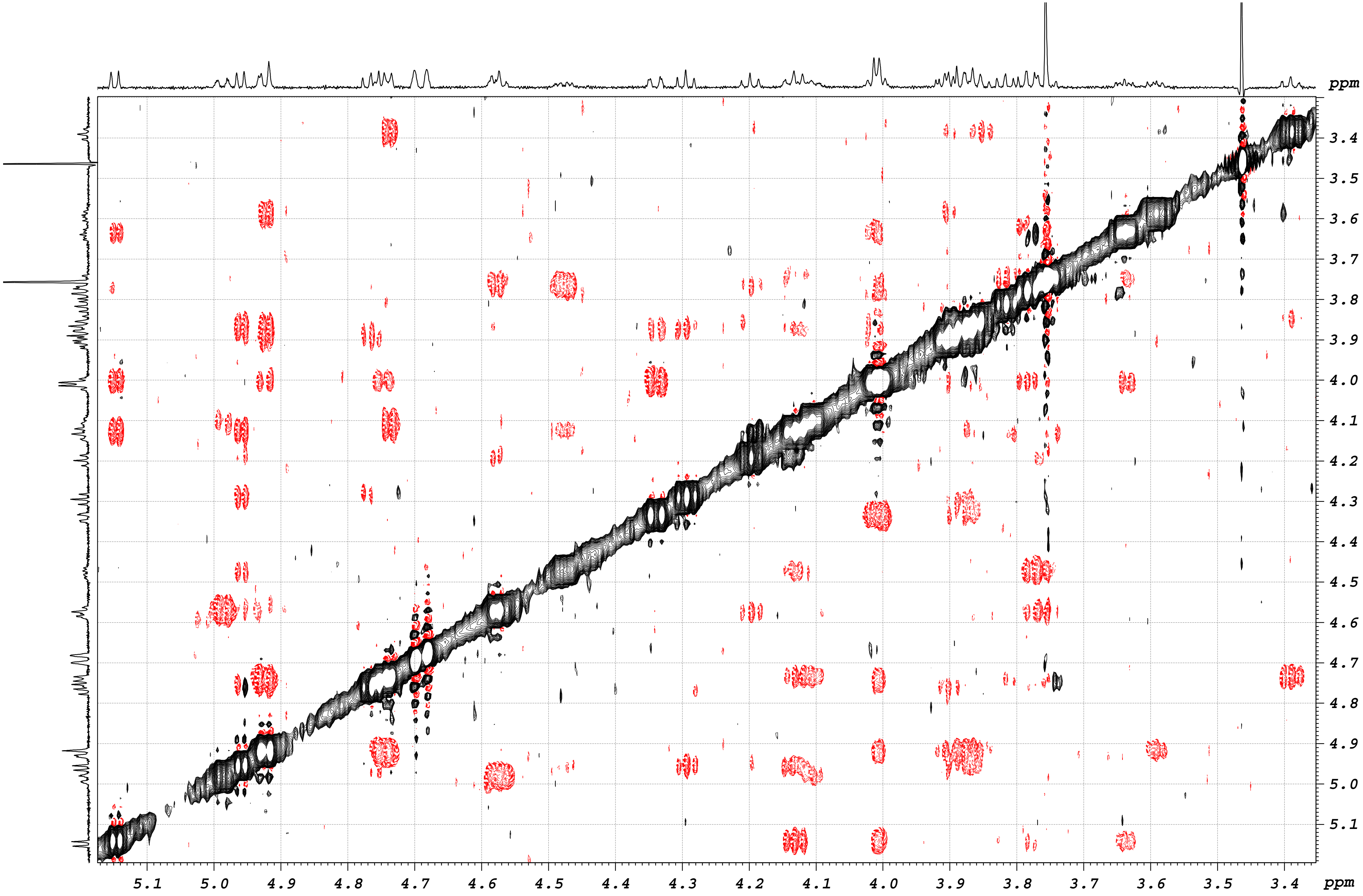

Figure 31. The ROESY (500.13 MHz) spectrum of the carbohydrate moiety of **6** in C_5D_5N/D_2O (4/1).

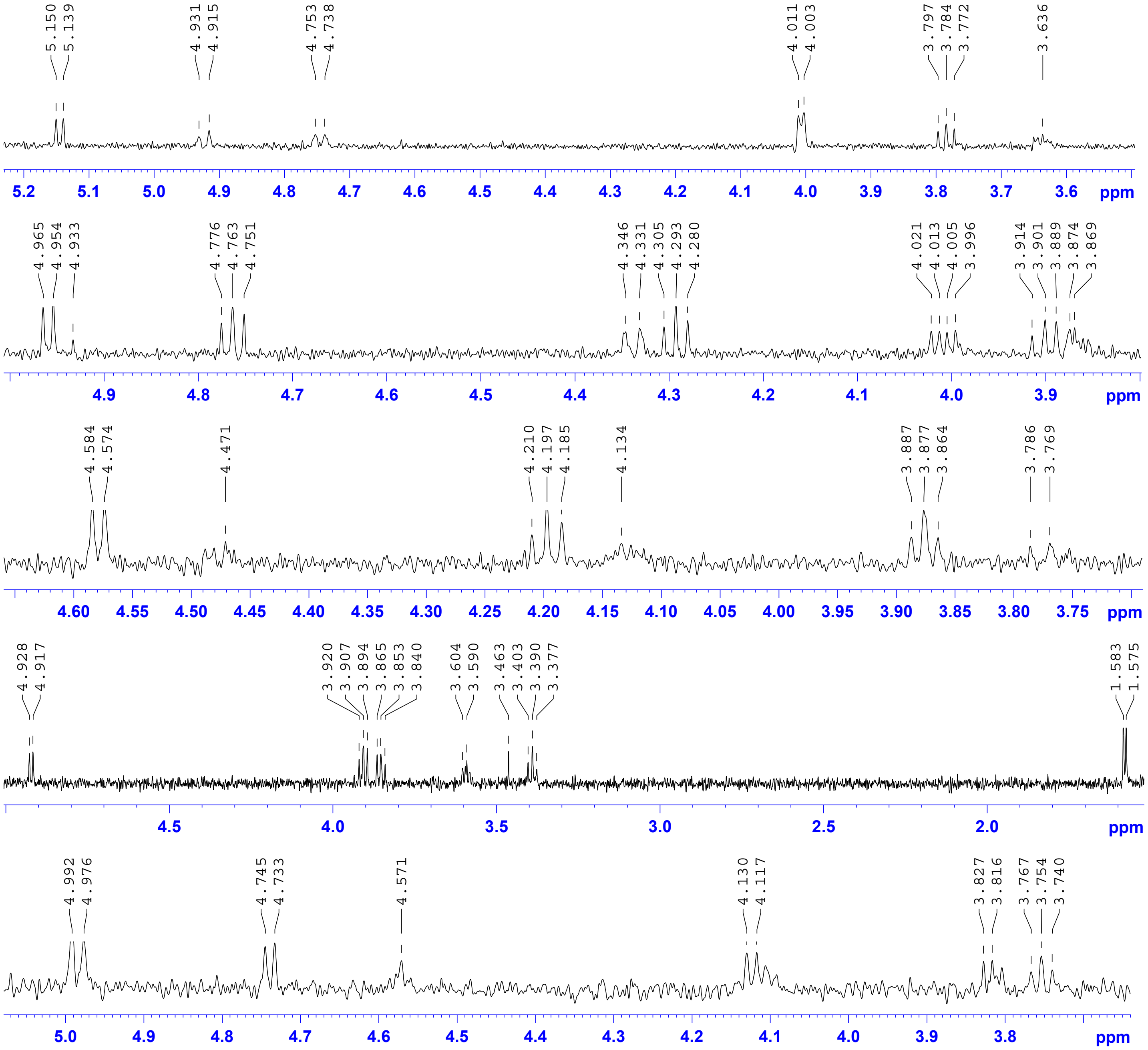

Figure 32. 1D TOCSY (700.13 MHz) spectra of the monosaccharide residues in **6** in C_5D_5N/D_2O (4/1).

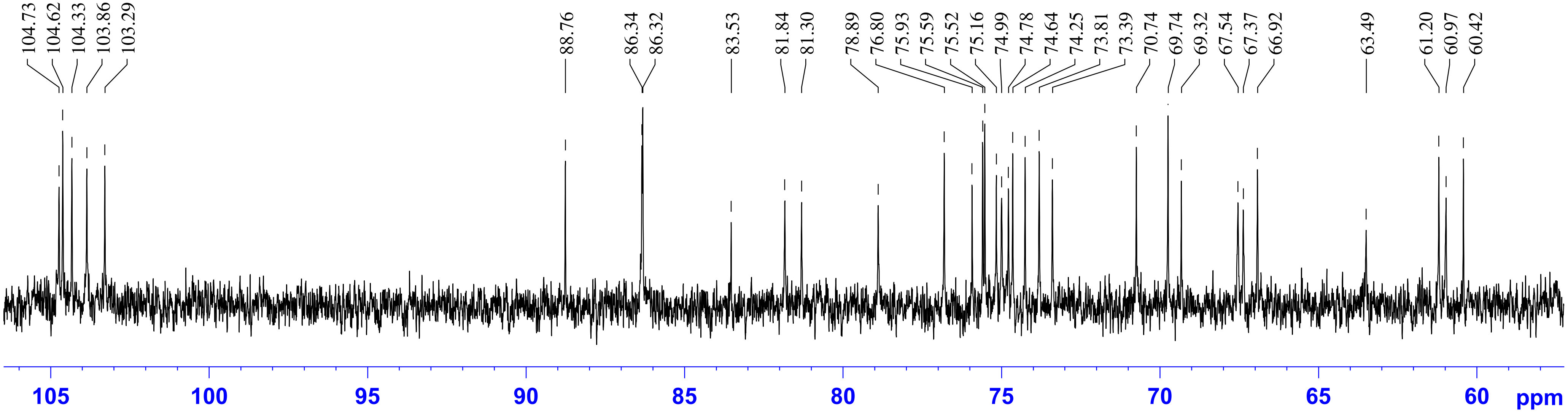

Figure 33. The ^{13}C NMR (176.04 MHz) spectrum of the carbohydrate moiety of **7** in C_5D_5N/D_2O (4/1).

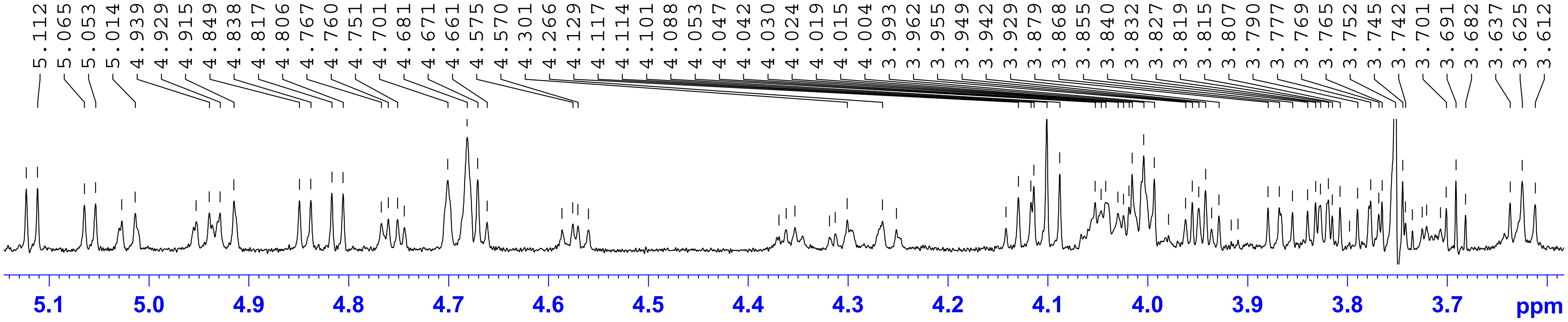

Figure 34. The ^{1}H NMR (700.13 MHz) spectrum of the carbohydrate moiety of **7** in C_5D_5N/D_2O (4/1).

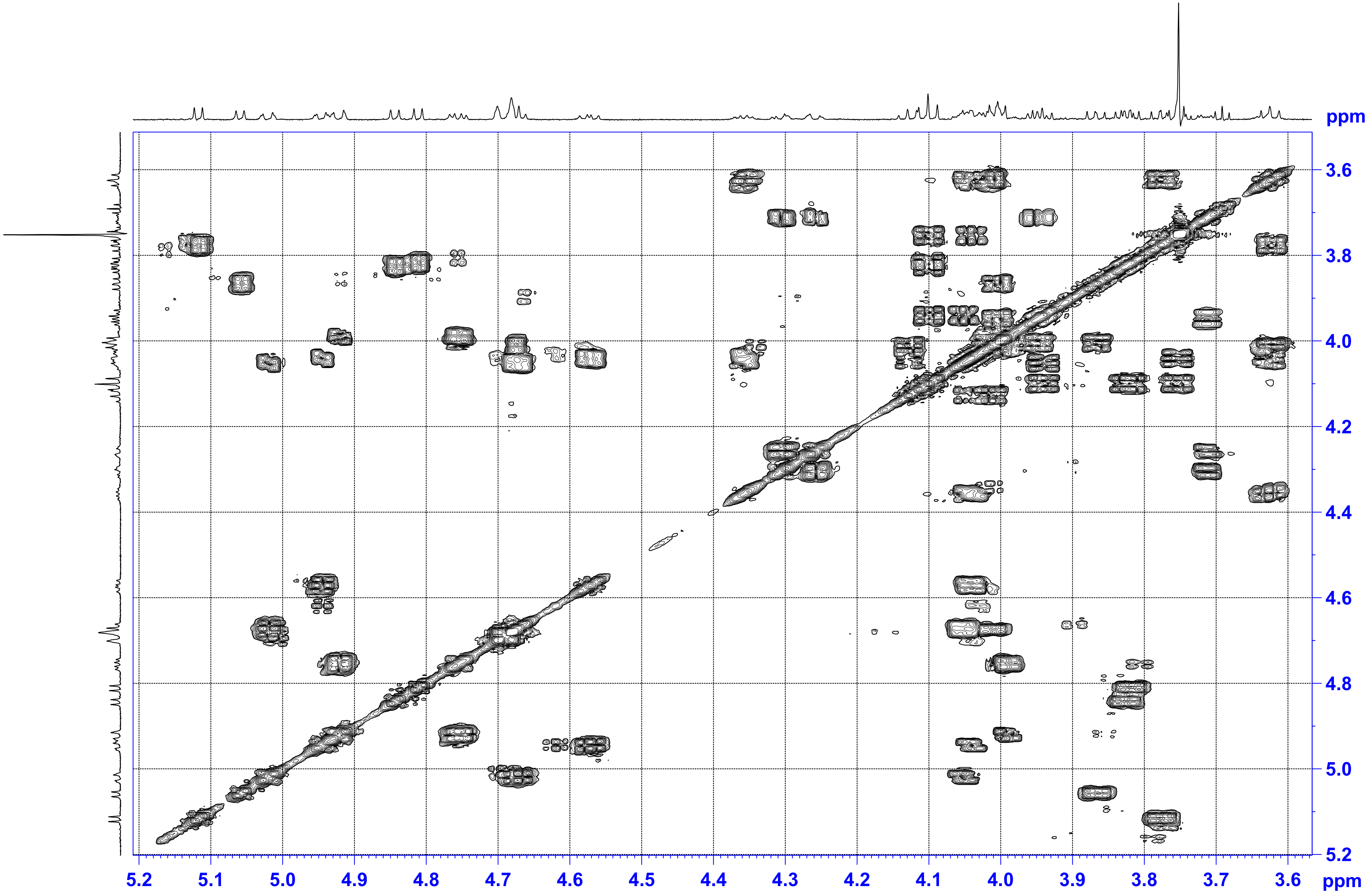

Figure 35. The COSY (700.13 MHz) spectrum of the carbohydrate moiety of **7** in C_5D_5N/D_2O (4/1).

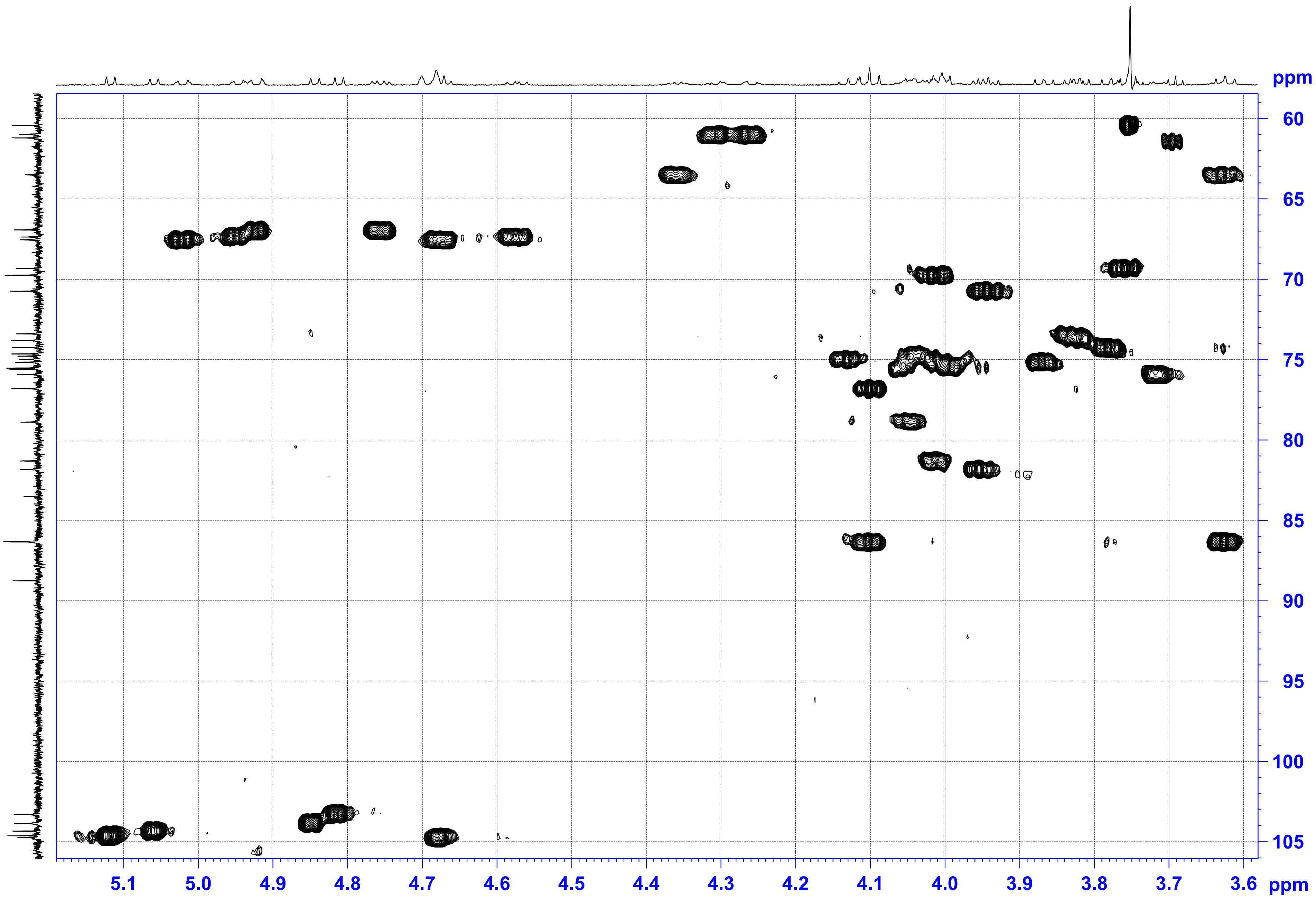

Figure 36. The HSQC (700.13 MHz) spectrum of the carbohydrate moiety of **7** in C_5D_5N/D_2O (4/1).

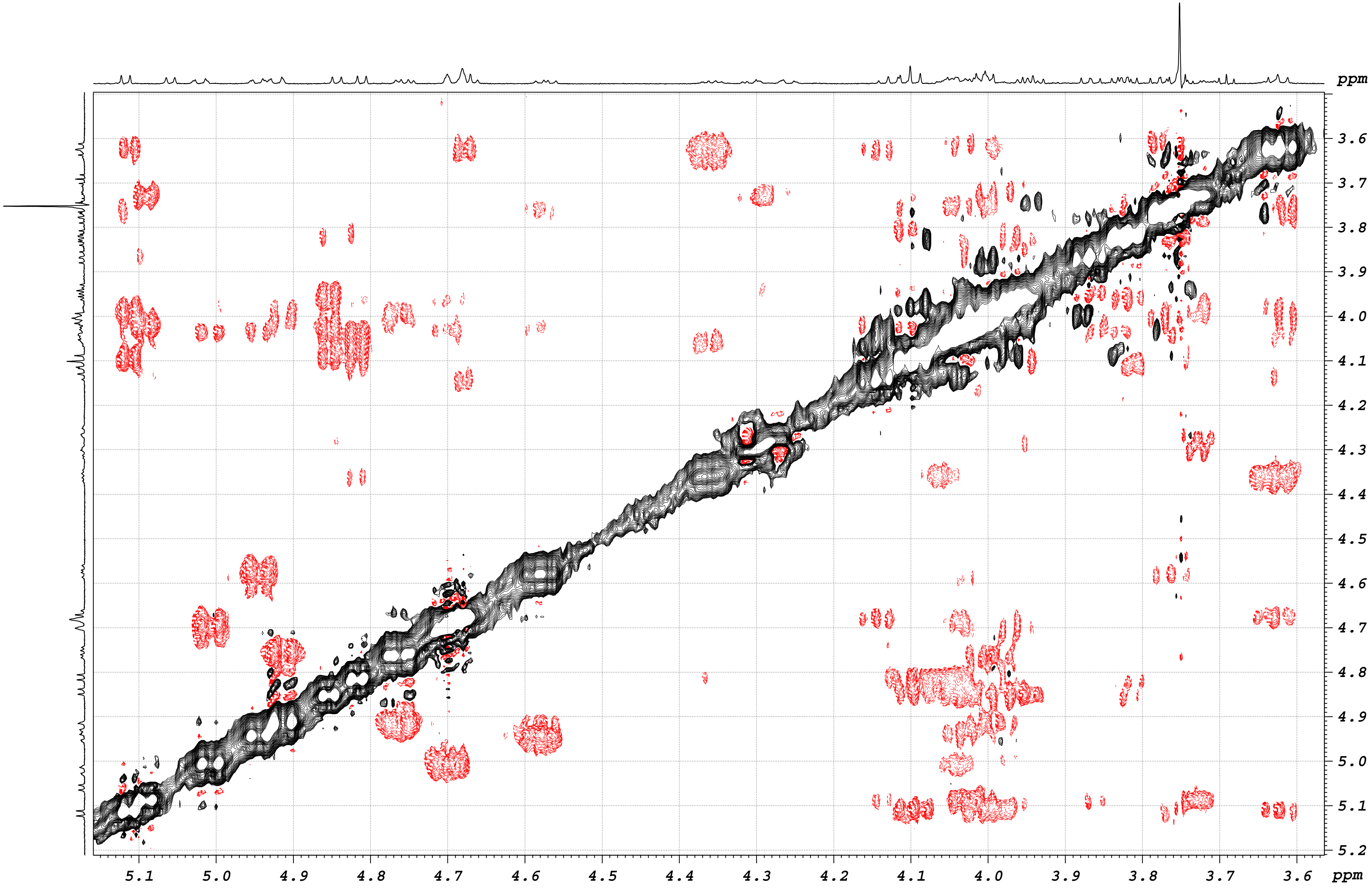

Figure 37. The ROESY (500.13 MHz) spectrum of the carbohydrate moiety of **7** in C_5D_5N/D_2O (4/1).

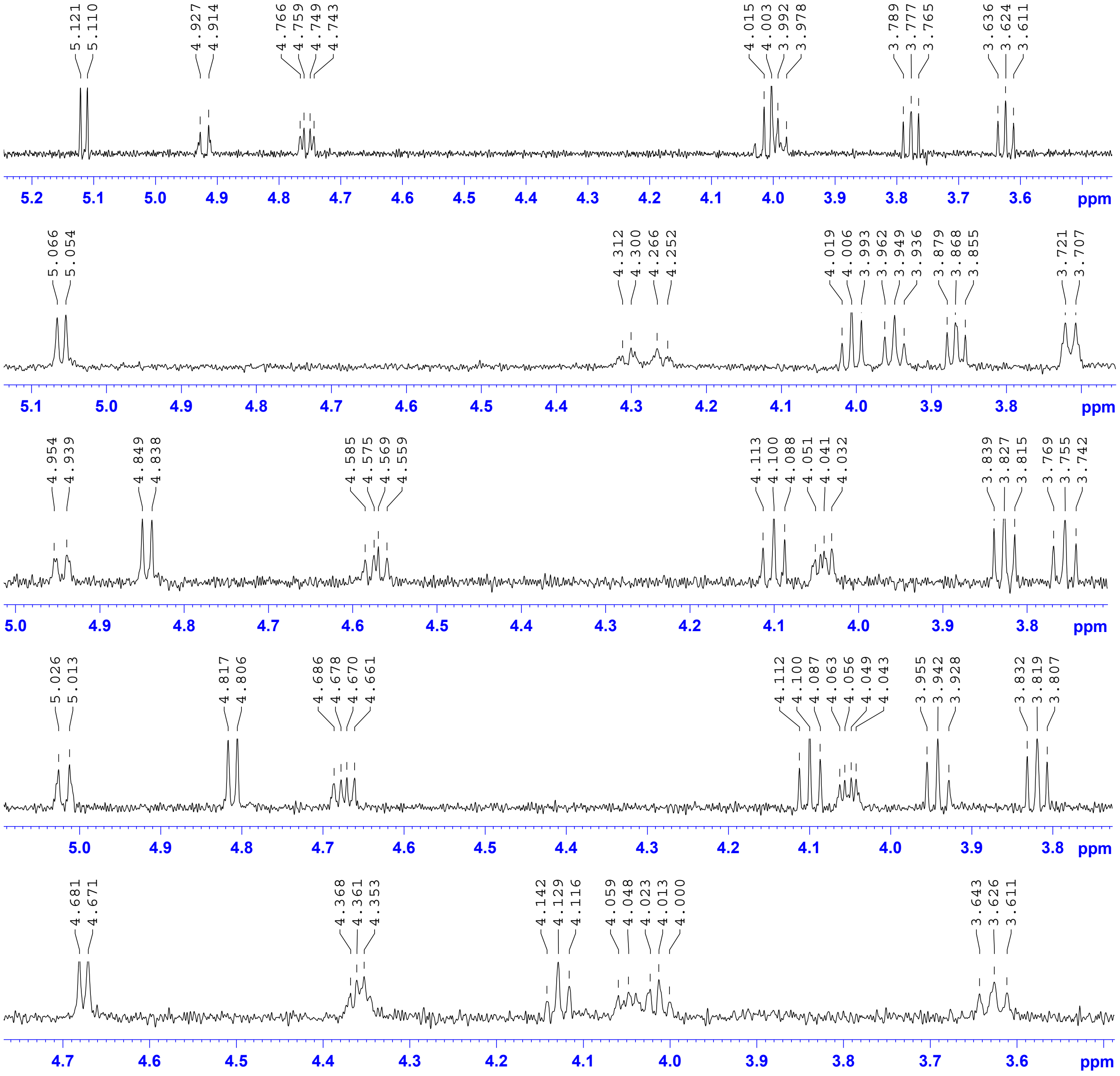

Figure 38. 1D TOCSY (700.13 MHz) spectra of the monosaccharide residues in **7** in C_5D_5N/D_2O (4/1).

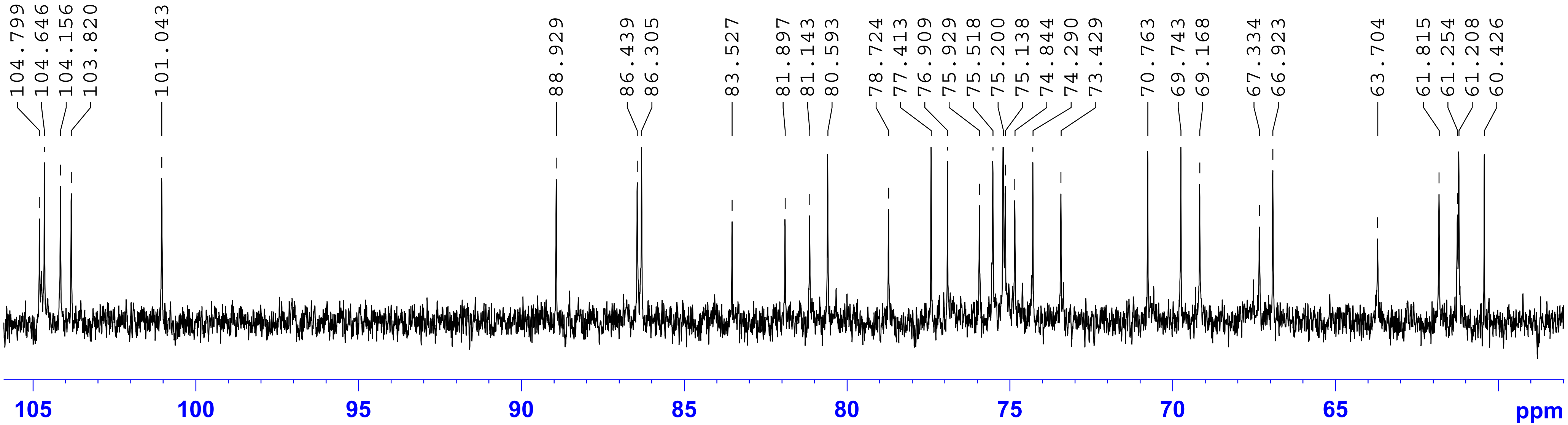

Figure 39. The ^{13}C NMR (176.04 MHz) spectrum of the carbohydrate moiety of **8** in C_5D_5N/D_2O (4/1).

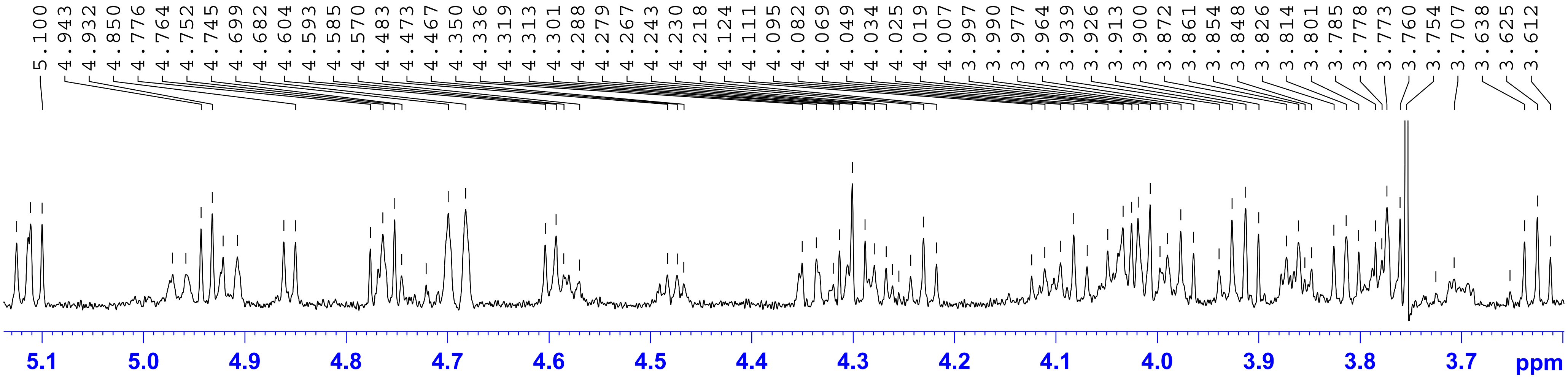

Figure 40. The ^{1}H NMR (700.13 MHz) spectrum of the carbohydrate moiety of **8** in C_5D_5N/D_2O (4/1).

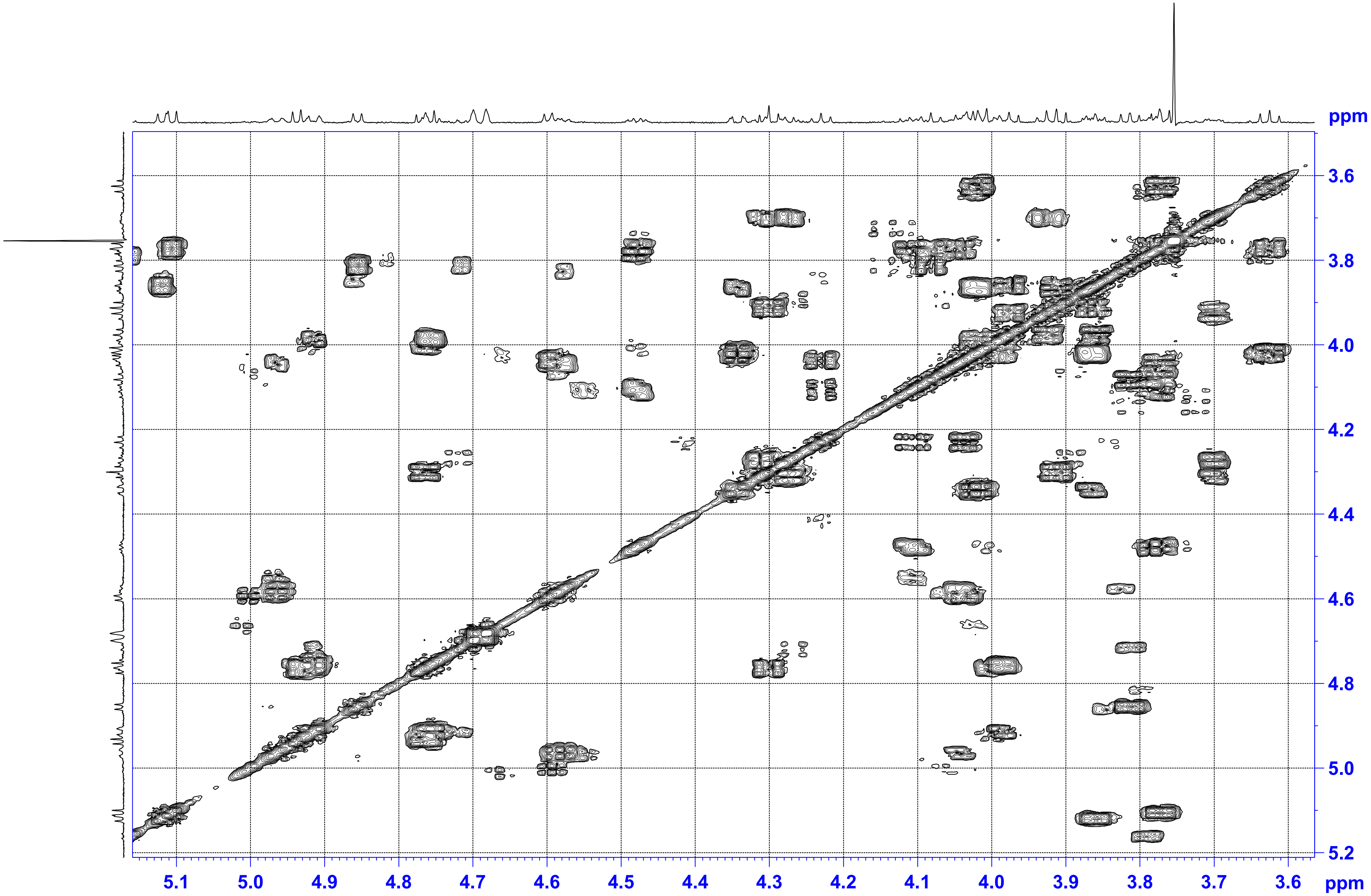

Figure 41. The COSY (700.13 MHz) spectrum of the carbohydrate moiety of **8** in C_5D_5N/D_2O (4/1).

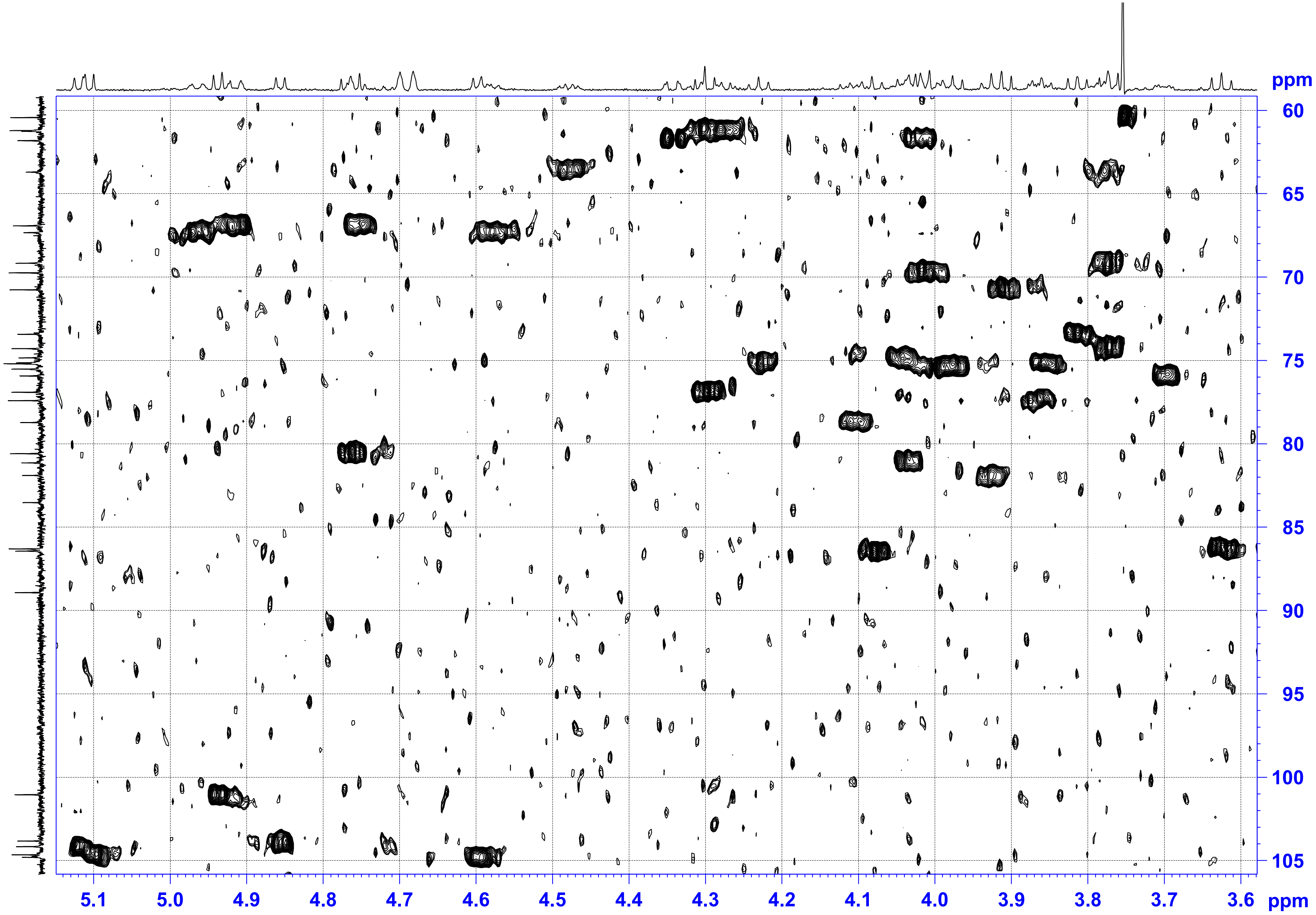

Figure 42. The HSQC (700.13 MHz) spectrum of the carbohydrate moiety of **8** in C_5D_5N/D_2O (4/1).

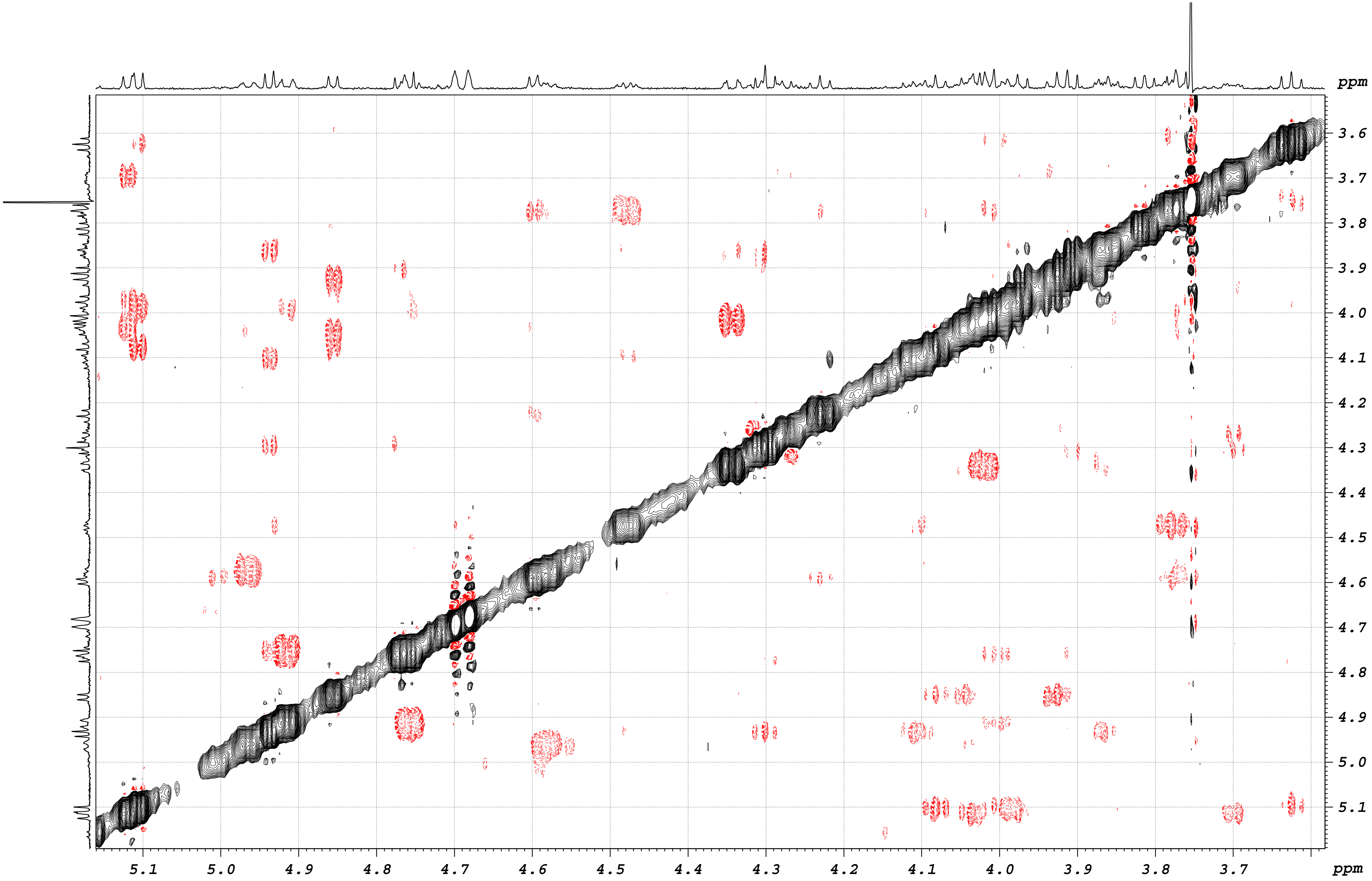

Figure 43. The ROESY (500.13 MHz) spectrum of the carbohydrate moiety of **8** in C_5D_5N/D_2O (4/1).

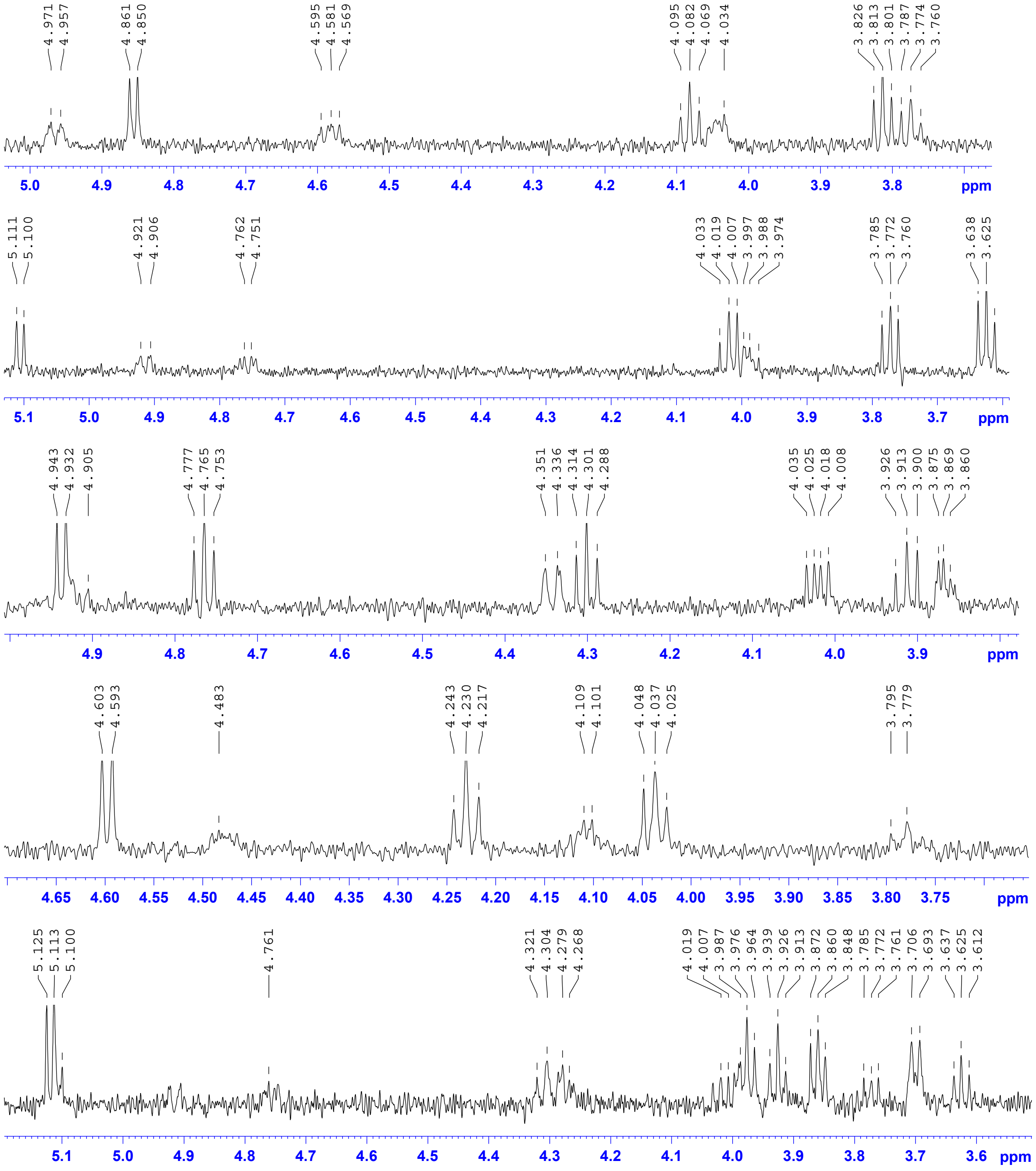

Figure 44. 1D TOCSY (700.13 MHz) spectra of the monosaccharide residues in **8** in C_5D_5N/D_2O (4/1).

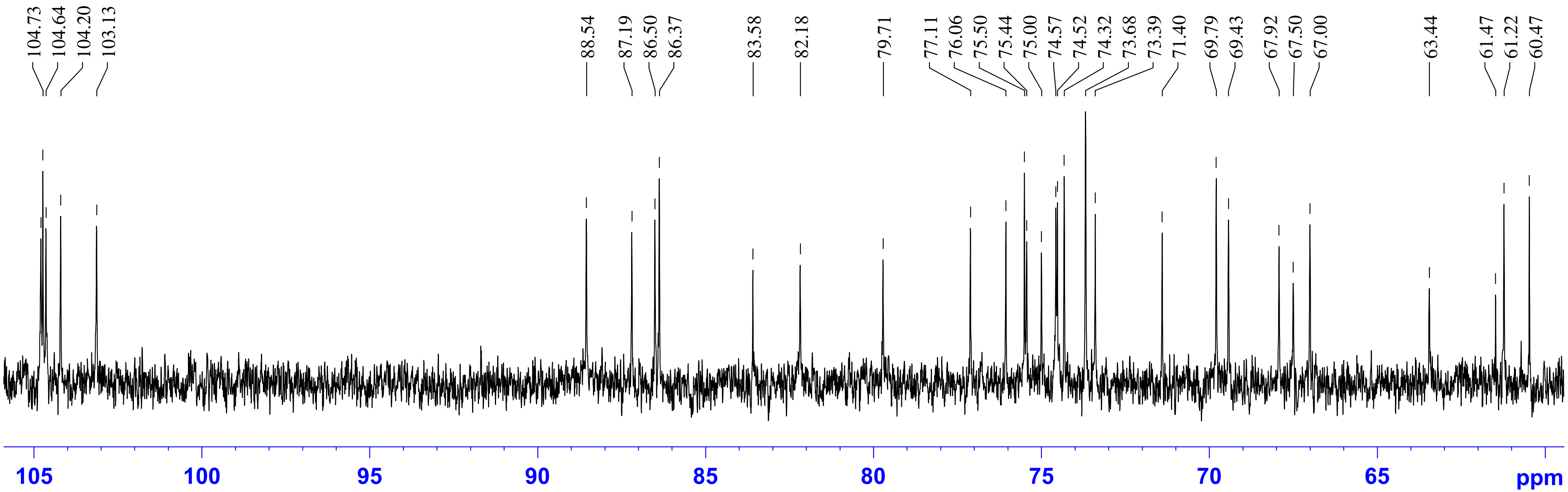

Figure 45. The ^{13}C NMR (176.04 MHz) spectrum of the carbohydrate moiety of **9** in C_5D_5N/D_2O (4/1).

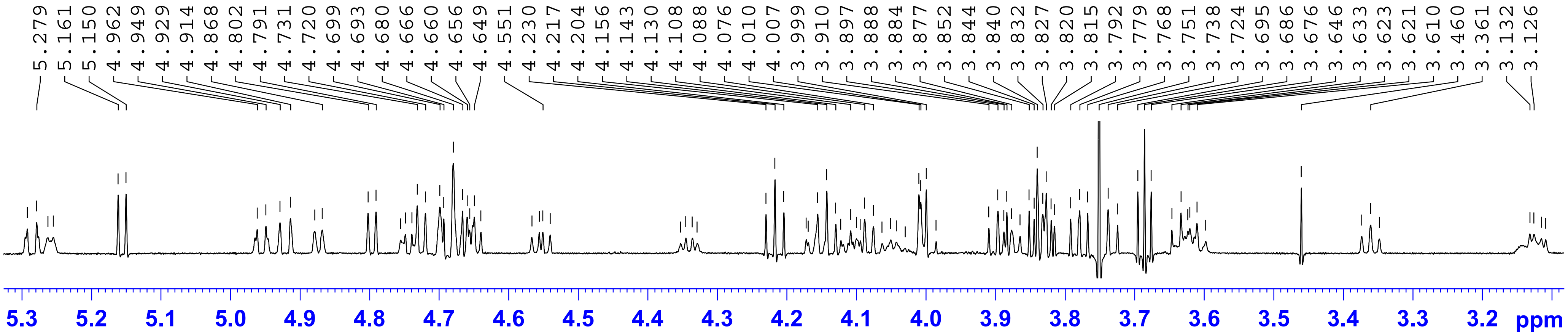

Figure 46. The ^{1}H NMR (700.13 MHz) spectrum of the carbohydrate moiety of **9** in C_5D_5N/D_2O (4/1).

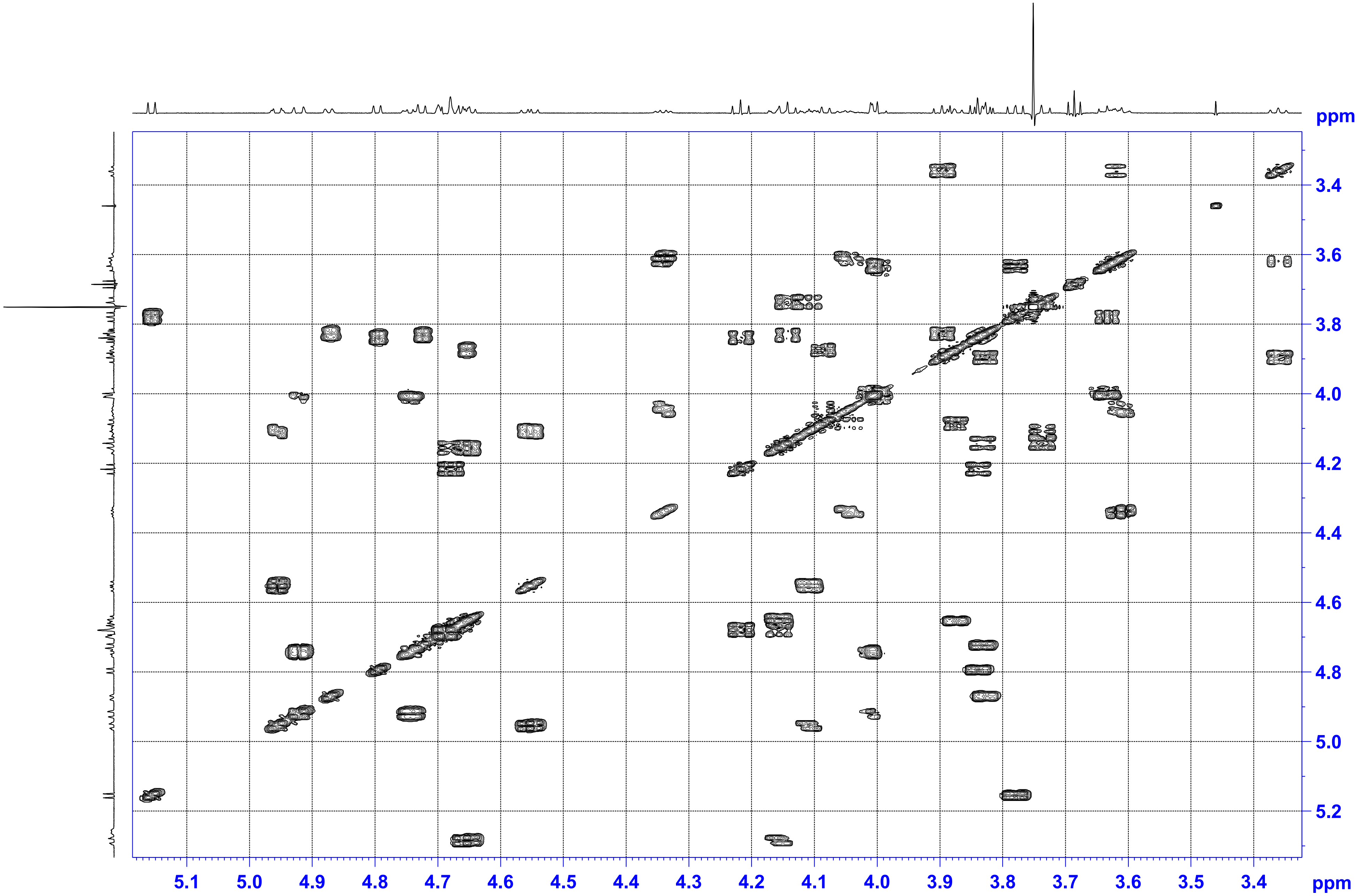

Figure 47. The COSY (700.13 MHz) spectrum of the carbohydrate moiety of **9** in C_5D_5N/D_2O (4/1).

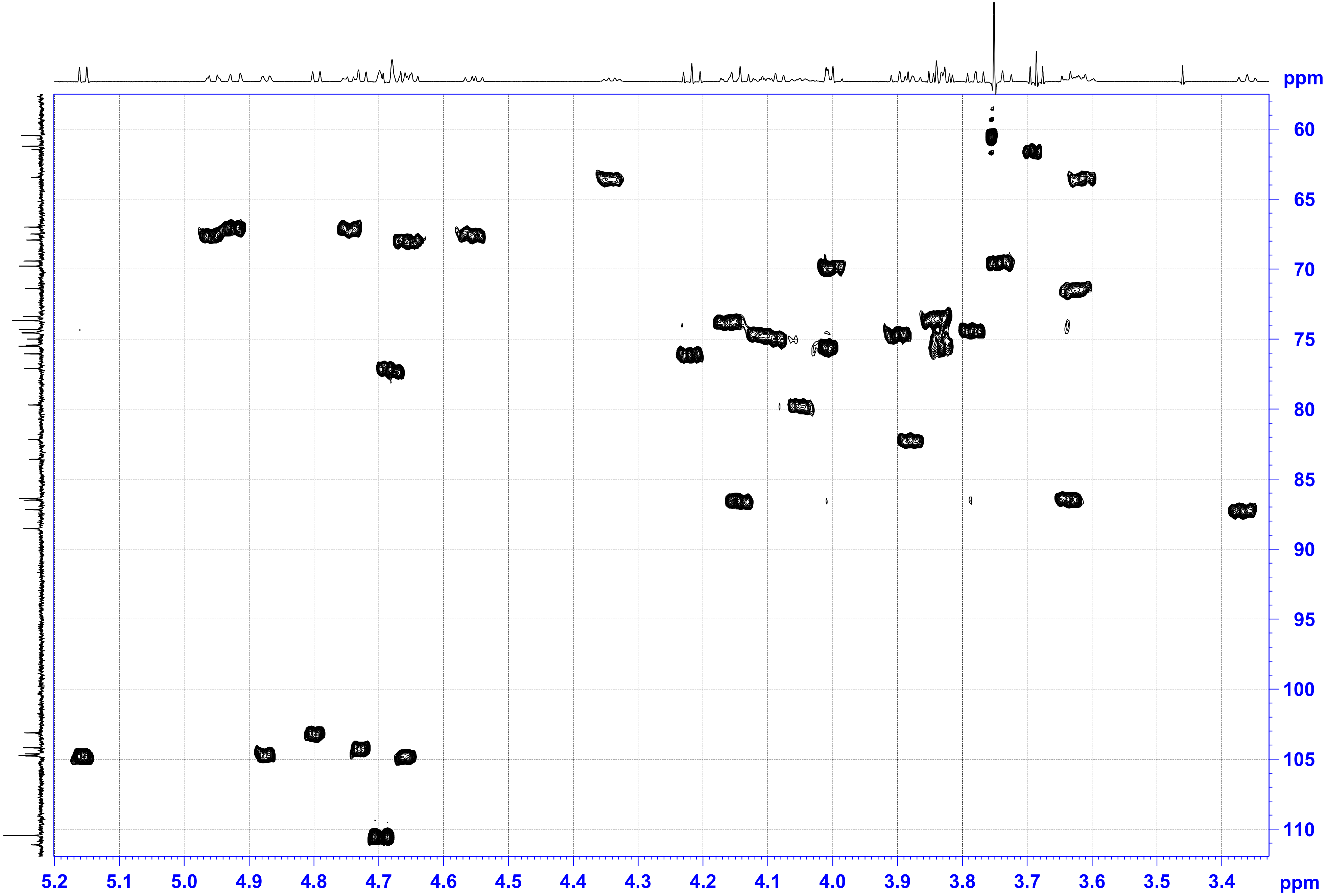

Figure 48. The HSQC (700.13 MHz) spectrum of the carbohydrate moiety of **9** in C_5D_5N/D_2O (4/1).

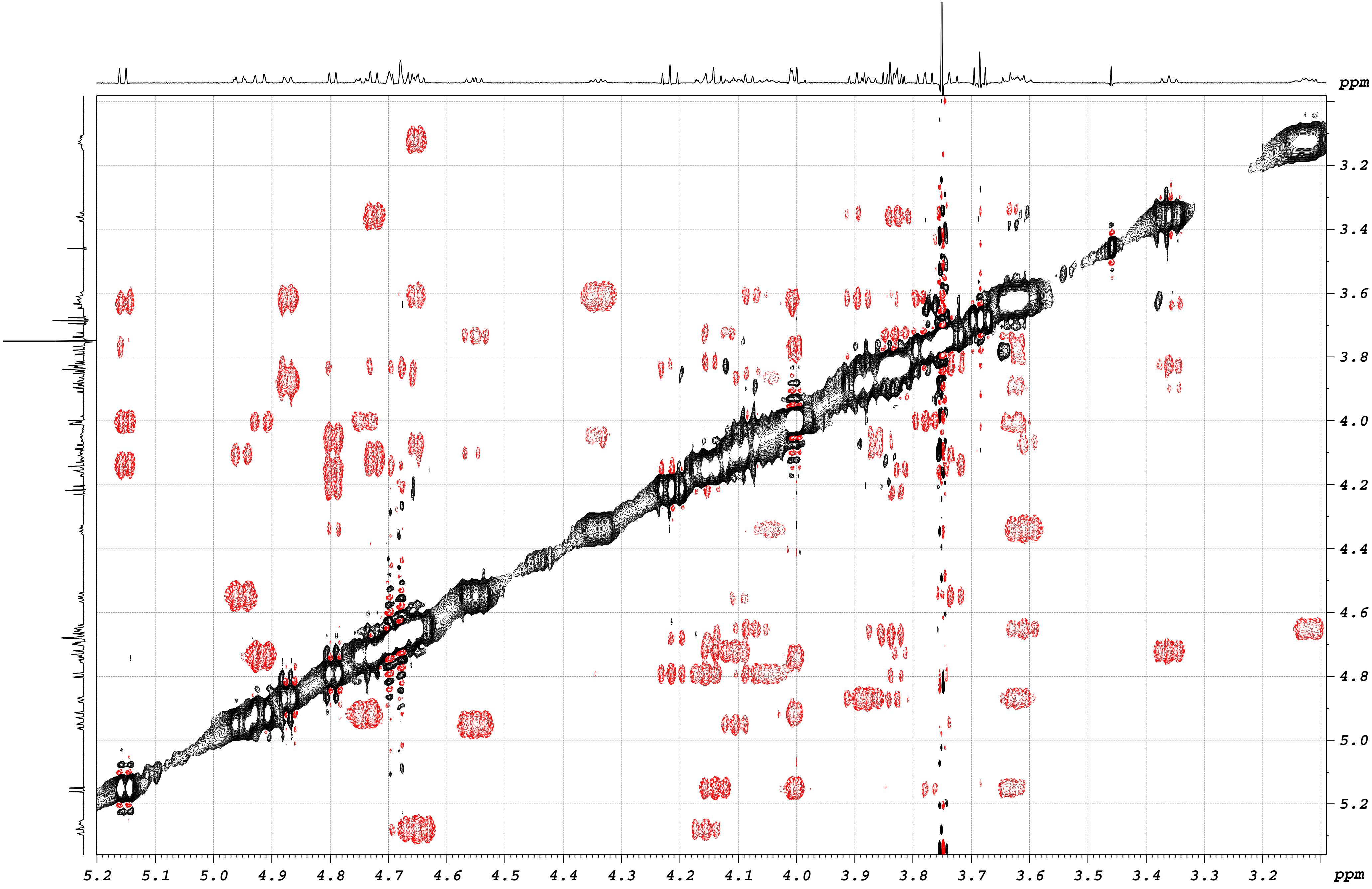

Figure 49. The ROESY (500.13 MHz) spectrum of the carbohydrate moiety of **9** in C_5D_5N/D_2O (4/1).

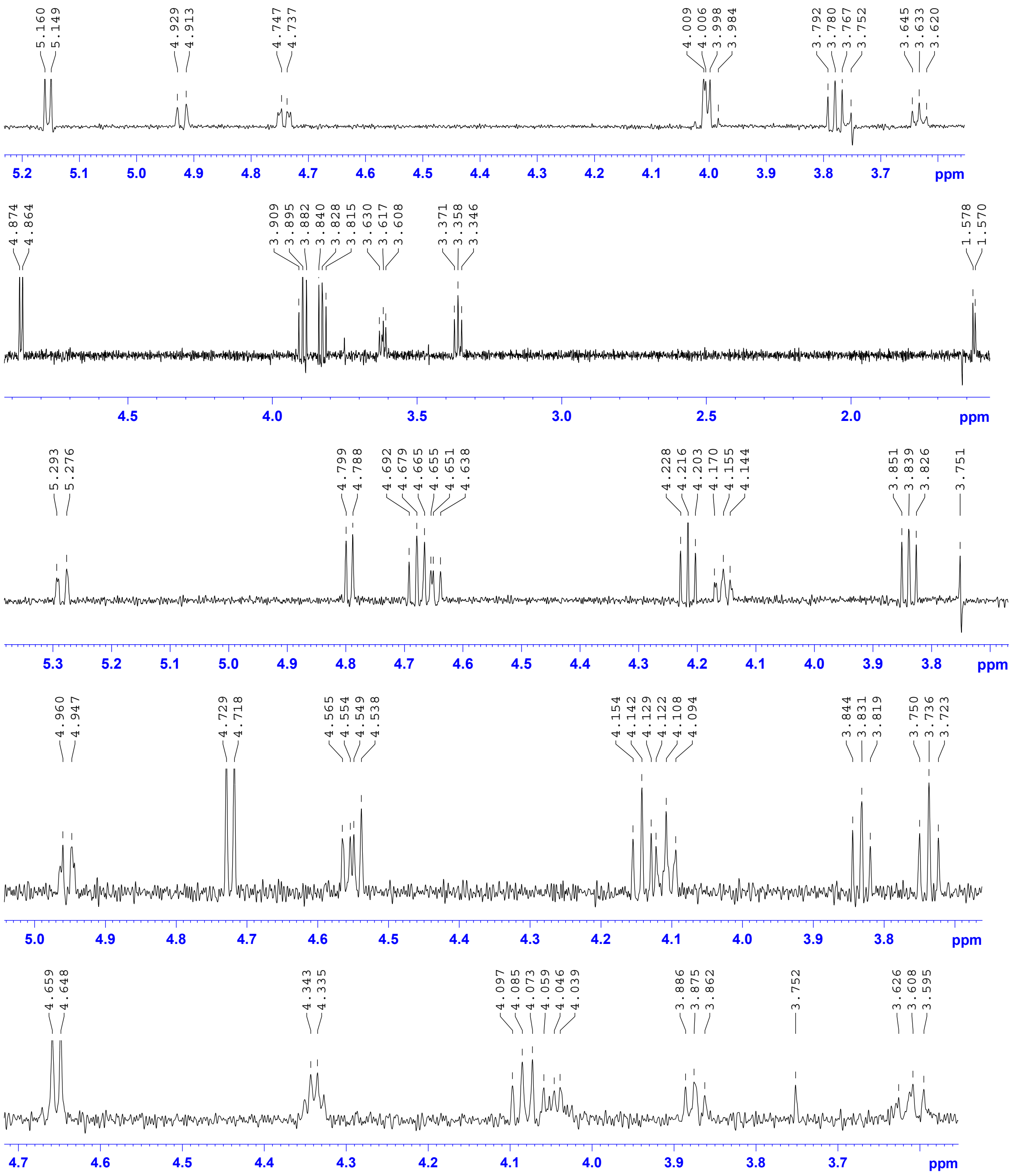

Figure 50. 1D TOCSY (700.13 MHz) spectra of the monosaccharide residues in **9** in C_5D_5N/D_2O (4/1).

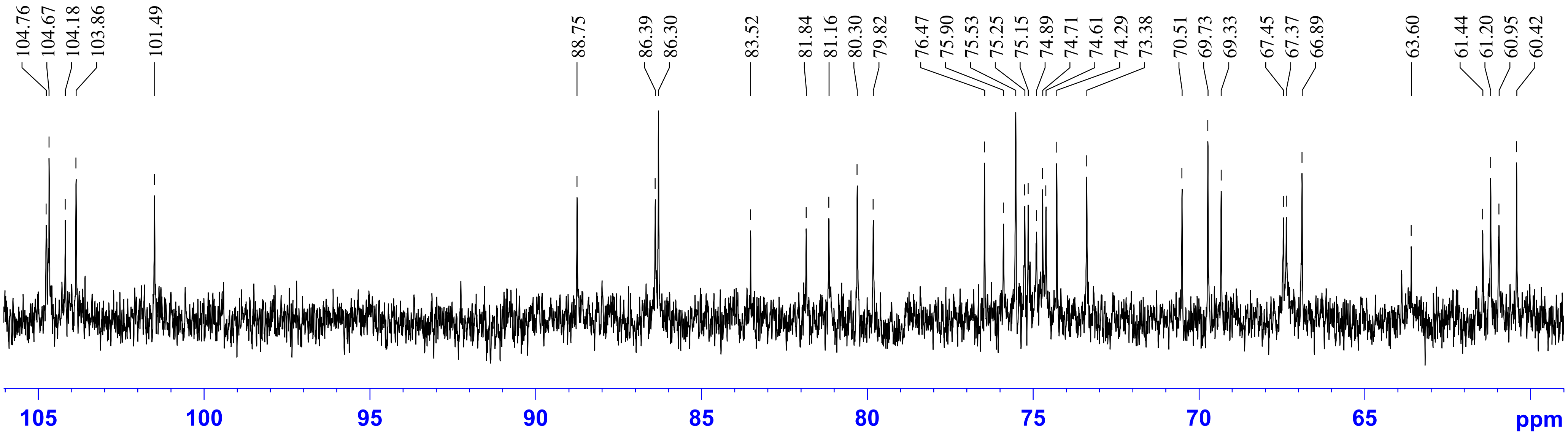

Figure 51. The ^{13}C NMR (176.04 MHz) spectrum of the carbohydrate moiety of **10** in C_5D_5N/D_2O (4/1).

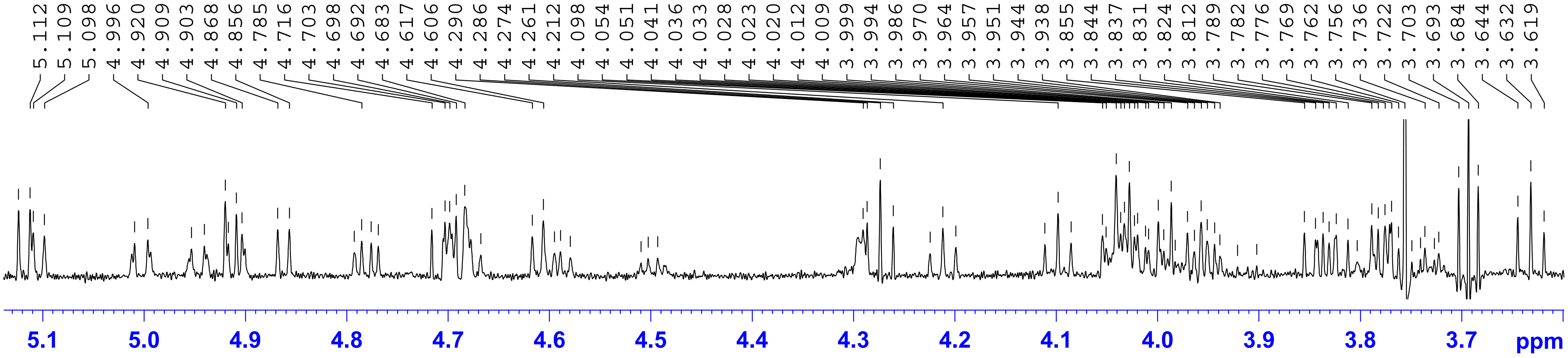

Figure 52. The ^{1}H NMR (700.13 MHz) spectrum of the carbohydrate moiety of **10** in C_5D_5N/D_2O (4/1).

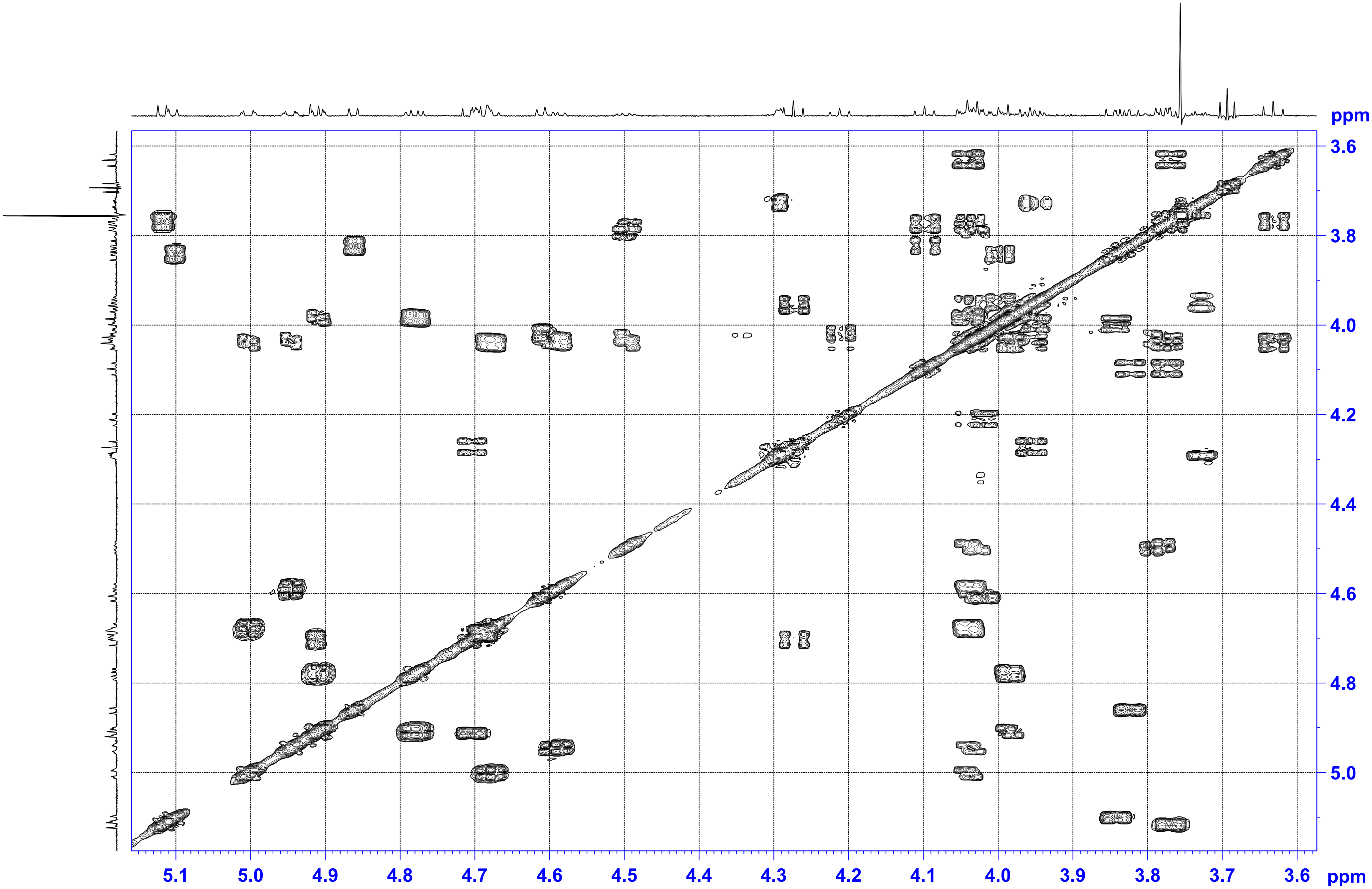

Figure 53. The COSY (700.13 MHz) spectrum of the carbohydrate moiety of **10** in C_5D_5N/D_2O (4/1).

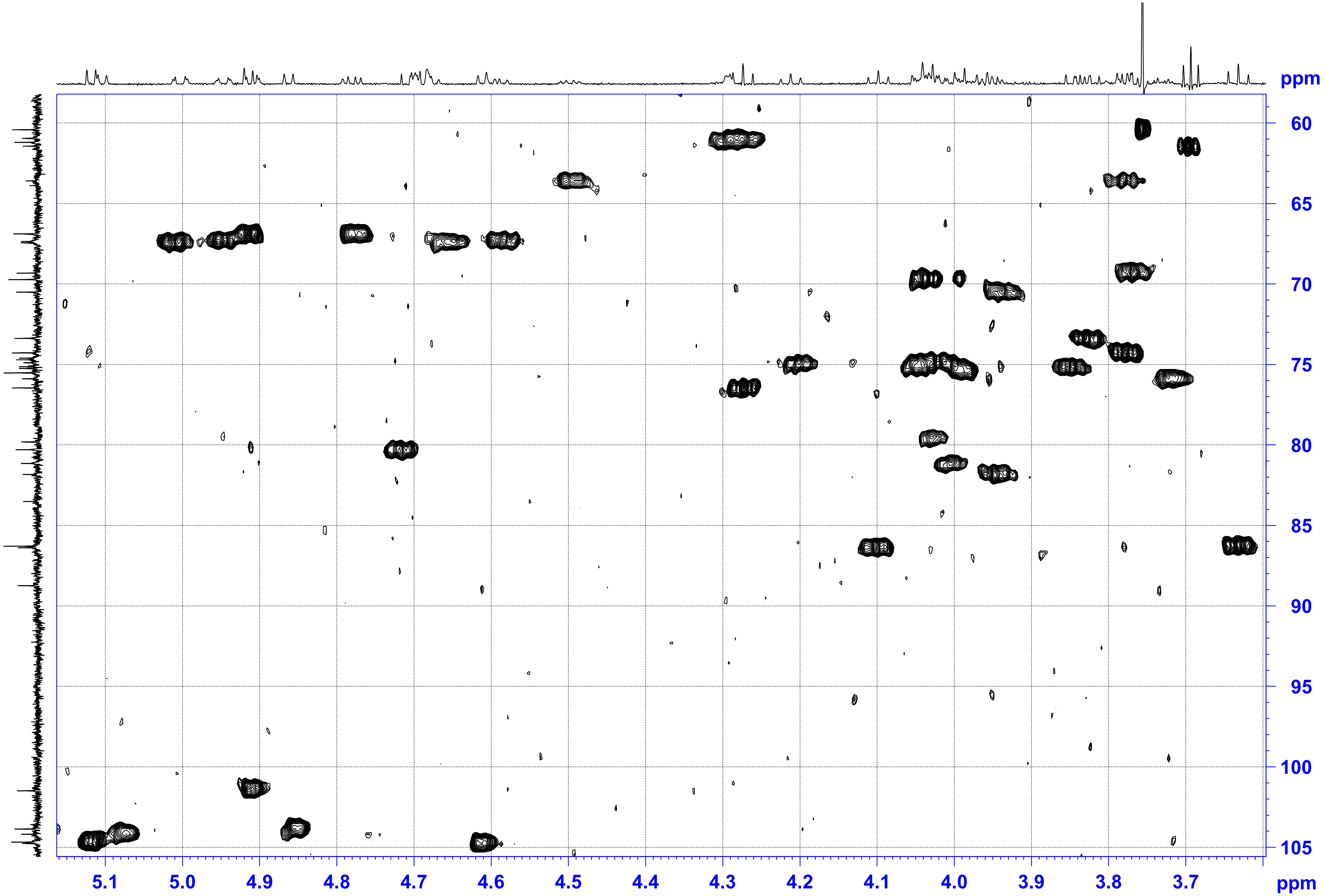

Figure 54. The HSQC (700.13 MHz) spectrum of the carbohydrate moiety of **10** in C_5D_5N/D_2O (4/1).

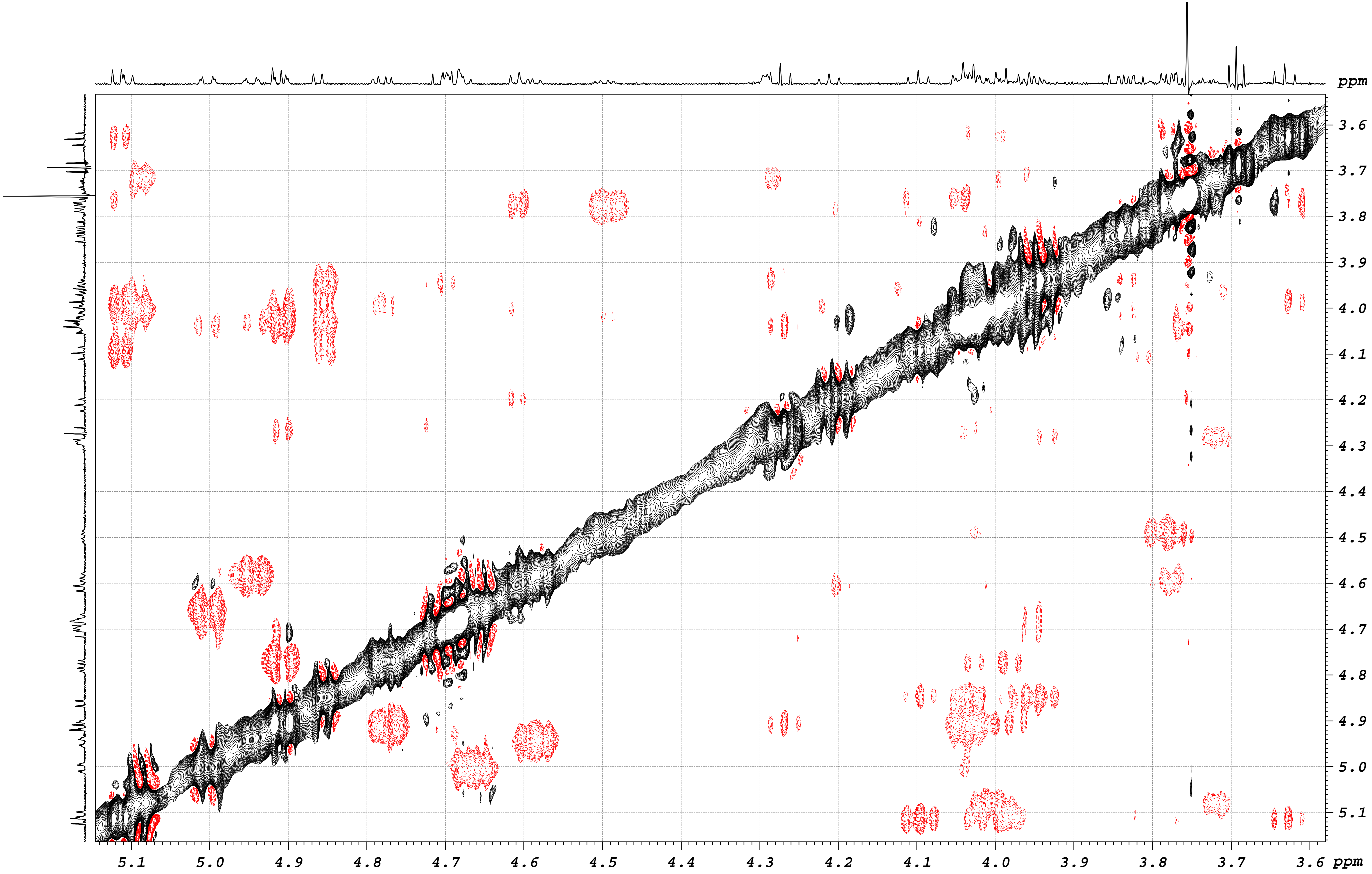

Figure 55. The ROESY (500.13 MHz) spectrum of the carbohydrate moiety of **10** in C_5D_5N/D_2O (4/1).

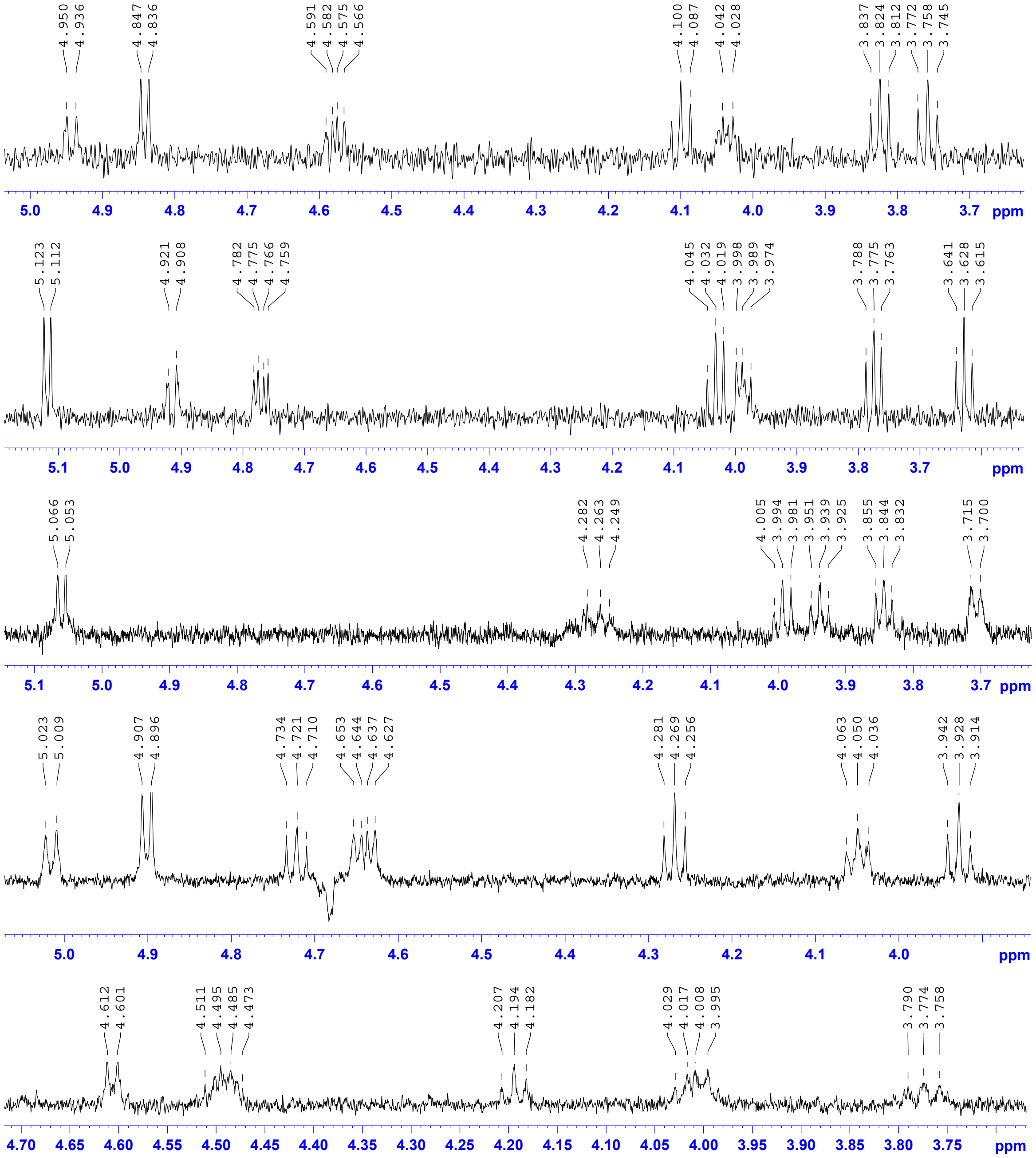

Figure 56. 1D TOCSY (700.13 MHz) spectra of the monosaccharide residues in **10** in C_5D_5N/D_2O (4/1).

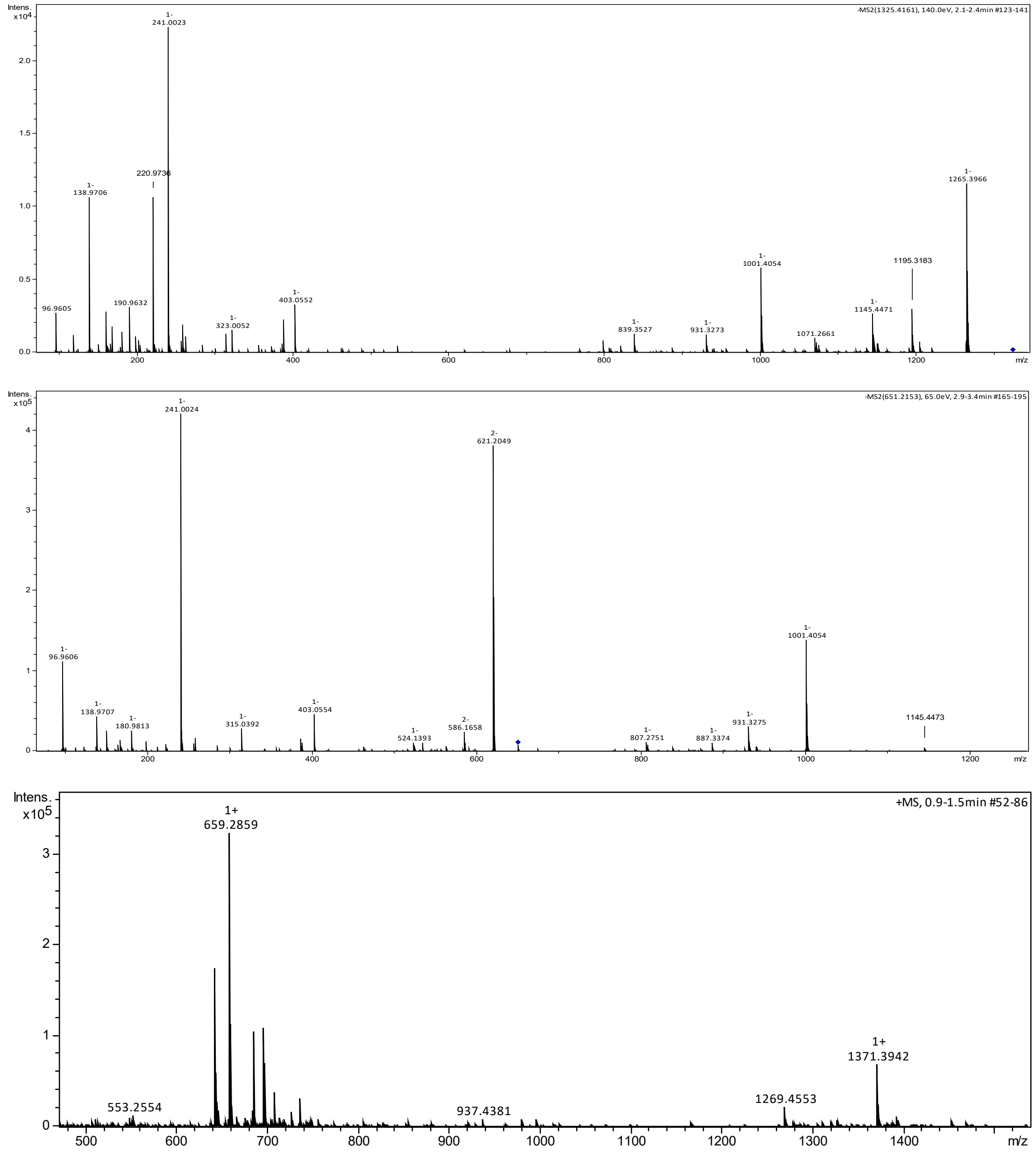

Figure 57. ESI-MS (–) spectra of psolusoside B₁ (**1**).

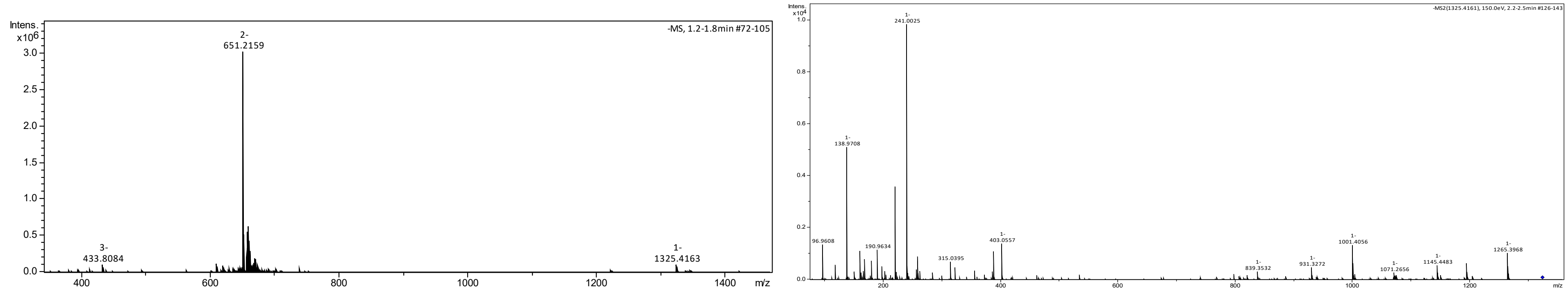

Figure 58. ESI-MS (–) spectra of psolusoside B$_2$ (**2**).

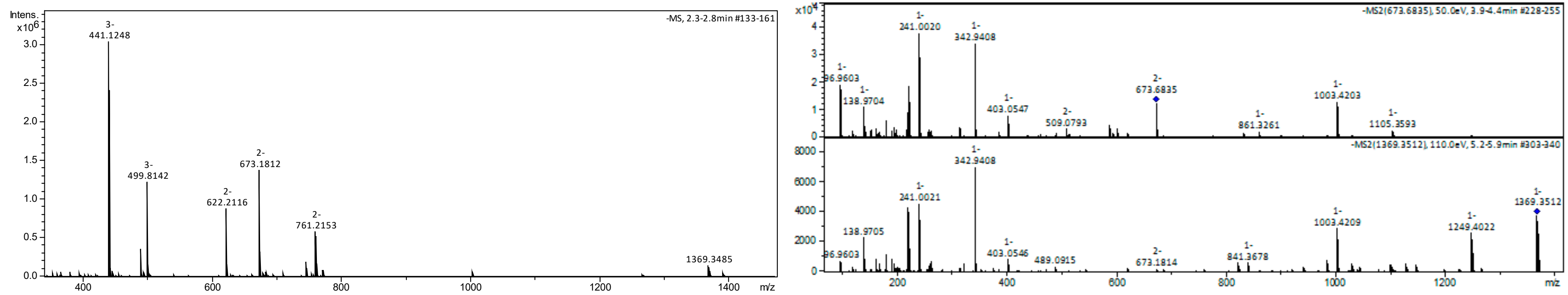

Figure 59. ESI-MS (–) spectra of psolusoside J (**3**).

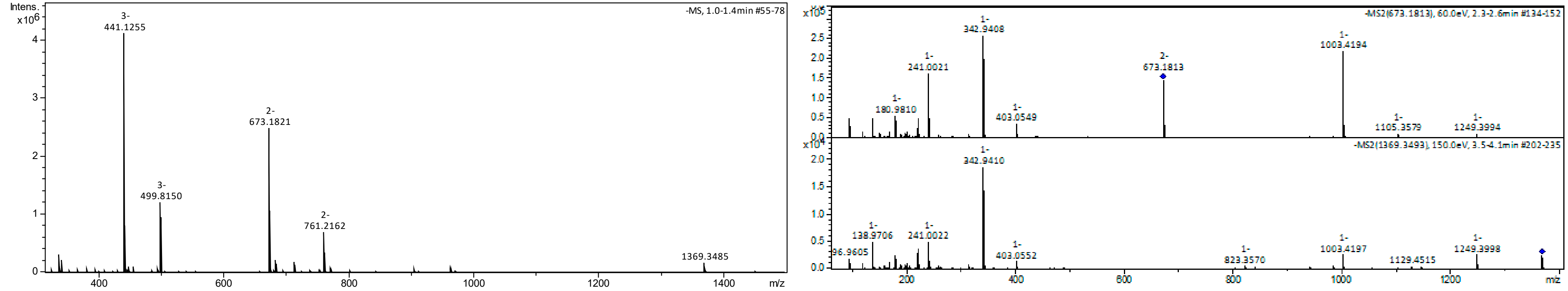

Figure 60. ESI-MS (–) spectra of psolusoside K (**4**).

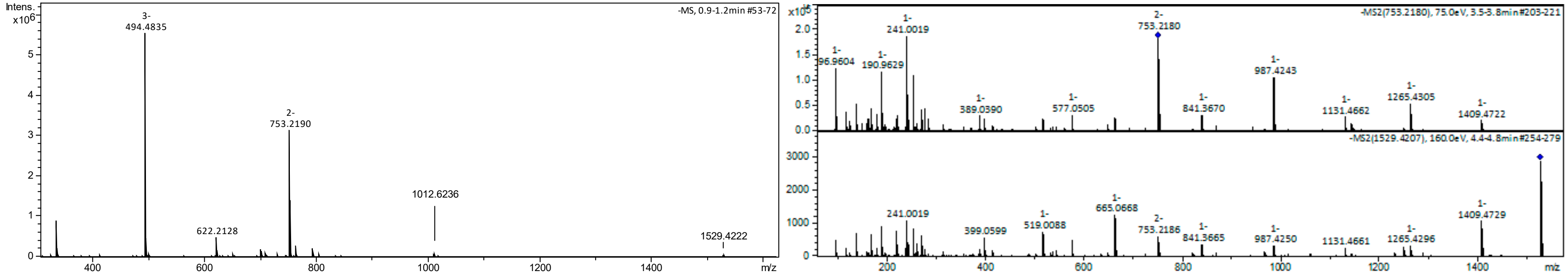

Figure 61. ESI-MS (–) spectra of psolusoside L (**5**).

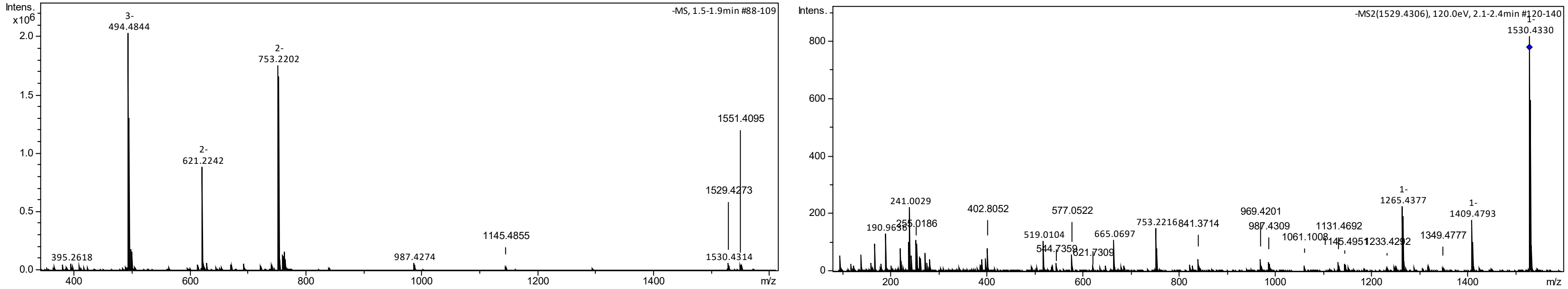

Figure 62. ESI-MS (–) spectra of psolusoside M (**6**).

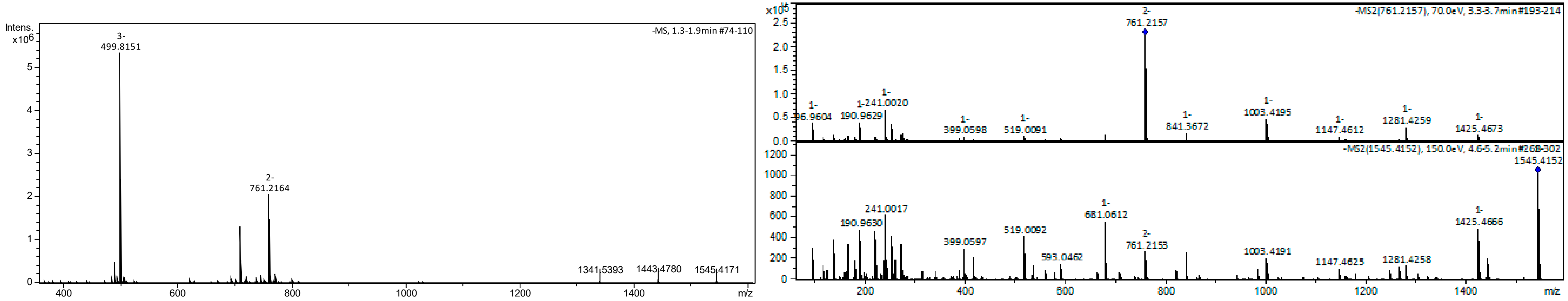

Figure 63. ESI-MS (–) spectra of psolusoside N (**7**).

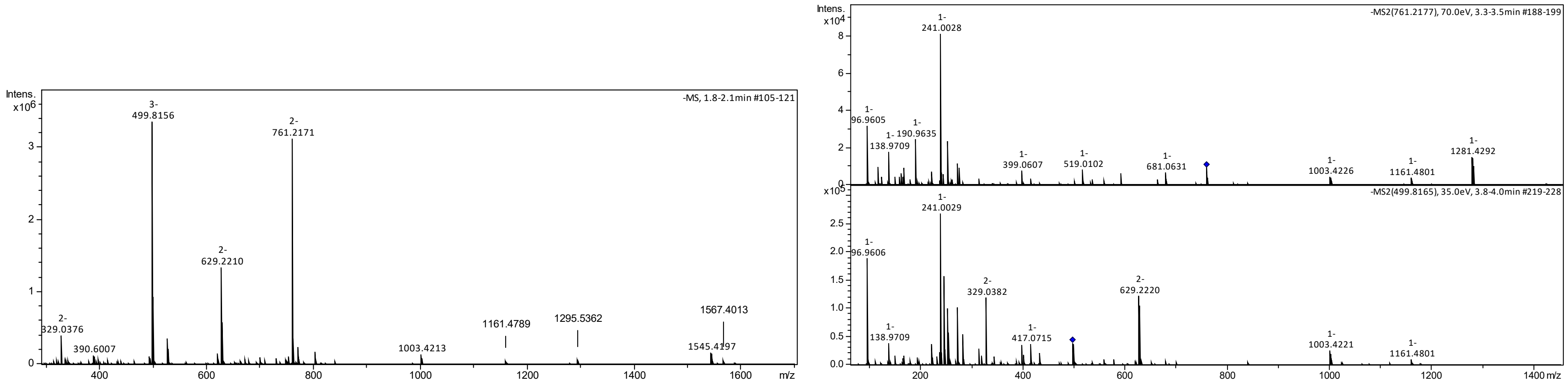

Figure 64. ESI-MS (–) spectra of psolusoside O (**8**).

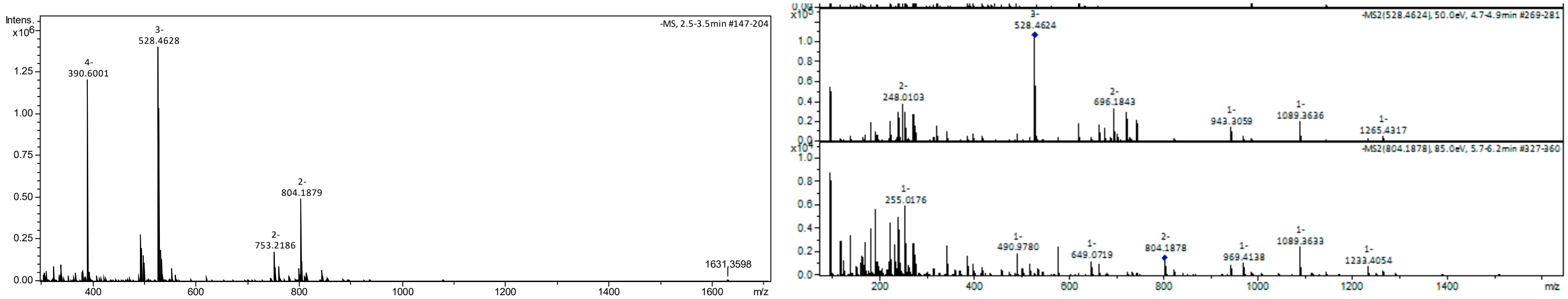

Figure 65. ESI-MS (–) spectra of psolusoside P (**9**).

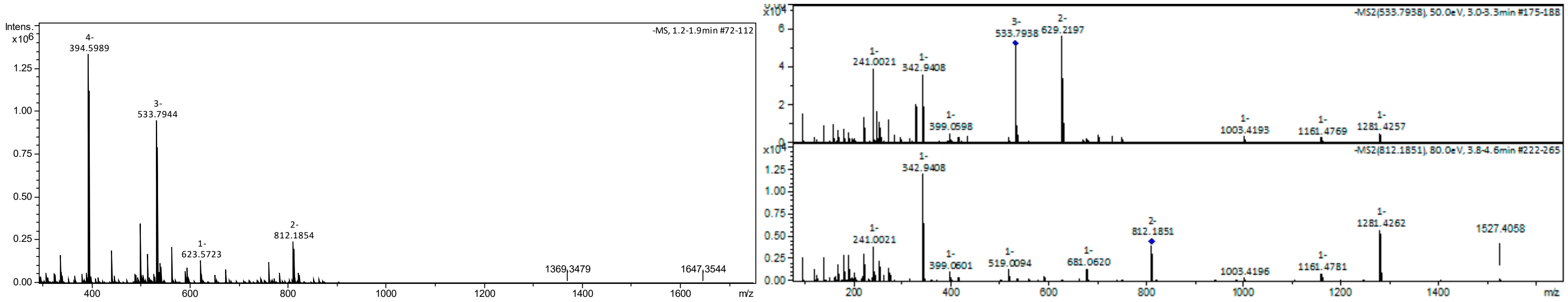

Figure 66. ESI-MS (–) spectra of psolusoside Q (**10**).
